# Simultaneous binding of Guidance Cues NET1 and RGM blocks extracellular NEO1 signaling

**DOI:** 10.1016/j.cell.2021.02.045

**Published:** 2021-04-15

**Authors:** Ross A. Robinson, Samuel C. Griffiths, Lieke L. van de Haar, Tomas Malinauskas, Eljo Y. van Battum, Pavol Zelina, Rebekka A. Schwab, Dimple Karia, Lina Malinauskaite, Sara Brignani, Marleen H. van den Munkhof, Özge Düdükcü, Anna A. De Ruiter, Dianne M.A. Van den Heuvel, Benjamin Bishop, Jonathan Elegheert, A. Radu Aricescu, R. Jeroen Pasterkamp, Christian Siebold

**Affiliations:** 1 Division of Structural Biology, Wellcome Centre for Human Genetics, University of Oxford, Roosevelt Drive, Oxford OX3 7BN, UK; 2 Department of Translational Neuroscience, UMC Utrecht Brain Center, University Medical Center, Utrecht University, Universiteitsweg 100, 3584 CG Utrecht, the Netherlands; 3 MRC Laboratory of Molecular Biology, Cambridge Biomedical Campus, Francis Crick Avenue, Cambridge CB2 0QH, UK

**Keywords:** signal transduction, cell surface receptors, axon regeneration, cell migration, Netrin, Neogenin, repulsive guidance molecule, complex structure, protein-protein interactions, morphogen signaling

## Abstract

During cell migration or differentiation, cell surface receptors are simultaneously exposed to different ligands. However, it is often unclear how these extracellular signals are integrated. Neogenin (NEO1) acts as an attractive guidance receptor when the Netrin-1 (NET1) ligand binds, but it mediates repulsion via repulsive guidance molecule (RGM) ligands. Here, we show that signal integration occurs through the formation of a ternary NEO1-NET1-RGM complex, which triggers reciprocal silencing of downstream signaling. Our NEO1-NET1-RGM structures reveal a “trimer-of-trimers” super-assembly, which exists in the cell membrane. Super-assembly formation results in inhibition of RGMA-NEO1-mediated growth cone collapse and RGMA- or NET1-NEO1-mediated neuron migration, by preventing formation of signaling-compatible RGM-NEO1 complexes and NET1-induced NEO1 ectodomain clustering. These results illustrate how simultaneous binding of ligands with opposing functions, to a single receptor, does not lead to competition for binding, but to formation of a super-complex that diminishes their functional outputs.

## Highlights

• The NEO1-NET1 structure suggests NET1-mediated NEO1/DCC cell surface clustering
• The receptor NEO1 and its ligands NET1 and RGM form a trimer-of-trimers supercomplex
• NET1 inhibits RGMA-induced growth cone collapse via the NEO1-NET1-RGM complex
• The NEO1-NET1-RGM complex silences RGM and NET1 effects on neuron migration

## Graphical abstract

**Figure:**
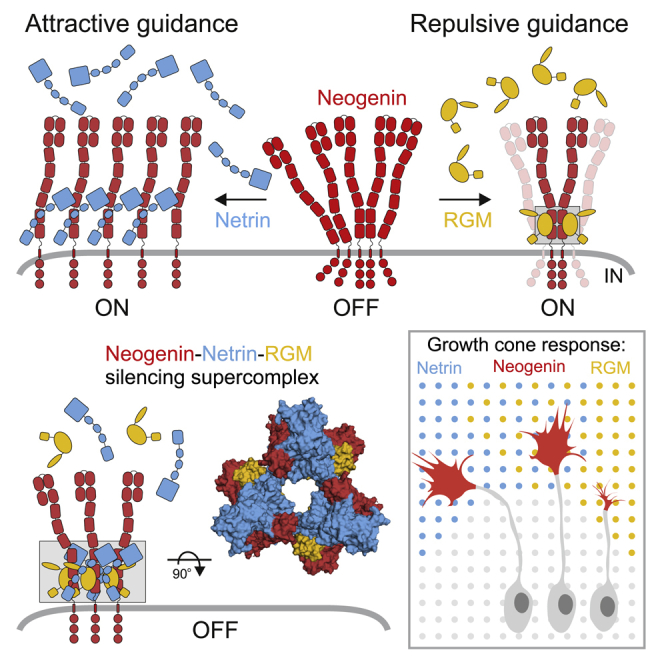

## Teaser

When extracellular guidance molecules Netrin and RGM that have opposing functions simultaneously bind the Neogenin receptor, a super-complex is formed that diminishes their functional outputs.

## Introduction

During their lifespan, cells are exposed to a plethora of cues that control processes such as cell division, differentiation, migration, and death. Often, these cues are presented simultaneously to bind specific cell surface receptors and activate downstream signaling pathways. Despite recent progress, how this wealth of extracellular information is integrated and controlled remains poorly understood. A remarkable example of such integration is represented by the receptor Neogenin (NEO1) and its two ligands, repulsive guidance molecule (RGM) and Netrin-1 (NET1). NEO1 is a single-pass transmembrane receptor belonging to the immunoglobulin (Ig) superfamily (Wilson and Key, 2007), composed of 4 N-terminal Ig-like domains, followed by 6 fibronectin type III-like domains, a single transmembrane helix, and an intracellular domain. It is implicated in inflammation (Fujita and Yamashita, 2017), multiple sclerosis (Demicheva et al., 2015), and various cancers (Li et al., 2009), and it has crucial functions in diverse cellular processes ranging from cell motility and adhesion (e.g., axon guidance, vascular development) (De Vries and Cooper, 2008; Kang et al., 2004) to survival and differentiation (O’Leary et al., 2015). To mediate these functions, NEO1 binds structurally and functionally distinct ligands such as RGM and NET1.

Interactions between NEO1 and all three members of the GPI-anchored RGM family (RGMA, RGMB/Dragon, RGMC/Hemojuvelin/HFE2) are mediated by FN domains 5 and 6 of NEO1, forming the core of a signal transduction hub for RGM-mediated repulsive guidance through the plasma membrane. Two RGM molecules act as molecular staples to bring together the juxtamembrane regions of two NEO1 receptors into a signaling-competent dimer (Bell et al., 2013; Rajagopalan et al., 2004; Siebold et al., 2017). Binding of RGM to NEO1 triggers rearrangements of the cytoskeleton via Rho GTPases and other effectors, and it results in, e.g., growth cone collapse (Conrad et al., 2007; Monnier et al., 2002). Whilst RGM-mediated signaling is specific to NEO1, NET1 can signal via both NEO1 and deleted in colorectal cancer (DCC), a structural homologue of NEO1 (Huyghe et al., 2020; Keino-Masu et al., 1996; Wilson and Key, 2006; Xu et al., 2014). Secreted netrins comprise an N-terminal laminin (LN) domain followed by three laminin epidermal growth factor-like repeats (LE1-3) and a C-terminal Netrin (NTR) domain (Cirulli and Yebra, 2007). NEO1 and DCC bind NET1 to trigger attractive growth cone responses and other cellular effects (Geisbrecht et al., 2003; Keino-Masu et al., 1996; Mille et al., 2009; Wang et al., 1999). Structural studies on binary NET1-DCC and NET1-NEO1 complexes have identified the three NEO1 membrane-proximal FN domains as the NET1 binding sites (Finci et al., 2014; Xu et al., 2014). This interaction triggers rearrangements of the actin cytoskeleton (Li et al., 2008; Li et al., 2002; Stavoe and Colón-Ramos, 2012).

NET1 and RGMs show partially overlapping expression patterns in different tissues, including the neural tube and nervous system. Both NEO1 ligands have been implicated in similar cellular processes, such as neuron migration (O’Leary et al., 2013; O’Leary et al., 2015; van Erp et al., 2015), axon guidance (Monnier et al., 2002; Wilson and Key, 2006; Xu et al., 2014), and inflammation (Fujita and Yamashita, 2017; Mirakaj and Rosenberger, 2017). At the cellular level, NET1 and RGMs cause opposing effects via NEO1 (e.g. cell attraction versus repulsion). As both ligands bind to the NEO1 FN domains, their signaling needs to be spatiotemporally controlled. Simultaneous exposure of NEO1 to RGMs and NET1 occurs during neural tube closure, axon guidance, neuron migration, and other processes (Kee et al., 2008; Moon et al., 2011; Muramatsu et al., 2011; O’Leary et al., 2013; Wilson and Key, 2006), and NET1 and RGMs can functionally interact. For example, RGMA is a repulsive guidance cue for NEO1-positive cortical interneurons migrating out of the medial ganglionic eminence (MGE) of the ventral forebrain. Interestingly, NET1 expression in the MGE silences the repulsive effects of RGMA-NEO1 signaling (O’Leary et al., 2013). Despite these insights, the molecular mechanisms explaining how both ligands activate NEO1, and how these guidance cues cross-talk to transduce their signals, remain elusive.

Here, we determined the structure of an unexpected ternary NEO1-NET1-RGM complex that forms a defined and stable “trimer-of-trimers” assembly. We show that formation of the ternary NEO1-NET1-RGM complex inhibits both RGMA-NEO1-mediated growth cone collapse and RGMA-NEO1- and NET1-NEO1-mediated neuron migration. Our data demonstrate that NET1 and RGMs can bind simultaneously to NEO1 rather than competing for binding, thereby unveiling an intriguing mode of receptor regulation. The ternary structure acts as a silencing complex, preventing the formation of the signaling-compatible RGM-NEO1 complex as well as NET1-induced NEO1 ectodomain clustering, both required for signal transduction. Thus, our work reveals how two ligands with distinct and opposing cellular functions can control their receptor by reciprocal silencing of downstream signaling pathways. This mechanism is likely to apply more generally, as other receptors are also known to bind structurally and functionally distinct ligands.

## Results

### RGMs and NET1 bind simultaneously to their receptor NEO1 at the cell surface

**Figure S1:**
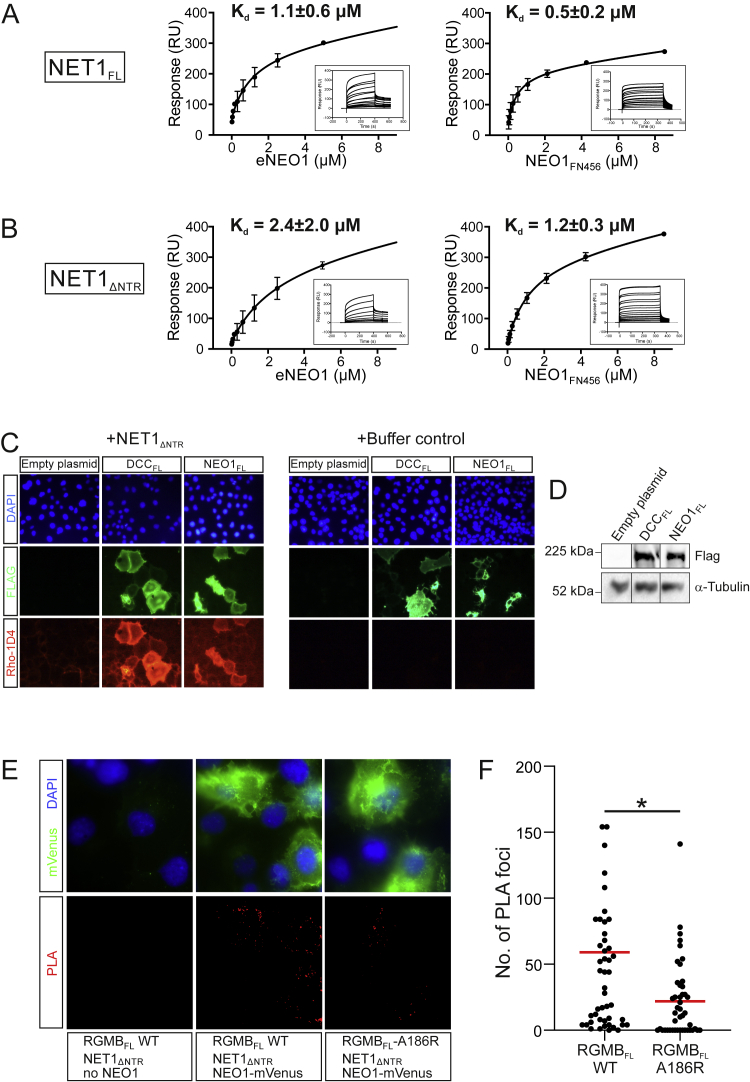
Identification of the minimal NEO1-NET1 interaction region, related to Figure 1 **(A, B)** SPR equilibrium binding experiments of different NET1 and NEO1 constructs. Graphs show a plot of the equilibrium binding response against used NEO1 construct concentrations (left panels: full-length NEO1 ectodomain (eNEO1), right panels: NEO1 FN type III domains 4 to 6 (NEO1_FN456_). Ligands immobilized on SPR sensor chip: **A**, full-length NET1; **B**, NET1_ΔNTR_. **(C)** Immunofluorescence staining of FLAG-tagged full-length human DCC (DCC_FL_) and mouse NEO1 (NEO1_FL_) overexpressed in COS-7 cells (green). Left panel: bound NET1_ΔNTR_ is stained via a Rho ID4 tag (red); right panel: transfected cells were incubated with buffer only as a negative control and stained as in the left panel. **(D)** Western blot of COS-7 cells transfected with the indicated plasmids used in **C**. α-tubulin serves as a loading control. **(E, F)** Proximity ligation assay (PLA) to test for simultaneous binding of NET1 and RGMB to NEO1. **(E)** COS-7 cells were transfected with a NEO1-mVenus fusion protein or the corresponding empty vector, and with full-length RGMB (wild type or RGMB-A186R). Transfected cells were incubated with NET1_ΔNTR_ before performing the PLA assay. PLA signals are shown in red and NEO1-mVenus transfected cells in green with nuclei in blue. **(F)** PLA signals were quantified and values from 3 individual experiments were plotted. A two-tailed, unpaired t test showed the statistical significance as p = 0.0107.

**Figure 1:**
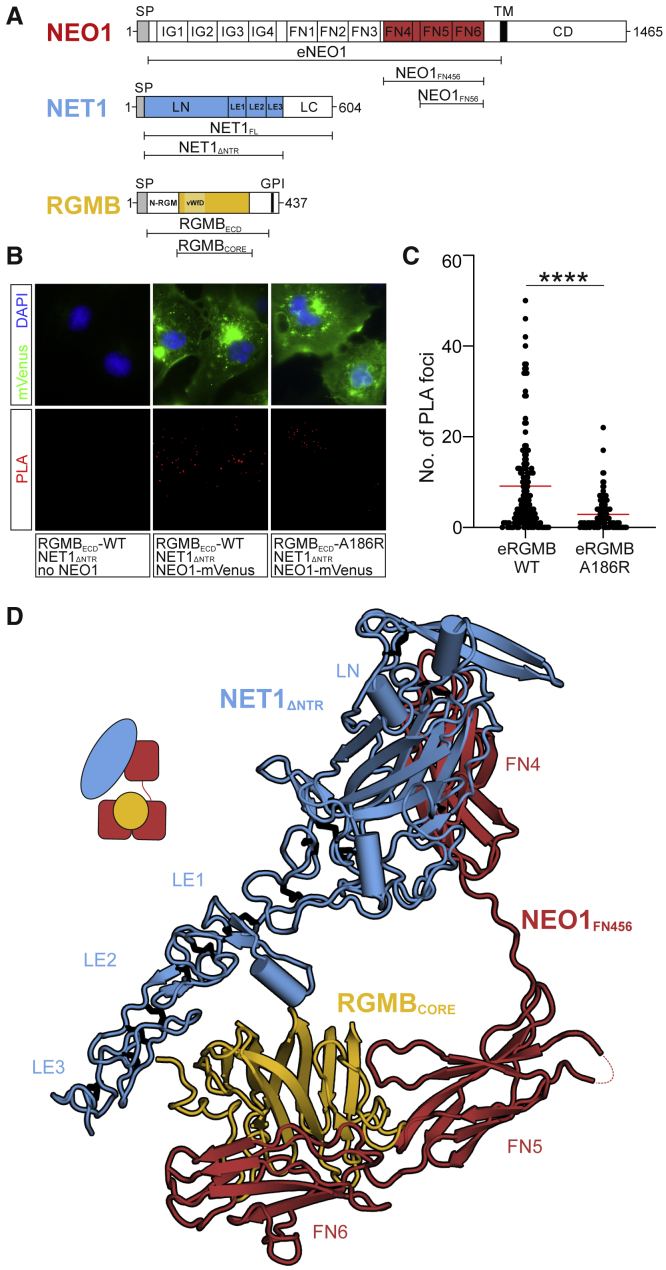
NET1 and RGMB can simultaneously bind NEO1 and form a ternary complex (A) Schematics of NEO1, NET1, and RGMB. SP, signal peptide; TM, transmembrane helix; IG, immunoglobulin-like domain; FN, fibronectin type III domain; CD, cytoplasmic domain; LN, laminin domain; LE, laminin epidermal growth factor-like repeats; LC, netrin (NTR) domain; N-RGM, RGM N-terminal domain identified to bind to BMP ligands (Healey et al., 2015); vWfD, von Willebrand factor D-like domain; GPI, glycosylphosphatidylinositol anchor. (B and C) Proximity ligation assays (PLA) were performed to test for simultaneous binding of NET1 and RGMB to NEO1. (B) Cos-7 cells were either transfected with a NEO1-mVenus fusion protein or empty vector. Cells were incubated with NET1_ΔNTR_ and RGMB_ECD_ (wild type or RGMB_ECD_-A186R). NEO1-mVenus positive cells are shown in green, nuclei are stained with DAPI and PLA signals in red. (C) Number of PLA signals per NEO1-mVenus positive cells. Individual values are plotted from 4 independent experiments. Statistical significance was determined using a two-tailed, unpaired t test with p < 0.0001. (D) Ribbon representation of the NEO1-NET1-RGMB protomer observed in the 3.25 Å resolution crystal structure, with NEO1_FN456_ in red, NET1_ΔNTR_ in blue and RGMB_CORE_ in yellow. A schematic is shown. See also Figure S1.

To dissect the molecular mechanisms that control NEO1 signaling via interactions with RGM and NET1, we identified the minimal binding regions for the NET1-NEO1 interactions using surface plasmon resonance (SPR) and cell surface binding experiments (Figures S1A–S1D). Our data show that the minimal complex is composed of the three membrane proximal FN domains of NEO1 (NEO1_FN456_) and a NET1 construct lacking the C-terminal NTR domain (NET1_ΔNTR_) (Figure 1A), in line with previous biochemical studies (Geisbrecht et al., 2003; Kruger et al., 2004; Mille et al., 2009; Xu et al., 2014). We previously mapped the RGM-binding region on NEO1 to the two membrane-proximal FN domains (NEO1_FN56_) (Bell et al., 2013).

Since cells can simultaneously encounter RGMs and NET1 in a NEO1-dependent manner *in vivo* (Kee et al., 2008; Moon et al., 2011; Muramatsu et al., 2011; O’Leary et al., 2013; Wilson and Key, 2006), we asked whether a stable ternary NEO1-NET1-RGM complex can exist at the cell surface, or whether NET1 and RGM would compete for NEO1 binding. To distinguish between these two options, we carried out *in situ* proximity ligation assays (PLAs) (Söderberg et al., 2006). We mutually incubated full-length NEO1 expressing cells with purified NET1_ΔNTR_ and the full-length ectodomain of RGMB (RGMB_ECD_) and observed numerous PLA foci, which can only be generated when NET1 and RGMB come into close proximity (<40nm) (Figures 1B and 1C). The number of foci was diminished when wild type RGMB was replaced with a RGMB mutant (RGMB-A186R [Healey et al., 2015]) that cannot efficiently bind to NEO1 (Figures 1B and 1C). This result suggests that NEO1 is specifically required for bridging NET1 and RGMB and that a complex of RGMB, NET1, and NEO1 forms on the cell surface. This scenario describes signaling in “*trans*,” whereby RGM and NEO1 are expressed in different cells, for example in RGM-mediated axon guidance. We also observed a high number of foci from NET1-RGMB interactions when full-length RGMB (RGMB_FL_) and NEO1 are co-transfected in the presence of soluble NET1_ΔNTR_ (Figures S1E and S1F). This shows that the ternary complex can also form in “*cis*,” a situation occurring in hepatocytes or chondrocytes (Zhang et al., 2009; Zhou et al., 2010) where the RGM-NEO1 complex regulates BMP signaling. It remains to be seen what the effect of NET1 in these signaling scenarios is.

### The structure of the ternary NEO1-NET1-RGM complex reveals a “trimer-of-trimers”

**Figure 2:**
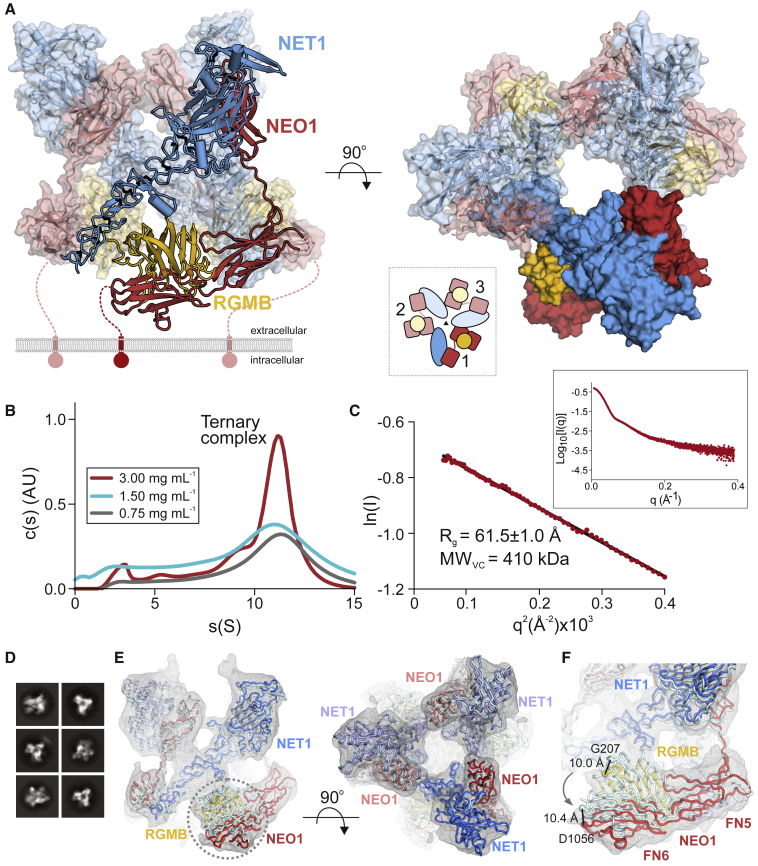
Structure of the NEO1-NET1-RGMB ternary complex (A) Two 90°-rotated ribbon representations of the NEO1-NET1-RGMB trimer-of-trimers complex. The relative location of the plasma membrane is depicted. The solvent accessible surface is shown in the right panel in addition to the ribbons. The inset shows an outline complex architecture and symmetry. Disordered regions are depicted as dotted lines. Color-coding is as in Figure 1D. (B) Sedimentation velocity AUC experiment of the NEO1_FN456_-NET1_ΔNTR_-RGMB_ECD_ complex at different concentrations. The major species corresponds to a 3:3:3 complex. (C) Guinier region analysis of the NEO1-NET1-RGMB complex from SEC-SAXS experiment suggests a molecular weight of 410 kDa, corresponding to the 3:3:3 NEO1_FN456_:NET1_ΔNTR_:RGMB_ECD_ stoichiometry. The inset shows the SAXS intensity plot for the final merged data. (D) Selected 2D class averages used for cryo-EM map reconstruction of the NEO1-NET1-RGMB ternary complex. (E) Ribbon representation of the 3:3:3 NEO1-NET1-RGMB cryo-EM complex. View and coloring as in (A). The crystallographic 3:3:3 NEO1-NET1-RGMB complex fitted into the cryo-EM map as a single rigid body (depicted in light cyan) is shown for comparison. The cryo-EM electron potential (grey mesh) is calculated to 6.0 Å resolution. (F) Close-up view of the NEO1-RGMB interface highlighted in (**E)**. The model of the ternary 3:3:3 complex fits the cryo-EM map better when refined as six rigid bodies (see also STAR Methods). Distances (Å) between selected Cα atoms are indicated. The curved arrow highlights the movement of the NEO1_FN56_-RGMB segment by up to 10 Å relative to the NEO1_FN4_-NET1 segment. See also Figures S2 and S3 and Methods S1 and S2.

To understand how NEO1, NET1, and RGMB assemble into a ternary complex, we determined the crystal structure of the minimal ternary NEO1-NET1-RGMB complex, composed of NEO1_FN456_, NET1_ΔNTR_ and the RGMB NEO1-binding region (RGMB_CORE_) (Figure 1D; Methods S1; Table S1) to 3.25 Å resolution. Strikingly, this ternary complex is assembled into a 3:3:3 stoichiometry, composed of three 1:1:1 “protomer” complexes arranged around a 3-fold symmetry axis (Figure 1D and Figure 2A). To test whether such a “trimer-of-trimers” arrangement can exist in solution, we carried out analytical ultracentrifugation (AUC) (Figure 2B) and small angle X-ray scattering (SAXS) (Figure 2C) analyses. Both methods suggest the assembly of a 3:3:3 NEO1-NET1-RGMB complex as the major species.

**Figure S2:**
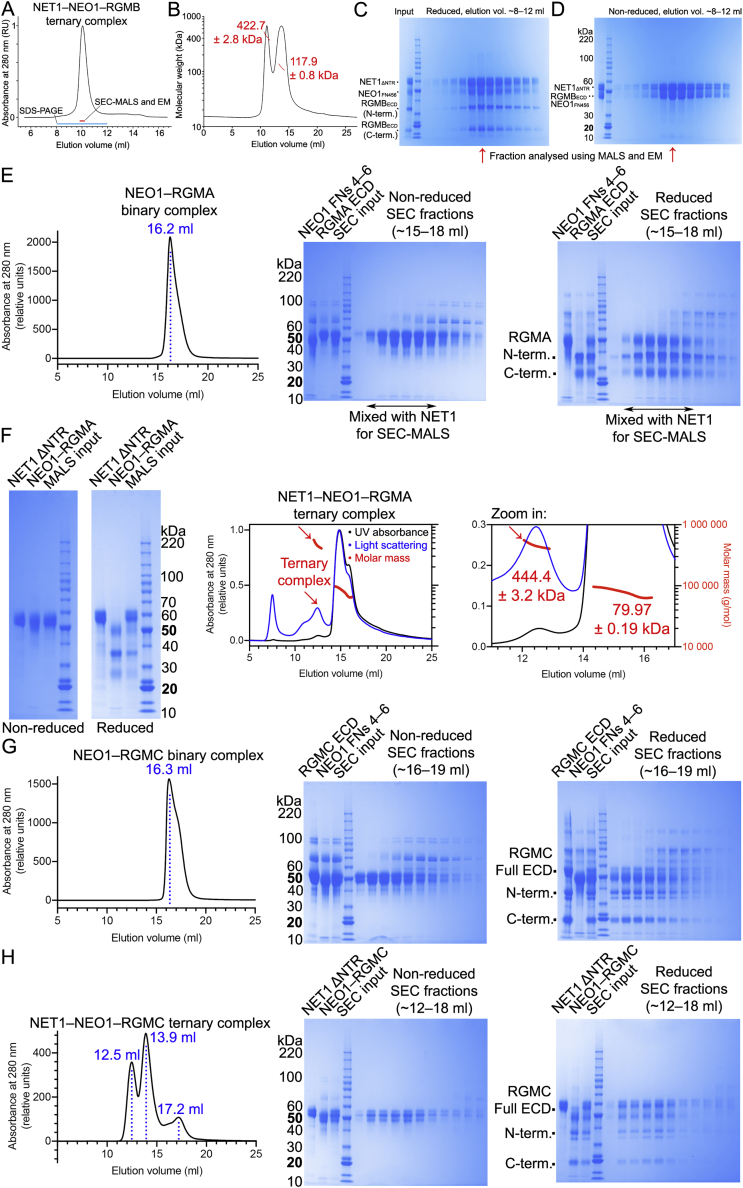
SEC, MALS and SDS-PAGE analysis of the ternary NEO1-NET1-RGM complexes, related to Figure 2 **(A)** SEC of the ternary NEO1_FN456_-NET1_ΔNTR_-RGMB_ECD_ complex. The SEC fraction (elution volume ~9.8-10.1 ml) indicated with a red line was analyzed using MALS (panel **B**) and cryo-EM. SEC fractions indicated with a blue line (elution volume ~8-12 ml) were analyzed on SDS PAGE (panels **C** and **D**). **(B)** SEC-MALS analysis of the NEO1_FN456_-NET1_ΔNTR_-RGMB_ECD_ complex. Calculated MW of 1:1:1 mol:mol:mol complex is 144.4 kDa (129.35 kDa of protein plus 15.06 kDa of seven Asn-linked Man_9_GIcNAc_2_ glycans). Calculated MW of 3:3:3 complex is 433.24 kDa. The NEO1-NET1-RGMB complex eluted as two peaks with corresponding MW of 422.7 kDa and 117.9 kDa (indicated with red lines). **(C, D)** SDS PAGE analysis of SEC fractions. Fractions were heated (100 °C, 10 minutes) in the presence or absence of 2-mercaptoethanol (panels **C** and **D**, respectively). **(E**) NEO1_FN456_ co-elutes with extracellular domain of RGMA (RGMA_ECD_) on SEC, suggesting that NEO1 and RGMA form a binary complex. SEC fractions were analyzed using SDS-PAGE under non-reducing and reducing conditions. Under reducing conditions, the RGMA_ECD_ dissociates into two fragments (labelled N-term. and C-term.) due to an autocatalytic cleavage mechanism. SEC fractions containing the binary NEO1-RGMA complex used to form the ternary NEO1-NET1-RGMA complex are indicated. SEC running buffer: 150 mM NaCl, 10 mM HEPES pH 7.5, 2 mM CaCl_2_, 0.02% NaN_3_ (flow rate 0.3 ml/min; Superose 6 Increase 10/300 GL column; 21 °C). **(F)** SDS-PAGE analysis (non-reducing and reducing conditions) of NET1 and NEO1-RGMA used to assemble the ternary NEO1-NET1-RGMA complex for SEC-MALS analysis. Traces corresponding to absorbance at 280 nm, light scattering and molecular masses derived from SEC-MALS are shown in black, blue and red, respectively. Calculated molecular masses based on protein amino acid sequences: NET1_ΔNTR_, 49.2 kDa plus 3 Asn-linked glycans, 5.6 kDa; FN domains 4–6 of NEO1, 39.2 kDa plus 2 Asn-linked glycans, 3.8 kDa; RGMA, 42.2 kDa plus 3 Asn-linked glycans, 5.6 kDa. Thus, calculated mass of the glycosylated NEO1-NET1-RGMA ternary 3:3:3 complex is 437.0 kDa. The ternary complex dissociated on SEC-MALS as suggested by a major peak corresponding to 79.97 kDa. However, an additional peak corresponding to 444.4 kDa, which is consistent with the NET1:NEO1:RGMA 3:3:3 mol:mol:mol complex, was also observed. **(G)** FN domains 4–6 of NEO1 co-elute with the full-length extracellular domain of RGMC (RGMC_ECD_) on SEC, suggesting that NEO1 and RGMC form a binary complex. SEC fractions were analyzed using SDS-PAGE under non-reducing and reducing conditions. Under reducing conditions, a fraction of RGMC_ECD_ dissociates into two fragments (labelled N-term. and C-term.) as observed for RGMA_ECD_ (E). **(H**) SEC and SDS-PAGE analysis of the ternary NET1–NEO1–RGMC complex. The ternary NEO1-NET1-RGMC complex elutes as two major peaks (12.5 and 13.9 ml peaks) at lower elution volume compared to the binary NEO1-RGMC complex (16.3 ml, **G**) or NET1 in isolation, suggesting that the NEO1-NET1-RGMC ternary complex forms in solution. SEC running buffer: 150 mM NaCl, 10 mM HEPES pH 7.5, 2 mM CaCl_2_, 1 mM sucrose octasulfate, 0.02% NaN_3_ (flow rate 0.3 ml/min; Superose 6 Increase 10/300 GL column; 21 °C). SEC input was 0.6 ml of the ternary complex at 2.6 mg/ml.

We further interrogated stoichiometry and architecture of the ternary complex using size-exclusion chromatography coupled to multiangle light scattering (SEC-MALS), and cryo-electron microscopy (cryo-EM). The ternary complex composed of NEO1_FN456_, NET1_ΔNTR_ and RGMB_ECD_ could be readily formed on SEC (Figures S2A–S2D). The peak fraction contained all three proteins and was further analyzed using SEC-MALS. The experimentally determined molecular weight (MW) (422.7 ± 2.8 kDa) matched the calculated MW of the glycosylated complex (433 kDa) confirming the presence of the 3:3:3 assembly in solution (Figure S2B). We further analyzed the same sample with cryo-EM (Methods S2). Single particle analysis of the NET1-NEO1-RGMB complex revealed their trimeric symmetry (Figure 2D), consistent with the complex determined by crystallography (Figure 2A). We reconstructed a cryo-EM map to 6.0 Å resolution in which the crystallographic 3:3:3 complex could be readily fitted (Figures 2E and 2F). Using a similar SEC-MALS experiment as for RGMB, we could show that the full-length extracellular domains of the other two human RGM family members RGMA and RGMC form ternary complexes with NEO1_FN456_ and NET1 (Figures S2E–S2H) suggestive of ternary 3:3:3 RGM-NEO1-NET1 architecture.

**Figure 3:**
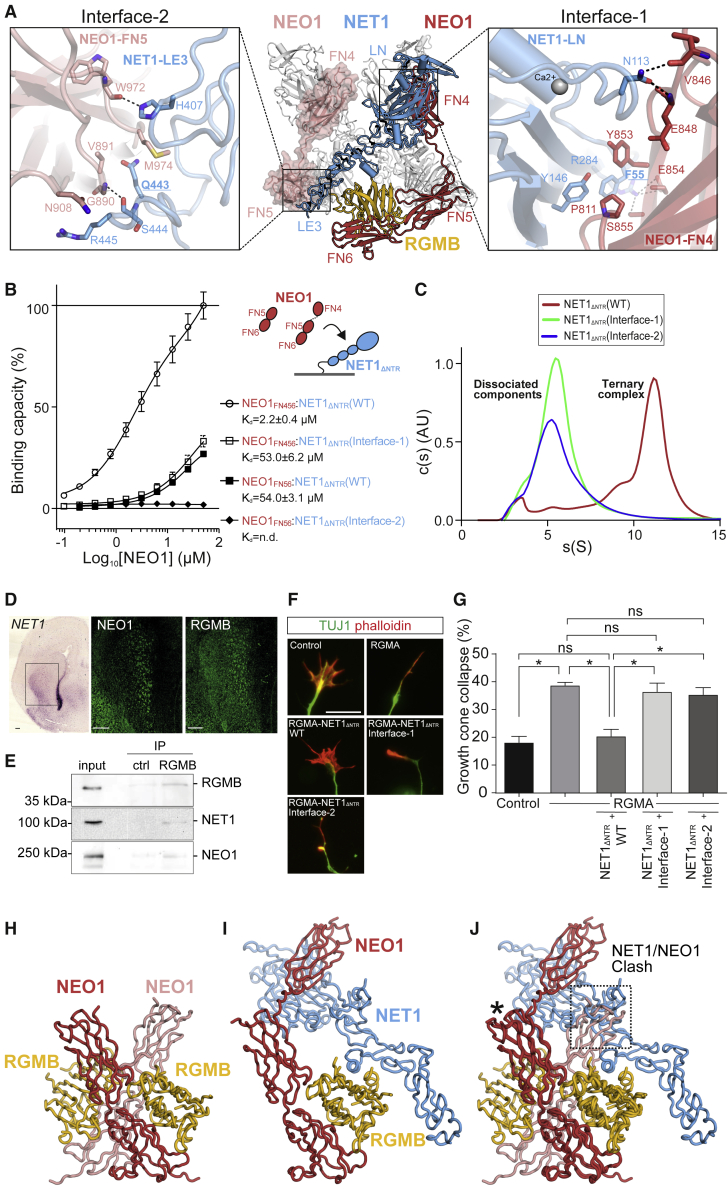
Interface analysis of the ternary NEO1-NET1-RGMB super-complex (A) Close-up views of the observed NET1-NEO1 interfaces (right: interface 1, left: interface 2). Residues are displayed in stick representation and labelled according to domain color-coding. A Ca^2+^ ion bound to NET1 LN (grey sphere) and hydrogen bonds (dashed black lines) are displayed. Mutated residues are in bold and underlined. (B) SPR equilibrium binding curves for the NET1-NEO1 interaction. A schematic of the experiment and the calculated *K*_d_ values are shown. (C) AUC analysis of the NEO1_FN456_-NET1_ΔNTR_-RGMB_ECD_ complex, using NET1_ΔNTR_ WT and mutants. Both NET1 interface-1 and -2 mutants abolish the 3:3:3 stoichiometry of the NEO1-NET1-RGMB super-complex. (D) Overlapping expression of NET1 RNA (in situ hybridization), and NEO1 and RGMB protein (immunohistochemistry) in consecutive coronal sections of E16 mouse striatum. Boxed area is shown at higher magnification for NEO1 and RGMB. Scale bar, 100 μm. (E) RGMB immunoprecipitation (IP) from adult mouse cortex was followed by immunoblotting. Input samples (lane 1), IP using control non-specific IgGs (cntrl) (lane 2), and anti-RGMB IP (lane 3). NEO1 and NET1 co-IP with RGMB from adult mouse brain lysates. (F and G) Functional analysis of the effect of NET1 on RGMA-mediated growth cone collapse. (F) Representative examples of growth cones from mouse P0 cortical neurons. Neurons were stained with the microtubule marker Tuj1 (green) and F-actin marker phalloidin (red). Scale bar, 10 μm. (G) Quantification of growth cone collapse. Growth cones were treated with control or RGMA alone and in combination with different NET1 variants. Proportions of collapsed growth cones relative to control are displayed. n = 3 experiments, one-way ANOVA followed by Tukey’s multiple comparison test. ^∗^p < 0.05. Data are shown as means ± SEM. (H–J) Comparison of binary NEO1-RGM (PDB ID 4BQ6 [Bell et al., 2013]) and the ternary NEO1-NET1-RGMB complexes shown as ribbons. The ternary NEO1-NET1-RGMB protomer complex architecture (I) clashes with the NEO1-RGM dimer-of-dimers signaling conformation (H) when superimposed on NEO1 (marked with an asterisk) (J). See also Figure S3, Figure S4, Figure S5.

**Figure S3:**
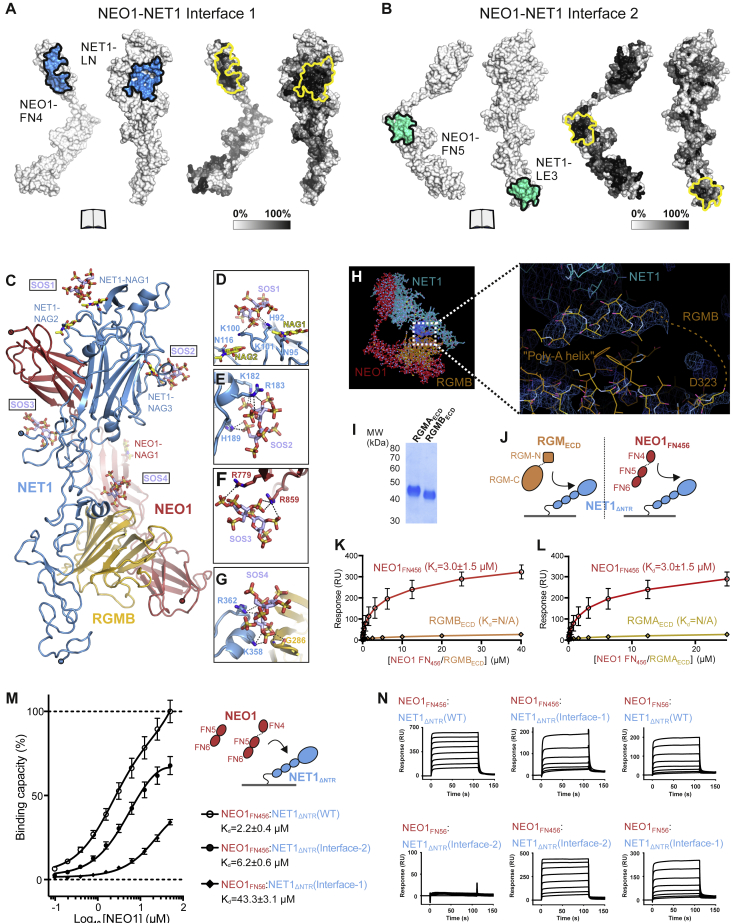
Structural and functional analysis of the ternary NEO1-NET1-RGM complex, related to Figures 2, 3, and 4 **(A, B)** Surface representations of NET1-NEO1 interactions The NEO1-NET1 Interface-1, formed by the NEO1 FN4-NET1 LN interaction is shown in A. Interface residues are mapped onto solvent accessible surfaces displayed in open-book view (blue, left panel in A). Residue conservation calculated with ConSurf server (https://consurf.tau.ac.il/) is mapped onto the protein surfaces according to a white-to-black gradient (right panel in A). Surfaces are highlighted with a line. The NEO1-NET1 Interface-2, formed by the NEO1 FN5-NET1 LE3 interaction is shown in B. Presentation is as in A. **(C-G)** Sugar sites identified on the ternary NEO1-NET1-RGMB crystal structure. (C) Ribbon presentation of the NEO1-NET1-RGMB protomer with the 4 N-linked N-acetylglucosamine (NAG; yellow) and 4 sucrose-octasulfate (SOS; light blue) molecules depicted as sticks. (D-G), Close-up views of the 4 SOS-binding sites with residue side chains within hydrogen-bonding distance shown in stick representation and labelled. Potential hydrogen bonds are displayed as dashed black lines. **(H)** NET1-RGM interaction analysis in the ternary trimer-of-trimers complex determined by X-ray crystallography. Overall 1:1:1 trimer architecture is displayed on the left. The close-up shows the interface between NET1 and RGMB. The sigmaA-weighted 2F_o_-F_c_ map of the final refinement in AUTOBUSTER is displayed and contoured at 1σ. RGMB is ordered to residue D323 and a dashed line denotes disordered residues linking to a putative helical stretch of Ala residues, which were built into this density as the sequence could not be unambiguously assigned. **(I)** Non-reducing SDS-PAGE of purified RGMA_ECD_ and RGMB_ECD_ used as analytes for SPR injections. **(J)** Schematics of the experimental SPR set up. NET1_ΔNTR_ (ligand) was attached to a streptavidin-coupled sensor chip via a biotinylated C-terminal Avi-tag. RGM_ECD_ and NEO1_FN456_ (analytes) were injected to probe interactions. **(K, L),** SPR equilibrium binding curves for NET1_ΔNTR_ binding experiments with NEO1_FN456_ (K and L; same measurement for comparison), RGMB_ECD_ (K) and RGMA_ECD_ (L). **(M, N)** SPR equilibrium binding curves for the NEO1-NET1 interaction. A schematic of the experiment (NEO1: red, NET1: blue) and the calculated K_d_ values are shown. The maximal response for the wild type NEO1_FN456_:NET1_ΔNTR_ interaction represents 100% binding. Sensorgrams for NEO1:NET1_ΔNTR_ interactions, corresponding to Figure 3B and Figure S6J are shown in (B).

The backbone of the “trimer-of-trimers” super-complex is essentially formed by interactions between NEO1 and NET1. NET1 is an elongated and rigid molecule, making contacts with two neighboring NEO1 molecules in order to bridge individual NEO1-NET1-RGM protomers within the super-complex (Figures 2A and 3A and Figures S3A and S3B). The NET1 LN domain (NET1_LN_) forms the major interaction site with NEO1, binding to NEO1_FN4_ (“Interface-1,” Figure 3A, right panel). The combined buried surface area between NET1_LN_ and NEO1_FN4_ is 686 Å^2^ and involves predominantly hydrophobic interactions, with NET1 residue F55 in the center. The second interface is formed by the NET1_LE3_ of the same NET1 molecule and a neighboring NEO1_FN5_ domain. In contrast to “Interface-1,” this interaction is of a predominantly hydrophilic nature with a buried surface area of 572 Å^2^ (“Interface-2,” Figure 3A, left panel). Both interfaces are highly conserved amongst NEO1 orthologues (Figures S3A and S3B). The NEO1 FN5 and FN6 domains interact with RGMB, as observed in our previous structural analysis of binary NEO1-RGM complexes, via the high-affinity “site 1” interface (Bell et al., 2013). A total of 4 N-linked sugars and 4 non-covalently linked sucrose-octasulfate (SOS) molecules are bound at the complex surface (Figures S3C–S3G). The NET1_ΔNTR_ and RGMB_CORE_ molecules form a very minor interaction (Figure S3H), but no binding was observed between NET1_ΔNTR_ and the ectodomains of RGMA and RGMB in solution (Figures S3I–S3L). This suggests that NET1-NEO1 interactions are the driving force for the formation of the “trimer-of-trimers” super-complex.

### The ternary NEO1-NET1-RGMA complex is essential for NET1 inhibition of RGMA-mediated growth cone collapse

In order to verify our observed NET1-NEO1 interfaces, we designed NET1 mutants to ablate specific interaction sites and tested these for NEO1 binding in SPR (Figure 3B, Figures S3M, and 3N). When compared to wild-type NET1_ΔNTR_, NET1_ΔNTR_^F55R^ (“NET1-Interface-1” mutant) designed to disrupt NET1-NEO1 “Interface-1” (Figure 3A, right panel), bound to NEO1_FN456_ with an approximately ten-fold lower affinity but did not change NET1 binding to NEO1_FN56_. On the other hand, NET1_ΔNTR_^Q443N/R445T^ (“NET1-Interface-2”), designed to introduce an Asn443-linked glycan to disrupt the NET1_LE3_-NEO1_FN5_ interface, abolished binding to NEO1_FN56_ (Figure 3B) and reduced NEO1_FN456_ binding approximately two-fold (Figures S3M and S3N). This supports the model of two independent NET1-NEO1 binding interfaces, as observed in our complex structure. We also tested the effects of the NET1_ΔNTR_ Interface-1 and -2 mutants using AUC (Figure 3C). Both NET1 mutant complexes with NEO1_FN456_ and RGMB_ECD_ abolished the “trimer-of-trimers” super-complex, in agreement with our observation that both NET1-NEO1 interface 1 and 2 are necessary to form the super-complex.

**Figure S4:**
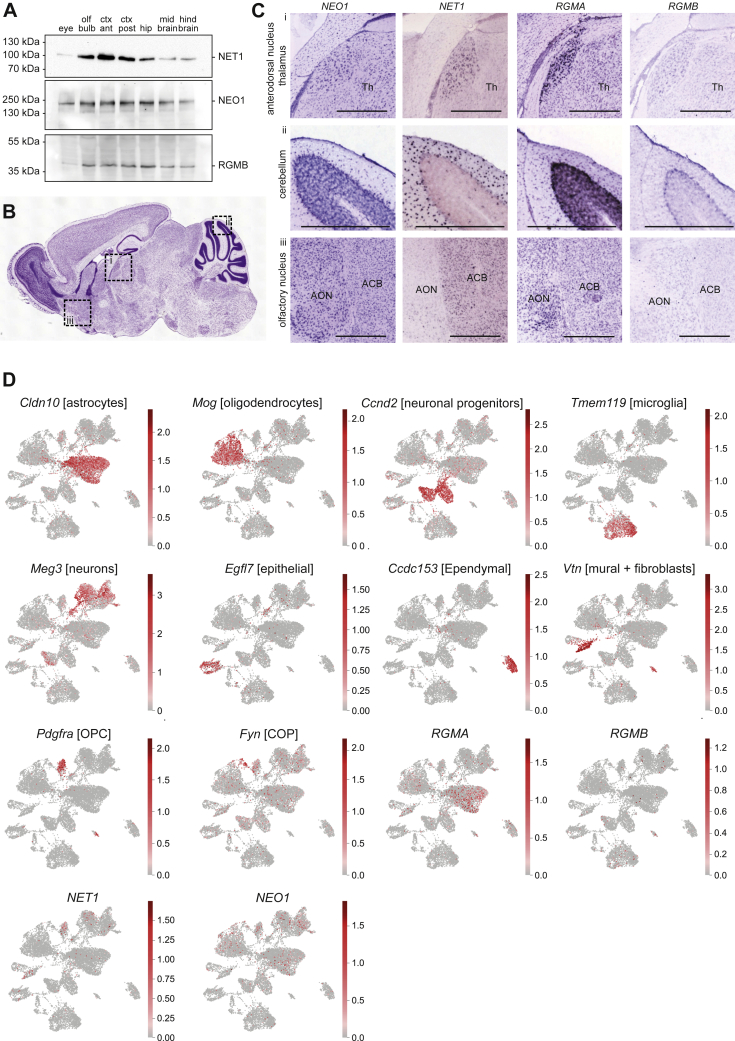
Expression of NEO1, NET1, RGMA and RGMB, related to Figure 3 **(A)** Protein expression of NET1, NEO1 and RGMB in the adult mouse brain detected by western blot analysis. **(B)** Sagittal overview of the adult mouse brain. **(C)***In situ* hybridization for *NEO1, NET1, RGMA* and *RGMB* in sagittal sections from the adult mouse brain (obtained from the Allen Brain Atlas (brain-map.org)). Regions of interest are indicated in boxed regions in B: (i) anterodorsal nucleus of the thalamus, (ii) cerebellum and (iii) olfactory nucleus. Images are obtained from the Allen Brain Atlas. Olf bulb, olfactory bulb; ctx ant, anterior half of the cortex; ctx post, posterior half of the cortex; hip, hippocampus; Th, thalamus; AON, anterior olfactory nucleus; ACB, nucleus accumbens. Scale bar = 500 μm. **(D)** scRNAseq dataset analysis (Mizrak et al 2019) for co-expression of *RGMA/B, NEO1* and *NET1* in adult V-SVZ. Single-cell expression levels of cluster-specific marker genes in adult ventricular-subventricular zone (V-SVZ) cells plotted on UMAP embedding (*Cldn10, Mog, Ccnd2, Tmem119, Meg3, Egfl7, Ccdc153, Vtn, Pdgfra, Fyn*). In addition, expression levels of *Neogenin (NEO1)*, *Netrin-1 (NET1)*, *RGMA* and *RGMB* are shown. Clusters marked as [clusterID]. OPC, oligodendrocyte precursor; COP, committed oligodendrocyte precursors.

To assess the functional impact of NET1 on the NEO1-RGM interaction, we confirmed that RGMA, RGMB, NET1, and NEO1 expression patterns in the nervous system allow interactions between these molecules in a “trimer-of-trimers” super-complex. We found widespread overlap in the distribution and expression of RGMs, NET1, and NEO1 in various brain regions of the adult mouse brain, including in the thalamus, cortex, and cerebellum (Figures S4A–S4C). For example, in line with previous data, these molecules were localized to cell types in the ventricular-subventricular zone, such as astrocytes and neurons, that reside in close proximity and that functionally interact (Figure S4D). Furthermore, RGMB, NET1, and NEO1 also displayed overlapping expression in different regions of the embryonic brain, including the striatum (Figure 3D). Next, we assessed whether RGMs, NET1, and NEO1 are present in the same protein complex in brain tissue. We performed immunoprecipitation (IP) of RGMB from adult mouse cortex followed by detection of RGMB, NET1, and NEO1. As predicted by the PLA experiments (Figures 1B and 1C) and expression data (Figures 3D and S4A), NEO1 and NET1 were co-immunoprecipitated with RGMB (Figure 3E). These observations, together with our data showing lack of RGMB-NET1 and RGMB-DCC binding (Figures S3K and S3L) (Bell et al., 2013), suggest that RGMs, NET1, and NEO1 co-exist in multimeric protein complexes on neural cells.

**Figure S5:**
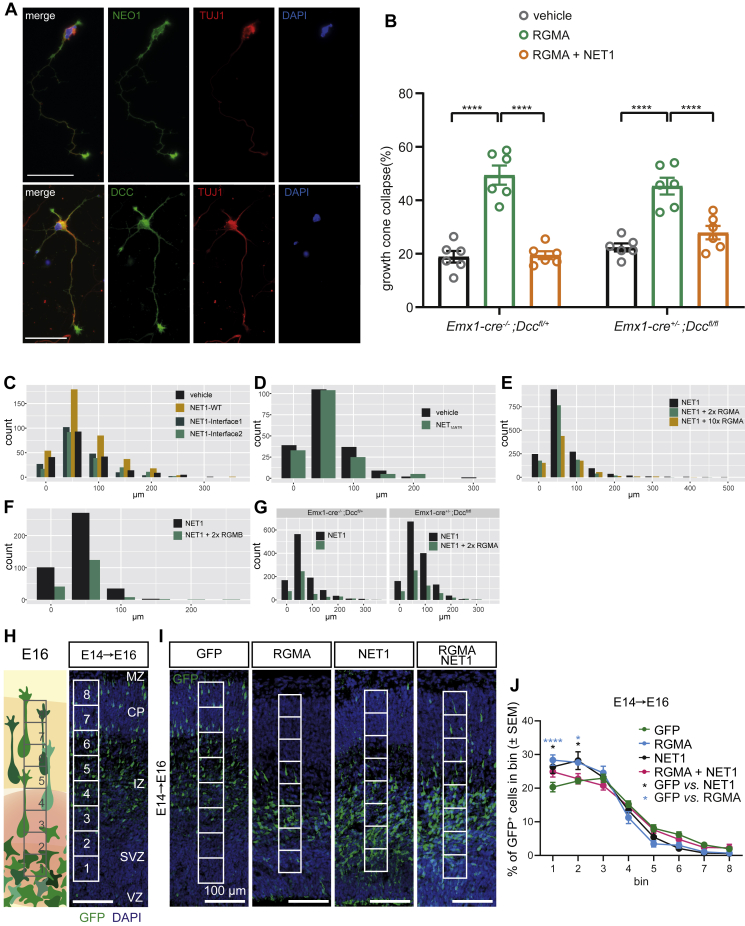
Silencing of RGMA-mediated growth cone collapse by NET1 is DCC-independent, related to Figures 3, 5, 6, and 7 **(A)** Immunocytochemistry of NEO1, deleted in colorectal cancer (DCC) and TUJ1 in P0 mouse cortical neurons at DIV3. Scale bar = 50 μm. **(B)** Mean ± S.E.M. of the percentage of collapsed growth cones following exposure to RGMA or RGMA + NET1_FL_ in cortical neurons from *Emx1-Cre*^*-/-*^*;Dcc*^*fl/+*^ (control) or *Emx1-Cre*^*+/-*^*;Dcc*^*fl/fl*^ (knockout) mice. *Emx1-Cre*^*-/-*^*;Dcc*^*fl/+*^ (mean ± S.E.M.): vehicle = 18.83 ± 2.17, RGMA = 49.40 ± 3.61, RGMA + NET1_FL_ = 19.47 ± 1.47. *Emx1-Cre*^*+/-*^*;Dcc*^*fl/fl*^ (mean ± S.E.M.): vehicle = 22.35 ± 1.48, RGMA = 45.28 ± 3.15, RGMA + NET1_FL_ = 27.88 ± 2.53. n = 6 experiments, two-way ANOVA with Tukey’s multiple comparisons test, ^∗∗∗^ p < 0.0001. **(C-G)** Quantification of migration distance in SVZ-NSC assays and analysis of GFP^+^ neurons following IUE. Migration distance (per 50 μm bin) of SVZ-neuroblasts related to (C) Figures 5C and 5D, (D) Figures 5E and 5F, (E) Figures 6A and 6B, (F) Figures 6I and 6J, and (G) Figures 6G and 6H. **(H-J)** Cortical migration of GFP^+^ electroporated neurons. (H) At E14 embryos were *in utero* electroporated (IUE) with expression vectors for GFP, RGMA, and/or NET1 (each condition has GFP). Embryos were harvested two days later at E16. Migration distance from the VZ to the MZ was measured per bin (1-8) (i.e. the number of GFP^+^ cells per bin/total GFP^+^ cells). (I-J) Electroporation of RGMA or NET1 caused an increase in the number of GFP^+^ in bins near the VZ, indicating reduced migration towards the MZ. Simultaneous overexpression of RGMA and NET1 in part rescued this inhibitory effect. The reduction in the number of GFP^+^ cells in more upper layers was visible in the images but did not reach statistical significance due to the low numbers of these more superficially located neurons. One-way ANOVA followed by Sidaks multiple comparisons test: RGMA vs. GFP bin 1 p < 0.0001, RGMA vs. GFP bin 2 p = 0.0305, NET1 vs. GFP bin 1 p = 0.379, NET1 vs. GFP bin 2 p = 0.362. GFP: n = 6 animals, RGMA: n = 6 animals, NET1: n = 4 animals, NET1+RGMA: n = 5 animals. Marker: 100 μm. VZ, ventricular zone; SVZ, subventricular zone; IZ, intermediate zone; CP, cortical plate; MZ, marginal zone.

To investigate the functional role of these protein complexes, we first used an NEO1-dependent growth collapse assay in which dissociated cortical neurons (CNs) were exposed to RGMA in the absence or presence of NET1 proteins (van Erp et al., 2015) (Figures 3F and 3G). CNs express NEO1 (Figure S5A), and addition of RGMA induced CN growth cone collapse (van Erp et al., 2015). This inhibitory effect of RGMA was diminished when CNs were simultaneously exposed to RGMA and wild type NET1_ΔNTR_, suggesting that NET1 can inhibit RGMA-mediated growth cone collapse. In contrast, when CNs were incubated with RGMA and either NET1 interface-1 or interface-2 mutant, RGMA-mediated growth cone collapse was unaffected (Figures 3F and 3G). Since embryonic CNs express NEO1 and DCC (Figure S5A), we confirmed that the observed effect of NET1 was independent of the NET1 receptor DCC (that shares some 80% sequence similarity to NEO1). We crossed *Emx1-IRES-cre* mice with *Dcc*^*fl/fl*^ mice (Krimpenfort et al., 2012), to delete *Dcc* from CNs (Gorski et al., 2002; Liang et al., 2012). We observed no difference in RGMA-induced growth cone collapse and NET1 rescue in *Dcc*^lox/lox^;*Emx1*^cre/wt^ compared to *Dcc*^lox/wt^;*Emx1*^wt/wt^ CNs (Figure S5B). Taken together, our functional and structural analyses suggest a mechanism for NET1-mediated inhibition of NEO1-RGM signaling, whereby the formation of the NEO1-NET1-RGM “trimer-of-trimers” super-complex is the driving force as it is incompatible with NEO1 dimerization and subsequent downstream signal activation (Figure 3H–3J).

### The structure of the binary NET1-NEO1 complex suggests NET1-mediated NEO1 clustering

**Figure 4:**
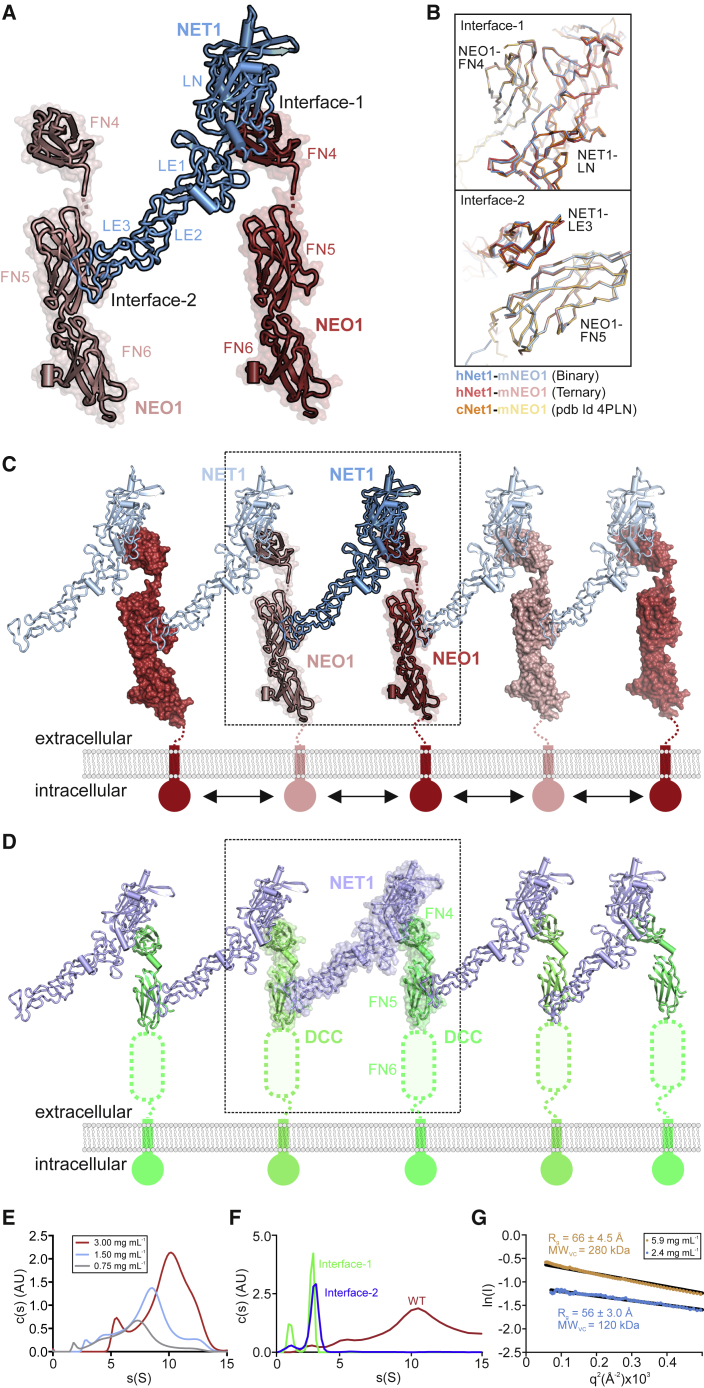
Structure and functional characterization of the binary NET1-NEO1 complex (A) Cartoon representation of the binary NET1_ΔNTR_-NEO1_FN456_ complex. NET1_ΔNTR_ contacts two NEO1_FN456_ chains, using the same interfaces observed in the “trimer-of-trimers” NEO1-NET1-RGMB super-complex structure. Interfaces 1 and 2 are labelled. (B) Comparison of NET1-NEO1 interfaces (interface 1: top panel, interface 2: lower panel). Superpositions were calculated using NEO1 FN4 (top panel, “interface-1”) and FN5 (lower panel, “interface-2”) as template. The binary (light blue/blue) and ternary (light red/red) NET1_ΔNTR_-NEO1_FN456_ complexes from this study and the previously determined NET1_ΔNTR_-NEO1_FN45_ complex (orange/light orange, PDB Id. 4PLN [Xu et al., 2014]) are shown as ribbons. (C) Overall arrangement of the NET1-NEO1 complex, which forms a continuous array in the crystal. The relative orientation of the plasma membrane is depicted. The region marked corresponds to the protomer in (A). (D) Cartoon representation of the previously published DCC_FN45_-NET1_ΔNTR_ complex (PDB Id 4PLO [Xu et al., 2014]) shown in the same orientation as the NET1_ΔNTR_-NEO1_FN456_ complex from C. Both complexes form a similar, continuous array in the crystal. The DCC FN6 domain missing in the DCC-NET1 complex is depicted schematically. (E and F) Sedimentation velocity AUC experiments of the binary NET1_ΔNTR_-NEO1_FN456_ complex. The complex reveals concentration-dependent increase of oligomerization, characterized by a shift to higher s(S) values (E). This can be inhibited by NET1 interface-1 and -2 mutants that both result in a reduction of the apparent molecular weight (F), corresponding to a 1:1 NET1-NEO1 complex stoichiometry. (G) Guinier region analysis of SEC-SAXS data collected for the NEO1_FN456_:NET1_ΔNTR_ complex at 2.4 mg mL^−1^ (blue) and 5.9 mg mL^−1^ (yellow) gives larger R_g_ and MW_VC_ values at higher concentrations. See also Figure S6.

**Figure S6:**
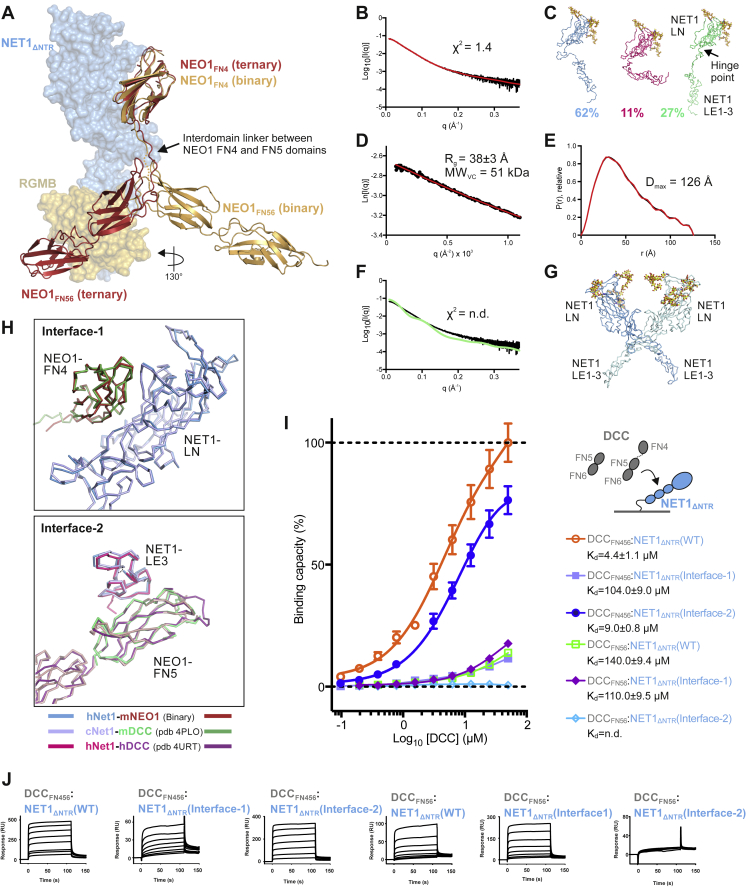
Structural and functional analysis of binary NET1-NEO1 and NET1-DCC complexes, related to Figure 4 **(A)** Flexibility between NEO1 FN4 and FN5-6 domains. Superposition of the binary NEO1-NET1 (gold) and NEO1-NET1-RGMB (red) complex structures. Superimpositions were calculated using NET1 as template. NET1 and RGMB are colored as in Figure 1A. Due to flexibility in the interdomain linker region between FN domains 4 and 5, the position of the NEO1 FN5-6 region varies greatly in relation to the FN4 domain. NEO1_FN56_ forms a structural unit. (B, C) Fit of an ensemble of NET1_ΔNTR_ models to experimental scattering data. Experimental (black) and calculated (red) scattering curves are displayed to a maximal momentum transfer of q = 0.37 Å^-1^, with fit value (χ^2^) displayed (B). A distribution of NET1_ΔNTR_ models as calculated by MultiFOXS and MES is displayed, color-coded as per model (C) **(D)** Guinier region for experimental and calculated scattering, with radius of gyration (R_g_) calculated from experimental data annotated. **(E)** Normalized pair-distance distribution function, with the derived maximum intra-particle diameter (D_max_). This suggests that NET1_ΔNTR_ behaves as a monomer in solution. **(F-G)** Fitting between experimental (black) and calculated (green) scattering data (F) from a proposed X-linked NET1_ΔNTR_ dimer (G) (PDB ID. 4PLN). **(H)** Comparison of the binary NEO1-NET1 and DCC-NET1 interfaces (‘Interface-1’: left panel, ‘Interface-2’: right panel). Superpositions were calculated using NEO1 FN4 (for ‘Interface-1’) and FN5 (for ‘Interface-2’) as template, respectively. The binary NEO1_FN456_-NET1_ΔNTR_ complex from this study and the previously determined DCC_FN45_-NET1_ΔNTR_ (PDB ID 4PLO) and DCC_FN56_-NET1_ΔNTR_ (PDB ID 4URT) complexes are shown as ribbons. **(I, J)** SPR binding analysis to characterize the NET1 interaction with the NEO1 paralogue DCC. SPR equilibrium binding curves for the DCC-NET1 interaction (B) and corresponding sensorgrams (**C**) are presented. A schematic of the experiment (DCC: grey, NET1: blue) and the calculated Kd values are shown. The maximal response for the wild type DCC_FN456_:NET1_ΔNTR_ interaction represents 100% binding.

We next determined the crystal structure of the binary complex between NEO1_FN456_ and NET1_ΔNTR_ (Figure 4A; Table S1). The observed NEO1_FN456_:NET1_ΔNTR_ interfaces are equivalent to the ternary NEO1-NET1-RGMB complex, with NET1 linking two separate NEO1 molecules via their FN4 and FN5 domains respectively (Figure 4A). The NEO1_FN56_ structural unit is positioned differently when compared to the ternary NEO1-NET1-RGM structure, undergoing a rotation of some 130^o^ around the FN4-5 interdomain linker (Figure S6A). Interface-1 and -2 determinants resemble that of our ternary complex and are also observed in a previously reported crystal structure of the chick NET1_ΔNTR_ with NEO1_FN45_ (Xu et al., 2014) (Figure 4B). However, we did not observe a 2:2 hetero-tetrameric arrangement as in the chick NET1-NEO1 structure, mediated by an antiparallel NET1 dimer. NET1_ΔNTR_ exists as a monomer at concentrations up to 81 μM when analyzed by SAXS (Figures S6B–S6G), in agreement with previous NET analyses (Finci et al., 2014; Grandin et al., 2016; Reuten et al., 2016). Crystal packing analysis of our binary NET1-NEO1 complex suggests a continuous NET1-NEO1 arrangement generated by a crystallographic two-fold axis, mediated by NET1-NEO1 interface 1 and 2 (Fig. 4C). This suggests a ligand-induced receptor clustering mechanism, resulting in a similar arrangement to that proposed for the NET1-DCC complex (Xu et al., 2014) (Figure 4D). In both complexes, interfaces 1 and 2 are highly conserved (Figure S6H). Our SPR analysis using NET1 interface-1 and-2 mutants is in agreement with this model, with equivalent NET1-binding properties observed for DCC (Figures S6I and S6J) as for NEO1 (Figure 3B).

To validate the NET1-NEO1 clustering model, we performed AUC analysis of the NEO1_FN456_-NET1_ΔNTR_ complex. We observed a concentration-dependent shift of the complex towards higher molecular weights (10–15 S), indicative of NET1_ΔNTR_-induced NEO1 clustering (Figure 4E). This was inhibited by mutations in both NET1-NEO1 interface 1 and 2 (Figure 4F). In support of this finding, analysis of the NEO1_FN456_-NET1_ΔNTR_ complex using SAXS gave a notable difference in scattering profile and calculated molecular weight at different concentrations (Figure 4G). Our data support a cell surface receptor oligomerization model in which a single NET1 molecule links two NEO1/DCC receptors together, leading to NEO1/DCC clustering in a concentration-dependent manner.

### RGM inhibition of NET1-mediated neuronal migration

**Figure 5:**
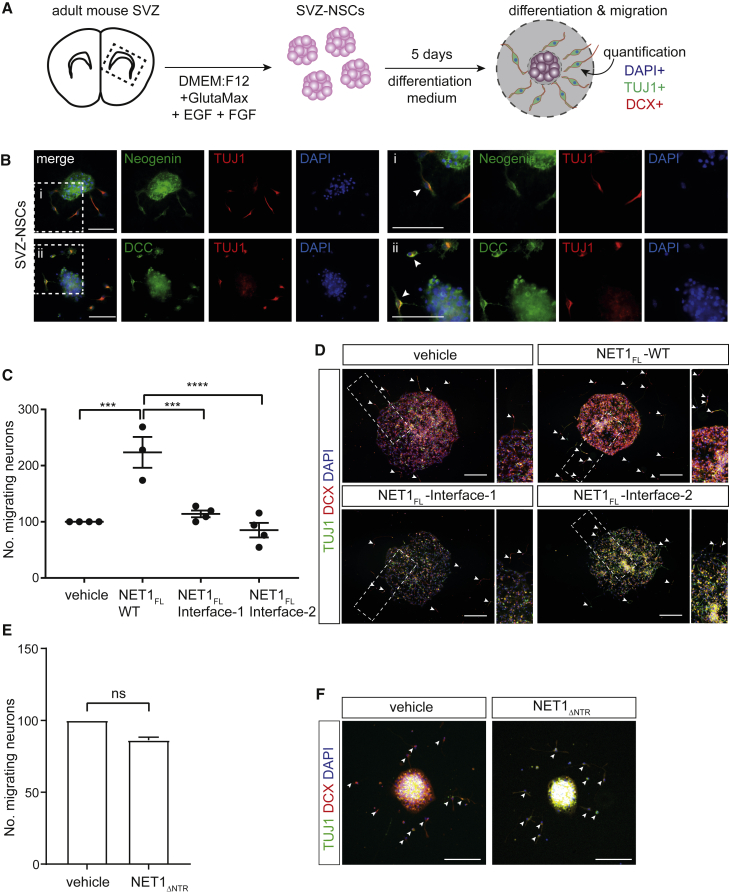
NET1 mediates SVZ-neuroblast migration via NEO1 (A) Schematic of the neurosphere migration assay. Neurospheres were generated from the adult mouse subventricular zone (SVZ) subsequently plated on control or NET1 proteins. (B) Immunocytochemisty for NEO1, DCC, and TUJ1 (to label SVZ-neuroblasts) in DIV5 SVZ-NSC cultures. SVZ-neurospheres (NSCs) and neuroblasts (arrowheads) express NEO1 and DCC. Boxed areas are shown at higher magnification on the right. Scale bar, 50 μm. (C and D) Analysis of migrating neurons from SVZ-NSCs grown on full-length NET1 constructs. Ablation of either NET1-NEO1 interface-1 or -2 interactions causes loss of NET1-mediated neuron migration. Mean ± SEM of the relative number of Tuj1/DCX positive migrating neurons per neurosphere: vehicle = 100.00, NET1_FL_-WT = 223.25 ± 27.51, NET1_FL_-Interface-1 = 114.20 ± 6.07, NET1_FL_-Interface-2 = 85.06 ± 13.02, n = 3-4 experiments. Brown-Forsythe to test significant difference between SDs (p < 0.05): ns. One-way ANOVA followed by Tukey’s multiple comparisons test: vehicle vs. NET1_FL_-WT P = 0.0003, NET1_FL_-WT vs. NET1_FL_-Interface-1 P = 0.0007, NET1_FL_ vs. NET1_FL_-Interface-2 p < 0.0001. Representative samples of mouse SVZ-NSCs grown on coverslips coated with indicated proteins are shown in (D). Boxed areas are shown at higher magnification on the right of each panel. Migrating neurons (white arrowheads) were identified via labelling with the microtubule markers TUJ1 (green) and DCX (red) as well as the nuclear marker DAPI (blue). (E and F) Analysis and representative samples of migrating SVZ neuroblasts (white arrowheads) grown on NET1_ΔNTR_. NET1 lacking the C-terminal NTR domain fails to increase neuron migration. Mean ± S.E.M of the relative number of TUJ1/DCX-positive migrating neurons per neurosphere: control (vehicle) = 100.00, NET1_ΔNTR_ = 86.18 ± 2.215, n = 2 individual experiments. Unpaired t test: p = 0.0247. See also Figure S5.

RGMA is a repulsive guidance cue for cortical interneurons migrating out of the MGE and it induces growth cone collapse in CNs. In line with our discovery of the “trimer-of-trimers” silencing complex, these effects can be silenced by NET1 (O’Leary et al., 2013) (Figures 3F and 3G). To functionally examine the NET1-NEO1 interaction and to test whether RGMs can inhibit NET1-mediated NEO1 signaling, we cultured mouse subventricular zone neurospheres (SVZ-NSCs) on different NET1 variants. NET1 and RGMA are expressed along the migratory route of mouse SVZ-neuroblasts *en route* to the olfactory bulb in the rostral migratory stream (RMS) and NET1 promotes SVZ-neuroblast migration *in vitro* (Bradford et al., 2010; O’Leary et al., 2015). This migration assay allows testing of NET1-NEO1 signaling output, because SVZ-neuroblasts express NEO1 and rely on this receptor for NET1-mediated migration (Figures 5A and 5B) (O’Leary et al., 2015).

SVZ-NSCs grown on full-length NET1 (NET1_FL_) showed a significant increase in the number of migrating neurons compared to control substrate (Figures 5C and 5D), in line with previous work (O’Leary et al., 2015). Interestingly, NET1_ΔNTR_ (that inhibits RGM-mediated growth cone collapse [Figure 3G]) was not sufficient to activate NEO1-depenent cell migration (Figures 5E and 5F), suggesting a crucial role of the NET1 C-terminal NTR domain, potentially because it interacts with heparan sulphate proteoglycans (Kappler et al., 2000). When NET1_FL_ interface-1 or -2 mutants were used no enhanced migration was observed, in agreement with our structural and biophysical analyses and supporting that both NET1-NEO1 interfaces are essential for function (Figures 5C and 5D).

**Figure 6:**
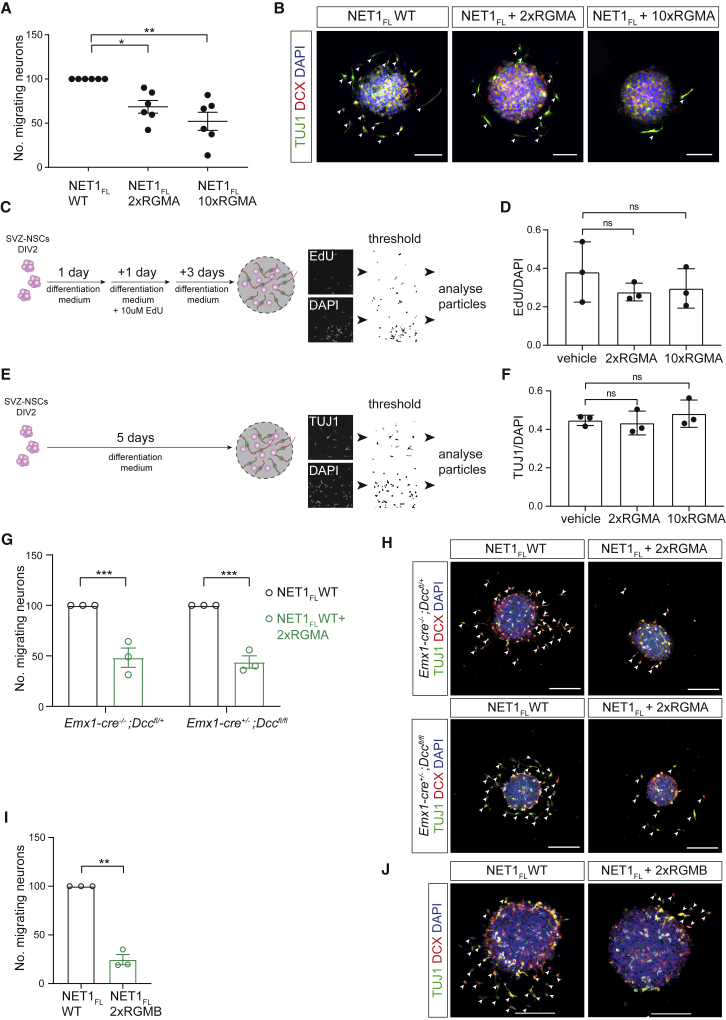
RGMs inhibit NET1-mediated SVZ-neuroblast migration (A and B) RGMA inhibits SVZ-neuroblast migration mediated by NET1-NEO1 signaling in a concentration-dependent manner. Analysis (A) and representative samples (B) of SVZ-NSCs grown on full-length NET1 and different concentrations of mouse RGMA. 2x RGMA = 1.2 μg/ml, 10x RGMA = 6.0 μg/ml. Mean ± SEM of the relative number of TUJ1/DCX-positive migrating neurons per neurosphere: NET1_FL_-WT = 100.00, NET1_FL_-WT + 2x RGMA = 68.52 ± 7.17, NET1_FL_-WT + 10x RGMA = 52.04 ± 10.27, n = 6 experiments. Bartlett’s test to test significant difference between SDs (p < 0.05): p <0.0001. Kruskal-Wallis followed by Dunn’s multiple comparisons test: NET1_FL_-WT vs. NET1_FL_-WT + 2x RGMA p = 0.0289, NET1_FL_-WT vs. NET1_FL_-WT + 10x RGMA p = 0.0023. Arrowheads indicate neuroblasts. Scale bar, 50 μm. (C**–**F) RGMA does not influence SVZ neurosphere proliferation and differentiation. (C) Overview of the proliferation assay. (D) Ratio of EdU-positive over DAPI-positive cells. Mean ± SD vehicle = 0.381 ± 0.091, 2x RGMA = 0.277 ± 0.027, 10x RGMA = 0.296 ± 0.059. n = 3 experiments, one-way ANOVA with Tukey’s multiple comparisons test, vehicle vs. 2x RGMA p = 0.5238, vehicle vs. 10x RGMA p = 0.6369. (E) Overview of the differentiation assay. (F) Ratio of TUJ1-positive over DAPI-positive cells. Mean ± SD vehicle = 0.447 ± 0.016, 2x RGMA = 0.433 ± 0.036, 10x RGMA = 0.482 ± 0.041. n = 3 experiments, one-way ANOVA with Tukey’s multiple comparisons test, vehicle vs. 2x RGMA p = 0.9389, vehicle vs. 10x RGMa p = 0.6897. (G and H) Silencing of NET1-mediated neuronal migration in neurospheres by RGMA is DCC-independent. Analysis (G) and representative samples (H) of SVZ-NSCs derived from *Emx1*^*wt/wt*^*;Dcc*^*lox/wt*^ (control) and *Emx1*^*cre/wt*^*;Dcc*^*lox/lox*^ (DCC knockout) mice grown on full-length NET1 with and without addition of 2x RGMA. Mean ± SEM of the relative number of TUJ1/DCX-positive migrating neurons per neurosphere: *Emx1*^*wt/wt*^*;Dcc*^*lox/wt*^ mean ± SEM NET1_FL_ = 100.00, NET1_FL_ + 2x RGMA = 48.263 ± 9.535, *Emx1*^*cre/wt*^*;Dcc*^*lox/lox*^; mean ± SEM NET1_FL_ = 100.00, NET1_FL_ + 2x RGMA = 43.977 ± 6.099. n = 3 experiments, two-way ANOVA with Sidak’s multiple comparisons test, NET1_FL_ vs NET1_FL_ + 2x RGMA p < 0.0004 for both genotypes. Arrowheads indicate neuroblasts. Scale bar, 50 μm. (I and J) RGMB inhibits neuroblast migration mediated by NET1. Analysis (I) and representative samples (J) of SVZ-NSC cultures grown on full-length NET1 with and without addition of 2x RGMB. Mean ± SEM of the relative number of TUJ1/DCX-positive migrating neurons per neurosphere: NET1_FL_ = 100.00, NET1_FL_ + 2x RGMB = 24.55 ± 5.253. n = 3 experiments, paired two-tailed t test p = 0.0048. Scale bar, 50 μm. See also Fig. S5.

Next, we examined whether RGMA can counteract the positive effect of NET1 on neuron migration and found that RGMA blocked the promotion of SVZ-neuroblast migration by NET1_FL_ in a concentration-dependent manner (Figures 6A and 6B, Figures S5C–S5G). Addition of RGMA had no effect on neuroblast proliferation or differentiation (Figures 6C–6F). Furthermore, we showed that NET1 could induce neuroblast migration following DCC ablation while RGMA could still inhibit this effect (Figures 6G and 6H, Figures S5C–S5G; data not shown). RGMB also blocked the promotion of SVZ-neuroblast migration by NET1 (Figures 6I and 6J) suggesting a general effect of RGMs.

**Figure 7:**
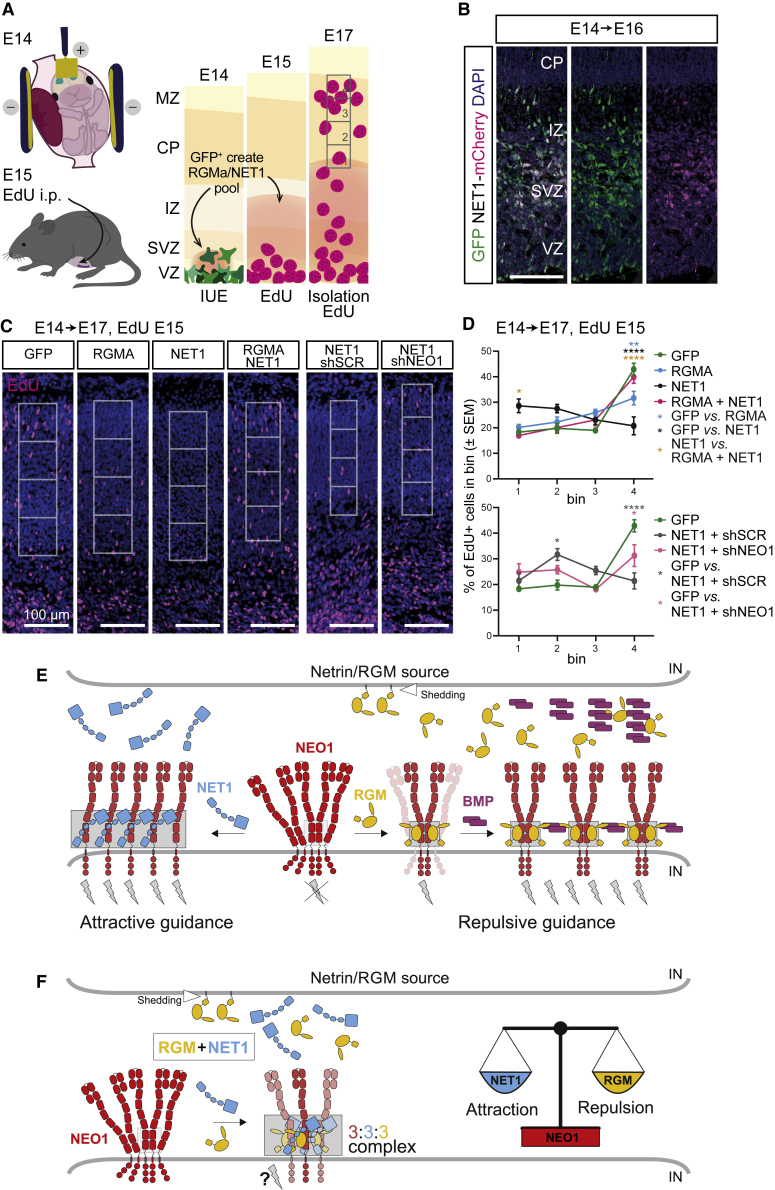
*In vivo* inhibitory interactions between RGMA and NET1 (A**–**D) *In vivo* inhibitory effects of RGMA and NET1 on embryonic mouse cortical neuron migration are silenced in the presence of both cues. (A) Graphical overview of the *in utero* electroporation (IUE) experiment. Embryos were electroporated at E14 with a GFP construct in addition to (combinations of) different expression vectors (RGMA, NET1, or shRNA). At E15, pregnant mothers were injected with EdU to label the population of cortical neurons born at E15. At E17, migration of Edu^+^ neurons was quantified in the cortical plate (CP) in 4 different bins (1–4). (B) Immunohistochemistry showing NET1 expression in the deep part of the E16 cortex following co-electroporation of GFP and NET1-mCherry. (C) EdU staining on E17 coronal sections of the mouse cortex to visualize migrating neurons born at E15, one day after IUE of the VZ at E14. Scale bar, 100 μm. (D) Quantification of Edu^+^ neuron migration using the bins shown in (C). Upper graph, IUE of RGMA and NET1 constructs reduced migration of EdU^+^ neurons, an effect silenced when RGMA and NET1 are co-electroporated. Lower graph, reduced migration of neurons following NET1 electroporation is partly rescued by knockdown of NEO1 (shNEO1). One-way ANOVA followed by Sidak’s multiple comparisons: RGMA vs. GFP bin 4 p = 0.0094, NET1 vs. GFP bin 4 p < 0.0001, NET1 vs. RGMA+NET1 bin 1 p = 0.0231, NET1 vs. RGMA+NET1 bin 4 p < 0.0001, NET1+shSCR vs. GFP bin 2 = 0.0366, NET1-shSCR vs. GFP bin 4 p < 0.0001, NET1+shNEO1 vs. GFP bin 4 p = 0.0108. GFP, RGMA, and NET1+ RGMA: n = 6 embryos, NET1 and NET1+shSCR: n = 4 embryos, NET1+shNEO1: n = 7 embryos. i.p., intraperitoneally; E, embryonic day; VZ, ventricular zone; SVZ, subventricular zone; IZ, intermediate zone; MZ, marginal zone. (E and F) Model for NEO1 signaling via the NET1 and RGM guidance molecules in trans. (E) NET1-induced clustering of NEO1 at the cell surface via Interface-1 and -2 interactions can lead to NEO1 intracellular interactions, inducing e.g. attractive guidance and outgrowth (left panel). In contrast, RGM binding to potentially pre-clustered NEO1 results in NEO1 dimerization in a signaling compatible conformation (Bell et al., 2013) (right panel). This architecture leads to activation of downstream signaling resulting in repulsive guidance (e.g., growth cone collapse), a process that can be potentiated by BMP morphogens (Healey et al., 2015). (F) Combined binding of RGM and NET1 to NEO1 results in “trimer-of-trimers” super-complexes, preventing cell surface clustering, thereby inhibiting both RGM-mediated repulsive but also NET1-mediated attractive signaling. See also Figure S5, Figure S7, Figure S8.

Finally, we assessed whether interactions between RGM and NET1 can lead to silencing of their individual biological effects *in vivo*. In the embryonic cortex, RGMA acts as a repulsive cue for migrating CNs via NEO1 (van Erp et al., 2015). NET1 promotes the migration of various types of neurons and displays very low expression in the cortex (Brignani et al., 2020; O’Leary et al., 2015; Yung et al., 2018). We designed an *in utero* electroporation (IUE) study to examine whether NET1 could silence the repulsive effect of RGMA on cortical neuron migration *in vivo*. Expression constructs for RGMA or NET1 (in combination with a GFP plasmid for visualization) were electroporated at E14 following which the pregnant mothers received a pulse of EdU at E15. EdU labelling enabled analysis of neurons that would migrate into a region of strong RGMA expression (±NET1 expression) that was generated by IUE at E14. Expression vectors were targeted to neuronal progenitors in the VZ at E14, followed by immunostaining at E16 or E17 (Figures 7A and 7B). Three days after GFP electroporation, EdU^+^ neurons were found throughout the cortex, including in the upper CP (Figures 7C and 7D). Electroporation of RGMA created a non-permissive zone for subsequent EdU^+^ neurons, resulting in a reduction of the number of EdU^+^ neurons in upper cortical areas (quantified in the CP) (Figures 7C and 7D). Electroporation of NET1 also reduced the migration of EdU^+^ neurons, most likely because neurons got trapped in deeper regions exogenously expressing this attractive cue (Figures 7C and 7D). Knockdown of NEO1 partially rescued the reduced migration of EdU^+^ neurons, indicating that the NET1-mediated effect requires NEO1 (Figures 7C and 7D). Finally, we tested co-electroporation of RGMA and NET1 and failed to detect a reduction in CN migration, both following analysis of EdU^+^ and GFP^+^ neurons (Figures 7C and 7D, Figures S5H–S5J). These data together with our observations from growth cone collapse and SVZ-NSC experiments (Figures 3, 5, and 6) and work by others (O’Leary et al., 2013) suggest that simultaneous binding of the functionally competing ligands NET1 and RGM blocks NEO1 receptor signaling.

## Discussion

Many guidance receptors bind multiple ligands, but how signaling downstream of these ligand-receptor interactions is integrated remains poorly understood (Dudanova and Klein, 2013; Morales and Kania, 2017). Here, we determine structures of a ternary NEO1-NET1-RGM complex and show that the guidance cues RGM and NET1 can bind simultaneously to NEO1 in a “trimer-of-trimers” super-complex. This ternary complex exists at the cell membrane, and its formation inhibits both RGMA-NEO1-mediated growth cone collapse and RGMA- and NET1-NEO1-mediated cell migration *in vitro* and *in vivo*. The ternary structure acts as a silencing complex, preventing formation of the signaling-compatible RGM-NEO1 complex and NET1-induced NEO1 ectodomain clustering. These results illustrate how simultaneous binding of structurally distinct ligands with specific cellular functions to a single receptor can lead to the formation of a complex that silences downstream signaling.

NEO1 is a multi-domain receptor with various interaction partners and signaling outputs. NEO1 oligomerization is required for signaling, with the 2:2 NEO1:RGM signaling compatible complex triggering growth cone collapse and cell repulsion (Bell et al., 2013) (Figure 7E, middle). This process can be potentiated by BMP morphogens (Tassew et al., 2014), with RGM acting as a physical link bridging BMP and NEO1, inducing NEO1 clustering (Healey et al., 2015) (Figure 7E, right). NET1 binding to NEO1 or the NEO1 orthologue DCC can trigger attractive guidance responses, and NET1-DCC binding induces receptor clustering (Stein et al., 2001; Xu et al., 2014). Here, we show that the binary NET1-NEO1 complex behaves in a similar way (Figure 7E, left), suggesting that NET1-induced chemoattraction is triggered by NEO1 receptor clustering. Despite both ligands binding in the juxtamembrane FN4-6 region, our data show that RGM and NET1 simultaneously target independent conserved binding sites on NEO1 and trigger a unique receptor conformation incompatible with the 2:2 NEO1-RGM and clustered NET1-NEO1 oligomeric states. This creates a “crossroads” between NET1-NEO1 and NEO1-RGM complexes at the cell surface with a NEO1-NET1-RGM “trimer-of-trimers” super-complex fine-tuning the output of this fundamental signaling pathway (Figure 7F). In this context, two functionally competing ligands (NET1 and RGM) act to block NEO1 signaling by forming a silencing complex.

The “primary” receptor for NET1 has long been thought to be DCC. However, recent studies revealed that NET1 can mediate chemoattractive responses through NEO1 (Huyghe et al., 2020; O’Leary et al., 2015; Xu et al., 2014) (Figures 5 and 6). Our binary NET1-NEO1 complex structure resembles the overall architecture of the binary DCC-NET1 complex (Xu et al., 2014), revealing two highly conserved interfaces—a high-affinity interaction site of NET1 with the DCC/NEO1-FN4 domain (interface 1) and a lower affinity interaction with the DCC/NEO1-FN5 domain (interface 2) —that are both crucial for interactions and function. Both arrangements suggest NET1-mediated clustering of DCC/NEO1 is important for downstream signaling. Our analysis of binary NET1-receptor complexes supports a conserved binding mode and mechanism of signaling activation between NET1 and both NEO1 and DCC.

We previously showed that the binding affinity of RGMB for DCC is approximately 1,000-fold lower than for NEO1 (Bell et al., 2013). Despite sharing over 80% similarity with the NEO1 minimal RGM-binding region (NEO1-FN_5-6_), DCC lacks a RGM interface loop present in NEO1-FN5 and therefore acting as a specificity determinant for NEO1-RGM binding. In agreement with the lack of a physiologically relevant DCC-RGM interaction, here we show that knockout of DCC in CNs has no effect on RGMA-induced growth cone collapse nor on the ability of NET1 to modulate this effect. DCC deletion did not affect NET1-mediated SVZ-neuroblast migration nor the ability of RGMA or RGMB to inhibit this effect. This supports the idea that inhibition by RGMs is likely attributed to the ternary NEO1-NET1-RGM complex. The divergent evolution of NEO1 alongside DCC may be explained by a requirement for the ternary NEO1-NET1-RGM complex in signal transduction, compartmentalized separately from DCC to specifically regulate inhibition of RGM signaling.

RGM and NET1 expression patterns frequently overlap, e.g. during neural tube closure or in the more developed central nervous system (Kee et al., 2008; Moon et al., 2011; Muramatsu et al., 2011; O’Leary et al., 2013; Wilson and Key, 2006) (Figures 3D and S54). Initial work implied competitive binding between NET1 and RGMA on NEO1 (Conrad et al., 2007). Our data show that instead both ligands can simultaneously bind NEO1 to form a “silencing” complex that blocks downstream signaling and function. Our study, and the work of others, supports a model in which this ternary complex is important for regulating and fine-tuning NEO1-NET1-RGM signaling output. For example, previous work showed that NET1 can inhibit the repulsive effect of RGMA on migrating cortical interneurons (O’Leary et al., 2013). Our study also reveals that NET1 can silence RGMA-mediated growth cone collapse in CNs, requiring NEO1 (Okamura et al., 2011; van Erp et al., 2015) but not DCC. We also show that NET1 can inhibit RGMA-induced inhibition of CN migration *in vivo*. Importantly, NET1 and RGMA are co-expressed in the MGE and in various (intermediate) target regions of CN axons (Livesey and Hunt, 1997; van den Heuvel et al., 2013), and thus interneurons and CN axons are exposed simultaneously to both ligands during their migration or growth *in vivo*, emphasizing the biological relevance of our findings. Vice-versa, RGMA and RGMB inhibited the positive effects of NET1 on SVZ-neuroblast migration. SVZ-neuroblasts express NEO1 and migrate from the VZ through the RMS towards the olfactory bulb in a NET1-dependent and DCC-independent manner, encountering RGMs *en route*. NET1-mediated SVZ-neuroblast migration is inhibited by RGMA in a dose-dependent manner *in vitro* (Figures 5 and 6). These data suggest that NET1 and RGMA and RGMB functionally interact and that their simultaneous presentation leads to reciprocal silencing of their downstream effects, in line with the idea that the ternary complex acts to silence receptor signaling.

Currently, anti-RGMA antibodies are in clinical trials for the treatment of spinal cord injuries and progressive multiple sclerosis (Demicheva et al., 2015; Mothe et al., 2017; Nakagawa et al., 2019), and antibodies against RGMC decrease the level of hepcidin (the key regulator of iron homeostasis), offering promising therapeutic candidates for patients suffering from anemia of inflammation (Kovac et al., 2016). Signal activation by NET1 is linked to cellular self-renewal through Wnt and MAPK pathway elements (Huyghe et al., 2020), and interference with receptor interactions via a NET1-specific antibody triggers cell death in NET1-expressing tumors (Grandin et al., 2016). Central to generating regenerative therapeutics against NEO1 is balancing its role as a dependence receptor with axonal growth inhibition, as has recently been identified for RGM peptides that activate NEO1 but block targeting to membrane raft domains (Shabanzadeh et al., 2015; Tassew et al., 2014). Having identified the key interaction domains and interfaces that can be targeted by biologics or small molecules, our structural analyses will help modulate RGM and NET1 signaling in human diseases as well as open new therapeutic avenues via specific modulation of cell surface receptor stoichiometries.

### Limitations of the study

Although our study demonstrates that NEO1, NET1, and RGMs can form a “trimer-of-trimers” super-complex that silences the biological effects of NET1 and RGMs downstream of NEO1, there are a few outstanding issues:

1) Given the broad and overlapping roles of RGMs and NET1, it is plausible that the ternary complex functions more generally; e.g., we show that RGMC (not expressed in the brain) can participate in forming the NEO1-NET1-RGM super-complex (Figures S2G and S2H). Moreover, other receptors bind structurally distinct ligands, e.g., DCC binds NET1 and Draxin (Ahmed et al., 2011; Liu et al., 2018), while Plexins can bind Semaphorins and Slits (Beamish et al., 2018; Delloye-Bourgeois et al., 2015; Seiradake et al., 2016), and further experimental work is needed to establish whether such receptor complexes can form silencing super-complexes.
2) Future work is needed to investigate how the NEO1-NET1-RGM complex is modulated in the cell membrane. It is possible that the complex may exist in an equilibrium that is dependent on the local concentrations of NET1 and RGM or that the complex is endocytosed. Alternatively, complex formation may induce NEO1 cleavage to terminate signaling (e.g., van Erp et al., 2015) or affect localization into lipid rafts (Shabanzadeh et al., 2015). *In vivo* proteomic approaches performed in this study to identify NEO1-interacting proteins detected previously uncharacterized partners, including those involved in synapse formation and function (Figure S8; Table S3). Therefore, another future model to explore is that termination of NEO1-NET1-RGMA signaling in, e.g., axon guidance, may allow activation of subsequent cellular processes such as synapse formation. There is likely also regulation at the level of the individual components of the super-complex. For example, RGMB can be phosphorylated by the extracellular kinase VLK (Harada et al., 2019). The RGMB tyrosine side chain that is phosphorylated by VLK (human Y268) is located opposite the RGMB-NEO1 interface, in a RGMB region that is in proximity to NET1 (Figures S9A and S9B). This suggests that RGMB phosphorylation by VLK may impact on the formation of the ternary NEO1-NET1-RGMB complex. RGMs can also be released into the extracellular matrix via cleavage of their GPI anchor (Zhang et al., 2007) as well as by SKI-1 and Furin proteases resulting in several different RGM fragments (Tassew et al., 2012). Thus, RGMs exhibit a plastic role, with context-dependent RGM fragments capable of signaling in “*trans”* to control NEO1 receptor architectures and downstream signaling.

**Figure S7:**
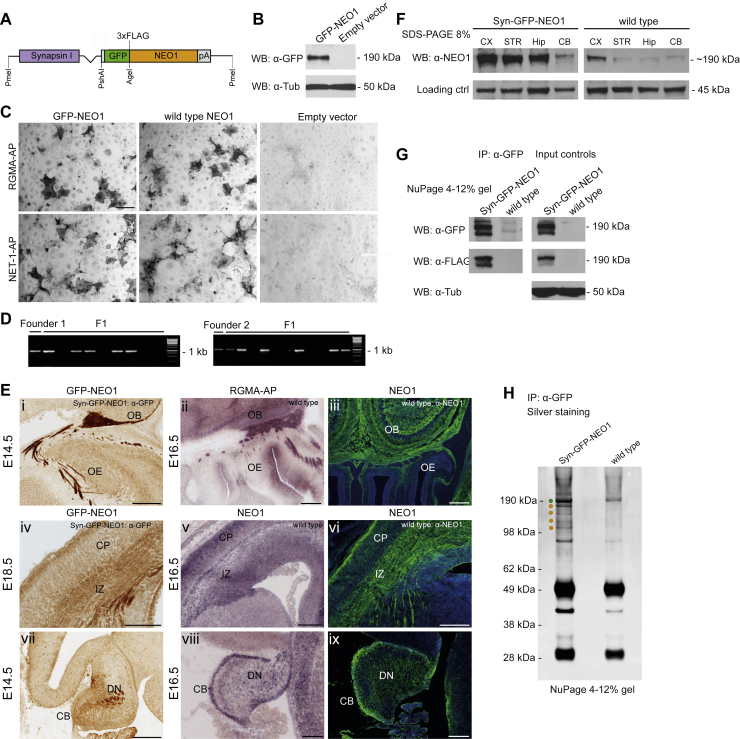
Generation of neuron-specific *NEO1* transgenic mice and *in vivo* proteomics analysis of NEO1-interacting proteins in brain lysates, related to Figure 7 **(A)** Schematic representation of the Syn-GFP-NEO1 fusion DNA fragment containing N-terminally GFP- and 3xFLAG-tagged mouse NEO1 cDNA cloned downstream of the neuron-specific synapsin-I promoter. pA: SV40 late polyadenylation signal. **(B)** Anti-GFP immunoblotting shows expression of GFP-NEO1 in lysate of HEK293 cells transfected with pcDNA3.1-CMV-GFP-NEO1. **(C)** RGMA-AP and Netrin (NET)-1-AP binding to COS-7 cells transfected with pcDNA3.1-CMV-GFP-NEO1 or wild type NEO1 (pCMVXL-6- NEO1). Empty vector (pcDNA3.1)-transfected COS-7 cells do not bind RGMA-AP or NET1-AP. **(D)***Syn-GFP-NEO1* founders 1 and 2, and transgenic offspring (F1) identified by PCR. Scale bar in C and D = 50 μm. **(E)** GFP-NEO1 expression was compared to endogenous NEO1 expression, using anti-GFP (i, iv and vii) and anti- NEO1 (iii, vi and xi) immunostaining, *NEO1 in situ* hybridization (v and viii) and RGMA-AP section binding (ii) on E14.5 sagittal (i, ii, vii-ix) and E18.5 coronal (iii-vi) brain sections of *Syn-GFP-NEO1* (i, iv, vii) and wild type mice (ii, iii, v, vi, viii, ix). Anti-GFP immunostaining is visualized with DAB. Sections iii, vi and ix are counterstained in blue with fluorescent Nissl. (i) GFP-NEO1 expression in the olfactory epithelium (OE) and olfactory sensory neuron (OSN) projections to the olfactory bulb (OB) in *Syn-GFP-NEO1* mice. (ii, iii) Endogenous NEO1 expression in the OE and OSN projections to the OB revealed by RGMA-AP section binding (ii) and anti-NEO1 immunostaining (iii). (iv-vi) Expression of GFP-NEO1 (iv) and endogenous NEO1 (v, vi) in the cortical plate (CP) and cortical projections in the intermediate zone (IZ). (vii-ix) Expression of GFP-NEO1 (vii) and endogenous NEO1 (viii, ix) in the deep nuclei (DN) and axonal projections of the cerebellum (CB). Markers i-ix: 200 μm. **(F)** Anti-NEO1 immunoblotting to detect NEO1 expression in lysates of dissected cortex (CX), striatum (STR), hippocampus (Hip) and cerebellum (CB) of E18.5 *Syn-GFP-NEO1* mice or wild type littermate controls. Anti-NEO1 immunoblotting on brain lysates of *Syn-GFP-NEO1* mice shows GFP-NEO1 and endogenous NEO1 protein. **(G)** Immunoblotting using anti-GFP and anti-FLAG antibodies shows GFP-NEO protein in an anti-GFP *in vivo* pull down experiment on brain lysates of perinatal *Syn-GFP-NEO* mice. **(H)** Silver staining of an anti-GFP experiment on brain lysates of perinatal *Syn-GFP-NEO1* mice. **(H)** Silver staining of an anti-GFP *in vivo* pull down on brain lysates of perinatal *Syn-GFP-NEO1* mice shows GFP-NEO1 protein (green dot) and putative NEO1-interacting proteins (orange dots).

**Figure S8:**
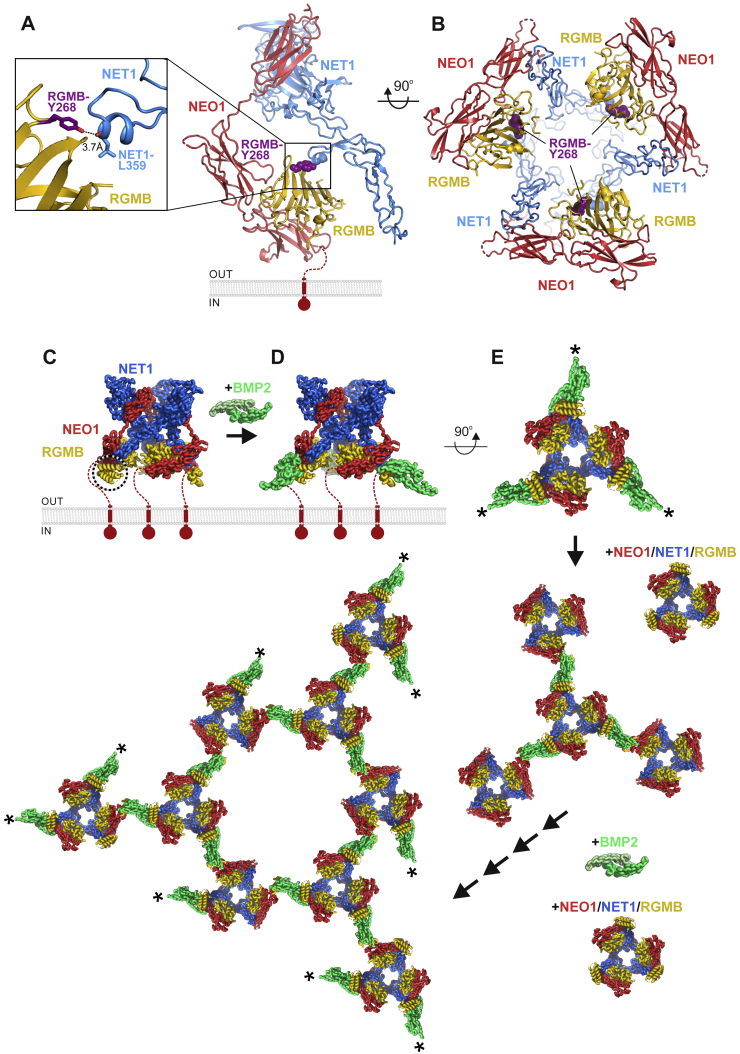
Structural analysis of RGM interactors and consequences for the ternary NEO1-NET1-RGM complex, related to Figures 3 and 7. **(A-C)** Model for BMP2-dependent clustering of the ternary 3:3:3 NEO1-NET1-RGM complex. **(A)** Ribbon presentation of the ternary NEO1-NET1-RGM complex, with modelled RGMB N-terminal domain based on the full-length RGMB structure (PDB ID. 4UI2). One of the three RGMB N-terminal domains essential for BMP binding is marked with a dotted circle. **(B)** The ternary complex containing full-length RGMB harbors three distinct binding sites for the disulfide linked BMP dimer (green) (here shown for BMP2). **(C)** Further addition of the ternary NEO1-NET1-RGM complex and the dimeric BMP2 morphogen can lead to clustering and a continuous arrangement in with RGMB bridges the dimer of BMP2 and the ternary complex. Asterisks mark the “free” RGMB-binding sites on BMP2. **(D, E)** The RGMB VLK phosphorylation site mapped onto the ternary NEO1-NET1-RGM complex structure. **(D)** Ribbon representation of the NEO1-NET1-RGMB protomer structure (color coded as in Figure 1). RGMB tyrosine 268 (Y268) that was previously shown to be phosphorylated by VLK is colored in purple and highlighted. **(E)** Ribbon representation of the NEO1-NET1-RGMB trimer-of-trimers complex. RGMB-Y268 is facing the inside of the ternary complex, and is likely shielded for VLK access.
3) Additional components may participate or interact with the NEO1-NET1-RGM super-complex. For example, in addition to acting as NEO1 ligands, RGMs are crucial co-receptors in the BMP pathway. This is primarily mediated via the interaction of the N-terminal domain of RGM (N-RGM, Figure 1A) with BMP ligands (Healey et al., 2015; Malinauskas et al., 2020; Wu et al., 2012; Yang et al., 2008). For BMP co-receptor function, RGMs are required to signal in “*cis*,” expressed by the same cell as NEO1 (and the BMP receptors). We previously showed that in this scenario RGM can simultaneously bind to BMP ligands and NEO1 (Healey et al., 2015; Malinauskas et al., 2020). This arrangement leads to BMP-dependent clustering of NEO1 at the cell surface, with RGM acting as a physical bridge. The presence of BMP ligands alongside NEO1, NET1, and RGM could facilitate clustering of the ternary 3:3:3 NEO1-NET1-RGM complex (Figures S9C–S9E). Another level of regulation could be achieved by direct protein-protein interactions mediated by the cell surface receptors LRIG (van Erp et al., 2015) and UNC5s (Hata et al., 2009; van den Heuvel et al., 2013) that have been shown to directly interact with and to modulate NEO1.

## STAR★Methods

### Key resources table

**Table:** 

REAGENT or RESOURCE	SOURCE	IDENTIFIER
**Antibodies**		

Mouse anti-His_6_ primary	Takara/Clontech	Cat# 631212, RRID: AB_2721905
HRP-conjugated goat anti-mouse IgG	Merck	Cat# A0168, RRID: AB_257867
Penta·His Antibody, BSA-free	Qiagen	Cat# 34660
Anti-Rho [1D4] monoclonal antibody	University of British Columbia	N/A
Anti-Flag produced in rabbit	Sigma-Aldrich	Cat# F7425 RRID: AB_10571678
Mouse anti-Tubulin	Santa Cruz Biotechnology Inc.	Cat# sc-23948 RRID: AB_10769716
Mouse anti-Tuj1	BioLegend	Cat# 801213 RRID:AB_10063408
Mouse anti-alpha-Tubulin	Sigma-Aldrich	Cat# T5168, RRID: AB_477579
Alexa Fluor 568-Phalloidin	Thermo-Fisher	Cat# A12380
Guinea pig anti-DCX	Merck	Cat# AB2253 RRID: AB_1586992
Goat anti-Neogenin	R&D	Cat# AF1079, RRID: AB_2151002
Rabbit anti-Neogenin	Santa Cruz	Cat# sc-15337 RRID:AB_2150998
Sheep anti-RGMB	R&D	Cat# AF3597, RRID: AB_2179484
Rat anti-Netrin1	R&D	Cat# MAB1109 RRID:AB_2154710
Goat anti-DCC	Santa Cruz	Cat# sc-6535 RRID: AB_2245770
Chicken anti-GFP	AVES	Cat# GFP-1020 RRID: AB_2307313
Rabbit anti-GFP	Invitrogen	Cat# A11122 RRID:AB_221569
Rabbit anti-GFP	Abcam	Cat# ab290 RRID:AB_303395
Mouse anti-FLAG	Stratagene	Cat #200471, RRID: AB_10596509
Goat anti-rabbit, Alexa Fluor 488	Life Technologies	CAT# A11034 RRID: AB_10374301
Donkey anti-Sheep, Alexa Fluor 488	Life Technologies	Cat# A-11015, RRID: AB_2534082
Goat anti-mouse, Alexa Fluor 555	Life Technologies	CAT# A21422 RRID: AB_2536164
Donkey anti-chicken, Alexa Fluor 488	Jackson Immunoresearch	Cat# **703-545-155 RRID:**AB_2340375
Goat anti-Guinea Pig, Alexa Fluor 555	Thermo-Fisher	Cat# A-21435 RRID: AB_2535856
Donkey anti-goat HRP conjugate	Jackson ImmunoResearch	CAT# 705-035-003 RRID: AB_2340390
Goat anti-rabbit HRP conjugate	Jackson ImmunoResearch	CAT# 111-035-003 RRID: AB_2313567
Rabbit anti-sheep HRP conjugate	Abcepta	Cat# ASR1953
Goat anti-rat HRP conjugate	Santa Cruz	Cat# sc-2065 RRID: AB_631756
Streptavidin-HRP conjugate	Sigma-Aldrich	Cat# GERPN1231

**Bacterial and Virus Strains**		

BL21-DE3	NEB	Cat# C2527I
DH5α	Invitrogen	Cat#: 18263012

**Chemicals, Peptides, and Recombinant Proteins**		

Dulbecco’s Modified Eagle’s Medium, high glucose	Sigma-Aldrich	Cat# D5796
D-biotin	Sigma-Aldrich	Cat# B4639
Streptavidin	Sigma-Aldrich	Cat# S4762
Bovine serum albumin	Sigma-Aldrich	Cat# A4503-100G
Polyethylenimine, branched	Sigma-Aldrich	Cat# 408727
Fetal Bovine Serum	Life Technologies	Cat# 10270106
L-Glutamine	Thermo-Fisher	Cat# 25030081
MEM non-essential amino acids	Thermo-Fisher	Cat# 11140050
Neurobasal Medium	Thermo-Fisher	Cat# 21103049
Hank’s balanced salt solution (HBSS)	Life Technologies	Cat# 14170112
Trypsin-EDTA (0.25%), phenol red	Thermo-Fisher	Cat# 25200056
DMEM/F-12	Thermo-Fisher	Cat# 41966-029
DNase I	Roche	Cat# 1284932001
Penicillin-Streptomycin	Thermo-Fisher	Cat# 15140122
B27 serum-free supplement	Thermo-Fisher	Cat# 17504044
Poly-D-Lysine	Sigma-Aldrich	Cat# P0899-100MG
Poly-L-Lysine	Sigma-Aldrich	Cat# P2636
Laminin	Thermo-Fisher	Cat# 23017015
Recombinant Mouse RGM-A Protein	R&D Systems	Cat# 2458-RG-050
Recombinant Mouse Netrin-1 protein	R&D Systems	Cat# 1109-N1/CF
EGF recombinant human protein	Thermo-Fisher	Cat# PHG0311
cOmplete protease inhibitor cocktail	Sigma-Aldrich	Cat# 11697498001
Phosphatase inhibitor cocktail 2	Sigma-Aldrich	Cat# P5726-1ML
Dynabeads protein G Immunoprecipitation Kit	Thermo-Fisher	Cat# 10007D
SuperSignal West Dura Extended Duration Substrate	Thermo-Fisher	CAT# 34076
NuPAGE Novex 4-12% Bis-Tris gradient gel	Invitrogen	Cat# NP0321
GelCode Blue Stain Reagent	Thermo-Fisher	Cat# 24590
FGF-Basic (AA 10-155) Recombinant Human Protein	Thermo-Fisher	Cat# PHG0024
Triton X-100	Merck	Cat# 1086431000
Pyrobest DNA Polymerase	Takara	Cat# R005A
Polybrene infection reagent (10 mg/mL stock = 1,000×)	Merck	Cat# TR-1003-G
Kifunensine class I α-mannosidase inhibitor	Tocris Bioscience	Cat# 3207
16% Formaldehyde solution	Thermo-Fisher	Cat# 28906
VECTASHIELD® Antifade Mounting Medium with DAPI	Vector Laboratories Inc.	Cat# H-1200
Lipofectamine 2000	Thermo-Fisher	Cat# 11668019
Trypsin sequencing grade	Promega	Cat# V5111
10 x Trypsin	PAA	Cat# 11471338
glutaMAX DMEM/F-12	Thermo-Fisher	Cat# 31331-028
Non detergent sulfobetaine (NDSB) 256	Soltec Ventures	CAS No. 81239-45-4
sucrose octasulfate, sodium salt	Toronto Research Chemicals	Cat# S699020-1g
Peptide “TETSQVAPA”	Genscript	Peptide TETSQVAPA
DAPI (4',6-Diamidino-2-Phenylindole, Dihydrochloride)	Thermo-Fisher	Cat# D1306
HEPES	Thermo-Fisher	Cat# 15630080
Ampicillin, sodium salt	Sigma-Aldrich	CAS no. 69-52-3
Kanamycin sulfate	Sigma-Aldrich	Cat# 10106801001
3C protease, His-tagged	Purified from BL21 cells transformed with pET28-3C protease plasmid (STRUBI)	
Albumin from chick egg white	Sigma-Aldrich	Cat# A5503
NBT/BCIP	Roche	11697471001
NuPAGE LDS sample buffer	Invitrogen	Cat# NP0007
MgCl_2_	Sigma-Aldrich	CAS no. 7786-30-3
pregnant mare's serum gonadotropin	Biovendor R&D	Cat# RP1782725000
human chorionic gonadotropin	Biovendor R&D	Cat# RP17825010
Entellan	Merck	Cat# 107960
Mowiol	Sigma-Aldrich	Cat# 81381

**Critical Commercial Assays**		

Vectastain Elite ABC kit	Vector laboratories	Cat# PK-7100 RRID:AB_2336827
NeuroTrace 435/455 Blue Fluorescent Nissl Stain	Invitrogen	Cat#N21479
Proximity ligation assay - Duolink in situ red starter kit mouse/RA	Sigma-Aldrich	Cat# DUO92101
Click-It EdU Cell proliferation kit for imaging, Alexa Fluor 555 dye	Thermo-Fisher	Cat# C10338

**Deposited Data**		

Coordinates and structure factors of the ternary NEO1-NET1-RGMB complex determined by X-ray crystallography	This paper	PDB 7NE0
Coordinates and structure factors of the binary NEO1-NET1 complex determined by X-ray crystallography	This paper	PDB 7NE1
Coordinates of the ternary NEO1-NET1-RGMB complex determined by cryo-EM	This paper	PDB 7NDG
Cryo-EM density map of the ternary NEO1-NET1-RGMB complex	This paper	EMD-12286
Raw movies of the dataset for the ternary NEO1-NET1-RGMB complex determined by cryo-EM	This paper	EMPIAR-10637

**Experimental Models: Cell Lines**		

HEK293	Sigma-Aldrich	Cat# 85120602-1VL RRID: CVCL_0045
HEK 293T	ATCC	Cat# CRL-3216; RRID: CVCL_0063
COS-7	ATCC	Cat# CRL-1651; RRID: CVCL_0224
HEK 293T Lenti-X	Takara/Clontech	Cat# 632180

**Experimental Models: Organisms/Strains**		

Mouse: C57BL/6j	Charles River	027 IMSR_JAX:000664
Mouse*: Dcc*^*fl/fl*^	Anton Berns (Krimpenfort et al., 2012)	N/A
Mouse*: Emx1-IRES-Cre*	The Jackson Laboratories	JAX stock # 005628
Mouse: *Syn-GFP-Neogenin*	This paper	N/A
Mouse: *B6CBAF1/Jico*	Charles River	N/A
Mouse: *Crl:Cd-1(ICR)*	Charles River	022

**Oligonucleotides**		

ISH: Net1-forward *CGACCTCAATAACCCGCACA*	(Brignani et al., 2020)	N/A
ISH: Net1-reverse *CTTGCAACGGTCGCATTCAG*	(Brignani et al., 2020)	N/A
ISH: NEO1-forward *ACACCGTTATCTGGCAATGG*	This paper	N/A
ISH: NEO1-reverse *TTCAGCAGACAGCCAATCAG*	This paper	N/A
genotyping: Neogenin-forward *TTAGACCTTGGTCCCACCATGTTCAAGATCCTGCTG*	This paper	N/A
genotyping: Neogenin-reverse *TCGACCGGTCTTGTCATCGTCATCCTTGTAATCGATATC*	This paper	N/A

**Recombinant DNA**		

Plasmid: pCX-GFP	Alain Chédotal (Zelina et al., 2014)	N/A
Plasmid: pCMV-mycDDK-RGMA	Origene	Cat# MR206975
Plasmid: pCAG:mNtn1-/3xGS/mCherry	Custom made at Vector Builder (Brignani et al., 2020)	Vector ID: VB190710-1048nbz
Plasmid: pSuper-shNeogenin	(van Erp et al., 2015)	N/A
Plasmid: pSuper-Scrambled	(van Erp et al., 2015)	N/A
Plasmid: pcDNA3.1-Syn-GFP-Neogenin	This paper	N/A
Plasmid: pCMVXL-6-Neogenin	Gift from Denise Davis	N/A
Plasmid: PCI-Syn-GlyS267Q	Gift from Manfred Kiliman	N/A
Plasmid: pcDNA3.1(-)/myc-his	Invitrogen	
Plasmid: pRK5-DR/GABA(A)a1	Gift from Guus Smit	N/A
Plasmid: APtag5-RGMA-AP	Gift from Thomas Skutella	N/A
Plasmid: pcDNA3.1-Netrin-1-AP	Gift from Kun-Liang Guan	N/A
Plasmid: AP-Fc	Gift from Roman Giger	N/A
Plasmid: pHR-CMV-TetO2	(Elegheert et al., 2018)	N/A
Plasmid: pHLsec	(Aricescu et al., 2006)	N/A
Plasmid: pHLsec-eNEO1	(Bell et al., 2013)	N/A
Plasmid: pHLsec-NEO_FN56_	(Bell et al., 2013)	N/A
Plasmid: pHLsec- RGMA_ECD_	(Bell et al., 2013)	N/A
Plasmid: pHLsec- RGMB_ECD_	(Bell et al., 2013)	N/A
Plasmid: pHLsec- RGMC_ECD_	(Bell et al., 2013)	N/A
Plasmid: pHLsec- RGMB_ECD_-A186R	(Bell et al., 2013)	N/A
Plasmid: pHLsec-RGMB_ΔN_	(Healey et al., 2015)	N/A
Plasmid: pHLsec-NET1_ΔNTR_	This paper	N/A
Plasmid: pHLsec-NET1_FL_	This paper	N/A
Plasmid: pHLsec-NET1_ΔNTR_(Interface-1)	This paper	N/A
Plasmid: pHLsec-NET1_ΔNTR_(Interface-2)	This paper	N/A
Plasmid: pHLsec-NET1_FL_(Interface-1)	This paper	N/A
Plasmid: pHLsec-NET1_FL_(Interface-2)	This paper	N/A
Plasmid: pHLsec- NEO_FN456_	This paper	N/A
Plasmid: pHLsec- NEO1_FL_	This paper	N/A
Plasmid: pHLsec- DCC_FN56_	This paper	N/A
Plasmid: pHLsec- DCC_FN456_	This paper	N/A
Plasmid: pHLsec- DCC_FL_	This paper	N/A
Plasmid: pHLsec- RGMB_FL_	This paper	N/A
Plasmid: pHLsec- RGMB_core_	This paper	N/A
Plasmid: pHLsec- RGMB_FL_-A186R	This paper	N/A
Plasmid: pET28-3C-protease	This paper	N/A
Plasmid: pHR-CMV-TetO2-NET1_ΔNTR_	This paper	N/A

**Software and Algorithms**		

autoBUSTER	(Bricogne et al., 2011)	https://www.globalphasing.com/buster/
REFMAC5	(Murshudov et al., 2011)	http://www.ccp4.ac.uk/download
PHASER	(McCoy et al., 2007)	http://www.ccp4.ac.uk/download
PDBsum	(Laskowski, 2001)	http://www.ebi.ac.uk/pdbsum
PISA	(Krissinel and Henrick, 2007)	http://www.ebi.ac.uk/pdbe/pisa/
Coot	(Emsley and Cowtan, 2004)	http://www2.mrc-lmb.cam.ac.uk/personal/pemsley/coot/
PyMOL	(Schrodinger, 2010)	https://www.pymol.org/
Consurf	(Ashkenazy et al., 2010)	http://consurf.tau.ac.il/2016/
ATSAS	(Petoukhov et al., 2012)	https://www.embl-hamburg.de/biosaxs/software.html
ScÅtter	(Rambo and Tainer, 2013)	http://www.bioisis.net/
MolProbity	(Davis et al., 2007)	http://molprobity.biochem.duke.edu/
AllosMod-FoXS	(Guttman et al., 2013)	http://modbase.compbio.ucsf.edu/allosmod-foxs/
MultiFoxs	(Schneidman-Duhovny et al., 2016)	https://modbase.compbio.ucsf.edu/multifoxs/
Scrubber2	BioLogic Software	http://www.biologic.com.au/
PRIVATEER	(Agirre et al., 2015a)	https://github.com/agirre/privateer
Sedfit	(Brown and Schuck, 2006)	http://www.analyticalultracentrifugation.com/default.htm
ImageJ	(Schneider et al., 2012)	https://imagej.nih.gov/ij/download.html
Clustal Omega	(Chojnacki et al., 2017)	https://www.ebi.ac.uk/Tools/msa/clustalo/
GraphPad Prism	GraphPad Software	http://www.graphpad.com/scientific-software/prism/
ASTRA 6	Wyatt	https://www.wyatt.com/products/software/astra.html
EPU	FEI	https://www.fei.com/software/epu-automated-single-particles-software-for-life-sciences/
RELION 3.1	(Zivanov et al., 2018)	https://www3.mrc-lmb.cam.ac.uk/relion/index.php/Main_Page
cryoSPARC	(Punjani et al., 2017)	https://cryosparc.com
CTFFIND 4.1	(Rohou and Grigorieff, 2015)	https://grigoriefflab.umassmed.edu/ctffind4
UCSF Chimera	(Goddard et al., 2007)	https://www.cgl.ucsf.edu/chimera/download.html
Phenix	(Afonine et al., 2018)	https://www.phenix-online.org/download/
XIA2	(Winter, 2010)	https://xia2.github.io/
Protein Lynx Global Server software	MatrixScience	http://www.matrixscience.com/help/instruments_masslynx.html#PLGS

**Other**		

TALON® Superflow Metal Affinity Resin	Clontech	Cat# 635668
Biacore T200	GE Healthcare	Cat# 28975001
Sensor Chip CM5	GE Healthcare	Cat# BR100012
CNBr-Activated Sepharose 4B	GE Healthcare	Cat# 17043001
Shodex KW-404 size exclusion column	Shodex (Separation & HPLC) Group	Cat# F6989203
Ultra-thin carbon support film, 3nm-on lacey carbon	Agar Scientific	Cat# AGS187-4
Round filter paper for Vitrobot	Agar Scientific	Cat# 47000-100
Amersham Protran western blotting membranes nitrocellulose	Merck	CAT# GE10600002
Superdex 16/60 200 PG HiLoad	GE Healthcare	Cat# 28989335

### Resource Availability

#### Lead Contact

Further information and requests for reagents should be directed and will be fulfilled by the Lead Contact Christian Siebold (christian@strubi.ox.ac.uk).

#### Material Availability

Plasmids generated in this study will be made available on request, but we may require a payment and/or a completed Materials Transfer Agreement if there is potential for commercial application.

#### Data and Code Availability

Atomic coordinates and structure factors for ternary NEO1-NET1-RGMB and binary NET1-NEO1 complexes determined using X-ray crystallography have been deposited in the Protein Data Bank (PDB) with accession codes 7NE0 and 7NE1, respectively. Atomic coordinates for the ternary NEO1-NET1-RGMB complex determined by cryo-EM have been deposited in the Protein Data Bank with accession code 7NDG, and the cryo-EM density map has been deposited in the Electron Microscopy Data Bank with accession code EMD-12286. Raw movies of the dataset for the ternary NEO1-NET1-RGMB cryo-EM complex have been deposited in the Electron Microscopy Public Image Archive (https://www.ebi.ac.uk/pdbe/emdb/empiar/) with accession code EMPIAR-10637. The authors declare no competing financial interests.

### Experimental Model and Subject Details

#### Cell lines

All cell lines used in this study are listed in the Key Resources Table and were cultured under standard growth conditions (37 °C, 5% CO_2_). HEK293T were used for transient mammalian expression using pHLsec vector (Aricescu et al., 2006). HEK293T Lenti-X cells were utilised to generate lentiviruses with the pHR-CMV-TetO_2_ vector (Elegheert et al., 2018), which were subsequently used to infect HEK293T cells and produce stable cell lines. Both cell lines were maintained in Dulbecco’s Modified Eagle’s Medium (DMEM high glucose, Sigma) supplemented with L-glutamine, non-essential amino-acids (Gibco) and 10% fetal bovine serum (FBS, Life Technologies) with no addition of antibiotics. Cells were grown and maintained in standard T75 (75 cm^2^ - LentiX) or T175 (175 cm^2^ - HEK293T) flasks. Cos-7 cells were used for PLA, immunofluorescence staining experiments (transfected with pHLsec vectors) and the AP binding assay (transfected with pCMVXL-6 and pcDNA3.1 vectors). Cos-7 cells were grown in complete medium consisting of DMEM supplemented with 10% (v/v) fetal bovine serum, L-Glutamine and MEM non-essential amino acids. For the PLA and immunofluorescence staining experiments, Cos-7 cells were incubated with purified NET1 and RGMB in complete medium. We used *E. coli* DH5α competent cells for cloning and *E. coli* BL21-DE3 for expression of 3C protease.

#### Primary cultures

Subventricular zone neurospheres (SVZ-NSC) were cultured *in vitro* from *C57BL/6* and *Dcc*^*fl/fl*^*-Emx1-IRES-cre* mice. These were used for migration-, differentiation-, and proliferation assays. Isolation SVZ-NSCs was performed as described previously (Guo et al., 2012). All SVZ-NSCs were cultured under standard growth conditions (37 °C, 5% CO_2_) in T25 flasks, in glutaMAX DMEM/F-12 (Thermo-Fisher) supplemented with 20 ng/ml EGF (Thermo-Fisher), 20 ng/ml FGF (Thermo-Fisher), B-27 (Thermo-Fisher) and penicillin/streptomycin (Thermo-Fisher). To induce differentiation SVZ-NSCs were cultured in neural differentiation medium consisting of Neurobasal medium (Thermo-Fisher) supplemented with 200 mM L-glutamine (Thermo-Fisher), 1x pen/strep, B-27 supplement (Thermo-Fisher) and 1.8 mM HEPES (Thermo-Fisher). For the growth cone collapse assays cortical neurons were cultured using trypsin-dissociated P0 mouse cortex from *C57BL/6* and *Dcc*^*fl/fl*^*-Emx1-IRES-cre* mice. Neurons were cultured in Neurobasal medium (NB; Thermo-Fisher) containing 2 mM L-glutamine (PAA), 1x penicillin/streptomycin (pen/strep, Thermo-Fisher), and B-27 supplement (Thermo-Fisher) on 100 μg/ml poly-D-lysine (Sigma-Aldrich) and 40 μg/ml laminin (Thermo-Fisher) coated, acid-washed coverslips in 12-wells plates under standard growth conditions (37 °C, 5% CO_2_).

#### Mouse lines

All mice were housed at 21 ± 2 °C and 40% - 70% humidity, on a wood-chip bedding supplemented with tissue on a 12h/12h day/night cycle with lights off at 19 hr. Animals were fed *ad libitum*. All experiments were approved by the Animal Ethics Committee of Utrecht University (Dierexperimenten Ethische Commissie) (CCD license: AVD115002016532) and conducted in agreement with Dutch laws (Wet op de Dierproeven, 1996; revised 2014) and European regulations (Guideline 86/609/EEC; Directive 2010/63/EU). Pregnant mothers were housed individually from the moment of observation of the plug (E0.5). In all experiments gender was not considered. *C57BL/6j (Charles river)* mice were used for *in utero* electroporation, brain lysate analysis, co-immunoprecipitation, *in situ* hybridization and immunohistochemistry. To delete DCC from the cortex and subventricular zone, *Emx1-IRES-cre* mice (The Jackson Laboratory, JAX stock #005628) were crossed with *Dcc*^*fl/fl*^ mice (Krimpenfort et al., 2012) (a gift from Anton Berns). *Syn-GFP-NEO1* mice were generated by pronuclear injections executed in the Central Laboratory Animal Facility (GDL, Utrecht University). Before injection, a 10.1 Kb DNA fragment containing the *Syn-GFP-NEO1* cassette was PmeI-cut from the *pcDNA3.1-Syn-GFP-NEO1* vector and isolated by agarose gel electrophoresis and electro-elution, followed by phenol-chloroform extraction and ethanol precipitation. DNA was injected into male pronuclei of fertilized eggs isolated from super-ovulated *B6CBAF1/Jico* mice (Charles River). Superovulation was induced by intraperitoneal (i.p.) injection of 5IU pregnant mare's serum gonadotrophin (PMSG; Folligonan), followed by injection of 5IU human chorionic gonadotrophin (hCG; Chorulon) 42-48 hrs later. Super-ovulated females were immediately mated with appropriate stud males. Microinjections were performed with a Narishige IM-300 microinjector. After injection of DNA into the pronucleus, embryos were cultured overnight in M2 medium (Sigma-Aldrich) in a humidified atmosphere with 5% CO_2_ at 37 °C. The next day, 2-cell stage embryos were implanted into Crl:CD-1(ICR) (Charles River) foster mothers. 15-20 Embryos were transferred into one oviduct of each recipient mouse. Transgenic founders were selected by PCR genotyping, using the following primers; FW, 5’-*TTAGACCTTGGTCCCACCATGTTCAAGATCCTGCTG*-3’; and RV, 5’-*TCGACCGGTCTTGTCATCGTCATCCTTGTAATCGATATC*-3’, and backcrossed with *C57BL/6* (Charles River) females to generate stable transgenic mouse lines.

### Method Details

#### Expression and purification of recombinant proteins

List of cDNAs and construct boundaries for secreted protein production: human NET1 (GenBank ID U75586; NET1_ΔNTR_: 25-453, NET1_FL_: 25-604), mouse NEO1 (GenBank ID NM_001042752.1, eNEO1: 42-1117, NEO1_FN56_:867-1117, NEO1_FN456_: 766-1117, NEO1_FL_: 42-1465), human DCC (GenBank ID AC011155; DCC_FN56_: 842-1097, DCC_FN456_: 720-1097, DCC_FL_: 26-1447), chicken RGMA (GenBank ID AY128507; RGMA_ECD_: 29-403), human RGMA (GenBank ID AL136826; RGMA_ECD_: 47-423), human RGMB (GenBank ID AK074887; full-length GPI-anchored RGMB: 50-437, RGMB_ECD_: 53-412, RGMB_ΔN_: 140-414, RGMB_core_: 137-334) and human RGMC (GenBank ID AY372521; RGMC_ECD_: 36-400).

##### Construct generation

All constructs, C-terminally fused with a hexahistidine (His_6_; all NEO1, DCC and RGM constructs), a BirA-recognition sequence (NET1 and RGM constructs, used for SPR), 1D4 epitope tag (that binds the Rho 1D4 antibody (Molday and Molday, 2014; Oprian et al., 1987); NET1_ΔNTR_), or N-terminal His_6_-SUMO-3C (NET1_ΔNTR_ and NET1_FL_ mutants) or FLAG-tag (NEO1_FL_, DCC_FL_ and full-length GPI-anchored RGMB), or N-terminal FLAG and C-terminal His_6_ (RGMB_ECD_ WT and A186R mutant (Healey et al., 2015)) were cloned into the pHLsec vector for transient transfection (Aricescu et al., 2006). Additionally, for SAXS, AUC and explant experiments, NET1_ΔNTR_ and NET1_FL_ constructs were cloned into the pHR-CMV-TetO_2_ vector for lentiviral transduction and stable cell line generation, with an N-terminal His_6_-SUMO-3C tag (Elegheert et al., 2018). This information is summarized in the ‘protein constructs, vectors and experimental uses’ portion of this section.

##### Cell culture

For large-scale expression, secreted constructs were expressed in HEK-293T cells. For transient transfection, cells were grown in roller bottles for 3 days at 37^o^C, 5% CO_2_ in DMEM (Thermo-Fisher) supplemented with 10% FBS (Life Technologies), 2mM L-Glutamine (Thermo-Fisher) and 0.1 mM NEAA (Thermo-Fisher) (’10% FBS/DMEM’) to confluency, transfected with 2 mg/L of pHLsec plasmid using PEI and exchanged into 2% FBS/DMEM. In the case of Lentivirally-transduced stable cell lines, HEK-293 LentiX cells were grown to confluency in 6-well dishes under conditions described above and co-transfected with 4 μg of DNA comprising pHR-CMV-TetO_2_ plasmid, pMD2.G (envelope plasmid) and psPAX2 (packaging plasmid) in a 1:1:1 ratio using PEI (Elegheert et al., 2018) and exchanged into 2% FBS. After 3 days, lentiviral particles were harvested from the supernatant, filtered (0.45 μm) and used to transduce confluent HEK-293T cells in a 6-well dish. Polybrene (Merck) was added at a final concentration of 10 μg/mL to promote virus:host fusion. Stable cell lines generated in this way were expanded to large-scale culture in roller bottles, grown to confluency in 10% FBS (3 days, 37 ºC, 5% CO_2_) and subsequently exchanged into 2% media.

All expression was performed in the presence of kifunensine (1 mg/L), a class I α-mannosidase inhibitor (Chang et al., 2007; Zhao et al., 2011), added during exchange of cell into low serum. Cells were maintained at 37 ºC throughout (besides all NET1_FL_ constructs, which were expressed at 30 ºC following exchange into 2% FBS/DMEM). Conditioned media was collected five days post-transfection, clarified by centrifugation and filtered (0.22 μm).

##### Protein purification – His_6_-tagged proteins

For His_6_-tagged proteins (both C-His_6_, N-His_6_-SUMO-3C and N-FLAG/C-His_6_), conditioned media was dialyzed against 10 volumes of PBS (48h, 4 °C) and proteins were purified by cobalt-affinity chromatography with TALON beads (Clontech). In order to produce RGMB_core_, we engineered a human rhinovirus 3C cleavage site between amino acid residues 334 and 335 (Bell et al., 2013). RGMB_CORE_ and N-His_6_-SUMO-3C-NET1 constructs were first purified as the other His_6_-tagged proteins, cleaved with 3C protease (His_6_ tagged; 12h, 21°C) and subjected to a second Talon bead purification from which the flow through (unbound fraction) was collected.

##### Protein purification – NET1_ΔNTR_-1D4

1D4-tagged NET1_ΔNTR_ was purified directly from conditioned media incubated with purified Rho-1D4 antibody (University of British Columbia) coupled to CNBr-activated sepharose beads (GE Healthcare). Protein-bound beads were washed extensively with 50 mM HEPES pH7.5, 300 mM NaCl and eluted overnight in 50 mM HEPES pH 7.5, 1 M NaCl, 500 μM “TETSQVAPA” peptide (Genscript).

##### Protein purification – Size Exclusion Chromatography

All affinity-purified protein samples were subject to a final purification step via size-exclusion chromatography (SEC) (Superdex S200 16/60 column, GE Healthcare) in 10 mM HEPES, pH 7.5 and 150 mM (1 M for NET1_ΔNTR_) NaCl. N-FLAG/C-His_6_ RGMB_ECD_ constructs purified for *cis* PLA assays were purified in PBS, 10% glycerol.

##### Protein constructs, vectors and experimental uses

NEO1_FN456_ was transiently expressed from pHLsec with a C-His_6_ tag for crystallization, SPR (as analyte), AUC and SAXS experiments. NEO1_FN56_, eNEO1, DCC_FN456_ and DCC_FN56_ were transiently expressed from pHLsec with C-His_6_ tags for use in SPR experiments (as analytes). NEO1_FL_ and DCC_FL_ were transiently expressed from pHLsec with N-FLAG tags for cell surface staining experiments.

NET1_ΔNTR_ was transiently expressed from pHLsec with a C-1D4 tag for crystallization experiments. NET1_ΔNTR_ was also transiently expressed from pHLsec with an N-His_6_-SUMO-3C for SAXS experiments and growth cone collapse assays. NET1_ΔNTR_ was additionally expressed in stable cell lines generated using the pHR-CMV-TetO2 vector with an N-His_6_-SUMO-3C tag for AUC experiments (Elegheert et al., 2018) containing its native signal sequence. The NET1_ΔNTR_ ‘Interface-1’ and ‘Interface-2’ mutants were transiently expressed in pHLsec with N-His_6_-SUMO tags for AUC experiments and growth cone collapse assays. NET1_ΔNTR_ (wild type and mutants) was transiently expressed from pHLsec with C-AviTag3 for SPR experiments (as ligands).

NET1_FL_ was transiently expressed from pHLsec with C-AviTag3 for SPR experiments (as ligand). NET1_FL_ (wild type) was expressed in stable cell lines generated using the pHR-CMV-TetO2 vector with an N-His_6_-SUMO tag for SVZ-NSC culture assays. NET1_FL_ ‘Interface-1’ and ‘Interface-2’ mutants were transiently expressed from pHLsec with N-His_6_-SUMO tags for SVZ-NSC culture assays.

RGMB_CORE_ was transiently expressed from pHLsec with a C-terminal 3C-His_6_ tag for crystallization experiments. RGMB_ECD_ was transiently expressed from pHLsec with a C-His_6_ tag for SAXS, SPR (as analyte), and AUC experiments. RGMA_ECD_ was transiently expressed from pHLsec with a C-His_6_ tag for SPR experiments (as analyte). Full-length GPI-anchored RGMB was transiently expressed from pHLsec with an N-FLAG for *trans* PLA experiments. RGMB_ECD_ WT and A186R were transiently expressed from pHLsec with N-FLAG and C-His_6_ tags for *cis* PLA experiments.

#### Mutagenesis and protein engineering

Site-directed mutagenesis and protein engineering were carried out using a two-step overlap-extension PCR strategy (Heckman and Pease, 2007) and the resulting PCR products were cloned into pHLsec-derived vectors (Aricescu et al., 2006). To produce the core domain of RGMB lacking both the N- and C-terminal regions (RGMB_CORE_: 137-334), a human rhinovirus 3C cleavage site (LEVLFQGP) was engineered between amino acid residues 334 and 335 of RGMB (SAILG-HSLPR)

#### Protein crystallization and data collection

Crystallization trials (100 nl protein solution plus 100 nl reservoir solution) were set up in 96-well Greiner plates in sitting-drop vapor-diffusion format using a Cartesian Technologies robot (Walter et al., 2005). Crystallization plates were maintained at 21 °C and imaged in a Formulatrix R1000 storage vault (Mayo et al., 2005).

Both the binary NEO1_FN456_:NET1_ΔNTR_ and ternary NEO1_FN456_:NET1_ΔNTR_:RGMB_CORE_ complexes were formed by mixing purified single components in equimolar amounts, dialyzed against 10 mM HEPES, pH 7.5 and 150 mM NaCl (24h, 4 °C) and purified via SEC (Superdex 200 16/60, GE Healthcare) with a running buffer of 10 mM HEPES, pH 7.5 and 150 mM NaCl. Peak fractions containing the respective complex were supplemented with NDSB-256 (Hampton research) to a final concentration of 0.2 M and concentrated to 7 mg/ml. Prior to crystallization, protein complexes were treated with endoglycosidase F1 (1 μg endoglycosidase F1/mg target glycoprotein, 1h, 37 °C) and supplemented with 3 mM sucrose octasulfate (SOS) (Toronto Research Chemicals).

NEO1_FN456_-NET1_ΔNTR_ complex crystals were grown in a reservoir solution containing 0.2 M ammonium nitrate, 20% PEG 3350, 40 mM potassium/sodium tartrate and was cryoprotected with this reservoir solution supplemented with 30% (v/v) ethylene glycol.

NET1_ΔNTR_-NEO1_FN456_-RGMB_CORE_ crystals were obtained in reservoir solution containing 0.1 M imidazole/MES, pH 6.5, 10% (w/v) PEG 8000, 20% (v/v) ethylene glycol and 30 mM sodium nitrate, sodium phosphate, ammonium sulphate, respectively, and were cryoprotected with 10% (v/v) ethylene glycol.

X-ray data were collected at 100 K at a wavelength of 0.9763 Å on beamline I03 at the Diamond Light Source, UK, and processed and scaled with XIA2 (Evans and Murshudov, 2013; Kabsch, 2010; Winn et al., 2011; Winter, 2010). X-ray data collection and refinement statistics are shown in Table S1.

#### Structure determination and refinement

The binary NEO1_FN456_:NET1_ΔNTR_ complex was solved by molecular replacement in PHASER (McCoy et al., 2007) using the structure of the fifth and sixth fibronectin type III domains of mouse NEO1 (PDB ID: 4BQ6 (Bell et al., 2013)), the fourth domain of human NEO1 (PDB ID: 1X5I) and chicken NET1_ΔNTR_ (PDB ID: 4PLM (Xu et al., 2014)) as search models. The ternary NET1_ΔNTR_-NEO1_FN456_-RGMB_CORE_ complex was determined using coordinates from the binary NEO1_FN456_:NET1_ΔNTR_ complex from this study and human RGMB (PDB ID: 4BQ6 (Bell et al., 2013)) as molecular replacement search models in PHASER (McCoy et al., 2007).

Structures were refined using initial rounds of refinement in REFMAC5 (Murshudov et al., 2011) and completed using autoBUSTER (Bricogne et al., 2011). This was interspersed with rounds of manual model building in COOT (Emsley and Cowtan, 2004). Refinement statistics are presented in Table S1 and Methods S1.

#### Model analysis

Stereochemical properties were assessed using MolProbity (Davis et al., 2007) and are presented in Table S1. PRIVATEER (Agirre et al., 2015b) was used to validate carbohydrate structures. Superpositions were calculated using the program PyMOL (Schrodinger, 2010), which was also used for preparation of images for figures. Buried surface areas were calculated using the PDBsum (Laskowski, 2001) and PISA (Krissinel and Henrick, 2007) webservers with a probe radius of 1.4 Å.

#### Cryo-EM sample preparation and data collection

The ternary NEO1_FN456_-NET1_ΔNTR_-RGMB_ECD_ complex was purified by SEC on a S200 10/300 Increase column with a running buffer of 10 mM HEPES pH 7.5, 150 mM NaCl, 2 mM CaCl_2_, 1 mM SOS, 0.01% NaN_3_ at 4 °C (Figure S2). The peak fraction containing the ternary complex was diluted to 0.07 mg/ml in SEC buffer. Lacey carbon grids with 3nm ultrathin carbon support film were glow discharged for 30 seconds at high RF level using Harrick Plasma Cleaner, model PDC-002-CE, and then 3.5 μl of the sample was pipetted per grid. Excess protein was blotted away for 3 seconds using filter paper (round filter paper for Vitrobot from Agar Scientific, catalogue number 47000-100) and Vitrobot Mark IV (Thermo Fisher Scientific) (relative force -15) at 95–100% humidity. Grids were plunge frozen in liquid ethane.

Cryo-EM data were collected on a Titan Krios G3i microscope (Thermo Fisher Scientific) operating at 300kV with a 50μm C2 aperture and Volta phase plate (Thermo Fisher Scientific), at the Division of Structural Biology, University of Oxford. Movies were recorded using a FEI Falcon III direct electron detector in electron counting mode using EPU software at a nominal magnification of 96000x, corresponding to a physical pixel size of 0.85 Å/pixel. A total dose of 40 e^–^/Å^2^ was used at a dose rate of 0.77 e^–^/pix/sec. Detailed acquisition parameters are listed in supplemental information (Table S2).

#### Cryo-EM data processing and model refinement

In total 1635 movies were collected and drift correction, beam-induced motion and dose-weighting were performed with MotionCor2 RELION 3.1 (Zivanov et al., 2018) for 1635 movies. Contrast transfer function (CTF) was estimated using CTFFIND 4.1 (Rohou and Grigorieff, 2015) implemented in RELION. 280158 particles were picked using Warp (Tegunov and Cramer, 2019). 2D classification in cryoSPARC v2 (Punjani et al., 2017) were performed for these particles and best 2D class averaged with 100674 particles were used to generate ab-initio 3D model with C3 symmetry. C1 symmetry did not generate reasonable 3D model. All Warp picked particles were used for 3D classification in cryoSPARC v2 and the best class with 177056 particles was used for refinement in RELION 3.1 with initial model generated in cryoSPARC v2. Bayesian particle polishing improved the resolution to 5.44 Å, however the map for RGMB was very weak. Last step of 3D classification without alignment with T regularization parameter set to 16 was performed and gave one class with more continuous map for RGMB, which was refined to 5.98 Å resolution, as estimated using the Fourier shell correlation (FSC) = 0.143 criterion.

The crystal structure of the ternary NEO1-NET1-RGMB (3:3:3) complex was placed into 5.98 Å map manually and was initially fitted as 3 rigid bodies using UCSF Chimera (Goddard et al., 2007). Each rigid body comprised the C-terminal domain of RGMB in complex with the FN domains 5–6 of NEO1 plus NET1 in complex with the FN domain 4 of NEO1. These 3 rigid bodies could be readily refined in real space using PHENIX 1.18.2-3874 (Afonine et al., 2018) (MolProbity all-atom clash score 2.41; model-to-map fit, CC_mask 0.48 (Davis et al., 2007)). We further refined the ternary NET1–NEO1–RGMB (3:3:3) complex by splitting it into 6 rigid bodies. 3 rigid bodies out of 6 comprised the C-terminal domain of RGMB in complex with the FN domains 5–6 of NEO1 plus the LE domains 2–3 on NET1. The remaining 3 rigid bodies out of 6 comprised the LE 1-LN domains of NET1 plus the FN domain 4 of NEO1. The refinement of 6 rigid bodies in real space using PHENIX gave better model-to-map fit without introducing any clashes and is presented in this study (MolProbity all-atom clash score 2.22; model-to-map fit, CC_mask 0.55). Linkers between NEO1 FNs 4 and 5–6, and NET1 LNs 1 and 2–3 were manually rebuilt in COOT. A full description of the cryo-EM data collection and structure refinement parameters is presented in Table S2 and the work flow is illustrated in Methods S2.

#### Surface plasmon resonance (SPR).

cDNA inserts for desired SPR ligands were cloned into the pHLsec-Avitag3 vector (Aricescu et al., 2006), leading to the production of proteins carrying a C-terminal biotin ligase (BirA) recognition site (‘Avi-tag’). Constructs were co-transfected into HEK-293T cells in a 6-well dish with pDisplay-BirA-ER (Addgene plasmid 20856; codes for an ER-localized biotin ligase) in a 3:1 pHLsec:pDisplay ratio. Cells were supplemented with 100 μM D-biotin to facilitate Avi-tag biotinylation, and maintained at 37^o^C, 5% CO_2_ for 3 days. Conditioned medium was collected and dialyzed against 2000 volumes of PBS. SPR experiments were carried out using a Biacore T200 machine (GE Healthcare) at 25 °C in SPR running buffer (10 mM HEPES, pH 7.5, 150 mM NaCl, 0.05% (v/v) polysorbate-20 and 1 mg/ml bovine serum albumin (fraction V, Sigma)). Streptavidin (Sigma-Aldrich) was covalently coupled to CM5 sensory chips via amine coupling to a response unit (RU) level of 5000 RU (Johnson et al., 2009; O’callaghan et al., 1999), to which biotinylated ligands were captured to the desired RU level. For each experiment, two analyte binding cycles were performed with buffer injections between each, enabling double referencing of binding responses (Myszka, 1999).

##### Initial NEO1:NET1 interaction study

NET1_ΔNTR_ and NET1_FL_ were immobilized at a level of 900 RU each. Purified NEO1 constructs (eNEO1, NEO1_FN456_) were purified by SEC as described above, and the highest concentration of analyte prepared via mixing 1:1 with a two-fold concentrated stock of SPR running buffer. Injection of 9 samples, prepared by a two-fold dilution series in running buffer from a highest concentration of 8 μM, was performed according to a series of: buffer blank, lowest-to-highest, highest-to-lowest, buffer blank. eNEO1 was injected for 400 s and NEO1_FN456_ for 360 s at a flow rate of 30 μl/min, followed by a 60 s dissociation phase. Surfaces were regenerated using 3 M MgCl_2_, injected for 120 s. Experimental data were processed using program SCRUBBER2 (Biological). Non-linear regression curve fitting was used to fit data to a ‘one-site specific binding’ model (Y = B_max_ × X /(K_d_ + X); X, analyte concentration; B_max_, maximum analyte binding) using GraphPad Prism 7 (www.graphpad.com).

##### NEO1/DCC:NET1 binding site analysis

NET1_ΔNTR_ wild type, ‘Interface-1’ mutant and ‘Interface-2’ mutant were immobilized at a level of 1007, 1027 and 937 RU each. NEO1_FN456,_ NEO1_FN56_, DCC_FN456_ and NEO1_FN56_ were purified by SEC and buffer exchanged into SPR running buffer as detailed above. Injection of 10 samples, prepared by a two-fold dilution series in running buffer from a highest concentration of 50 μM, was performed according to a series of: buffer blank, lowest-to-highest, highest-to-lowest, buffer blank. Analytes were injected for 110 s at a flow rate of 10 μl/min, followed by a dissociation phase of 60 s. Surfaces were regenerated using 3 M MgCl_2_, injected for 120 s. Experimental data were processed using program SCRUBBER2 (Biological). Non-linear regression curve fitting was used to fit data to a ‘one-site specific binding’ model (Y = B_max_ × X /(K_d_ + X); X, analyte concentration; B_max_, maximum analyte binding) using GraphPad Prism 7 (www.graphpad.com)

##### NET1:RGM interaction study

NET1_ΔNTR_ was immobilized at a level of 900 RU. RGMB_ECD_ and RGMA_ECD_ were purified by SEC and buffer exchanged into SPR running buffer as detailed above. Injection of 12 samples, prepared by a two-fold dilution series in running buffer from a highest concentration of 40 μM (RGMB_ECD_) or 25 μM (RGMA_ECD_), was performed according to a series of: buffer blank, lowest-to-highest, highest-to-lowest, buffer blank. RGM_ECD_ analytes were injected for 110 s at a flow rate of 10 μl/min, followed by a dissociation phase of 60 s. Surfaces were regenerated using 3 M MgCl_2_, injected for 120 s. Experimental data were processed using program SCRUBBER2 (Biological). Non-linear regression curve fitting was used to fit data to a ‘one-site specific binding’ model (Y = B_max_ × X /(K_d_ + X); X, analyte concentration; B_max_, maximum analyte binding) using GraphPad Prism 7 (www.graphpad.com)

#### Analytical ultracentrifugation

Sedimentation velocity (SV) experiments were performed at 20 °C using a Beckman Optima XL-1 analytical ultracentrifuge (Beckman Instruments), utilising a scanning absorbance of 280 nm and interference optics. Samples were contained within 12 mm Epon sector-shaped two-channel centerpieces and spun at 400,000 rpm (An60Ti rotor, Beckman Coulter Inc., CA), with 100 sample distribution scans taken in 6 minute intervals, alongside interference optics. Absorbance data for scans were analyzed using the program SedFit (Brown and Schuck, 2006) for size-and-shape distributions [c(*s,fr*), where *fr* is the frictional ratio and for a sphere *fr* = 1 and for other species *fr* > 1](Brown and Schuck, 2006). This enables the plotting of contour plots of c(s,M), where M is the weight of the protein. In all cases, a partial specific volume value of 0.73 ml g^-1^ was used.

For SV-AUC experiments, binary NEO1_FN456_-NET1_ΔNTR_ and ternary NEO1_FN456_-NET1_ΔNTR_-RGMB_ECD_ complexes were assembled via mixing of components in equimolar ratios, using wild type NET1_ΔNTR_ or NET1_FL_, and ‘Interface-1’ and ‘Interface-2’ mutants. Complexes were then dialyzed for 16 hours against a buffer containing 10 mM HEPES pH 7.5, 150 mM NaCl, 2 mM CaCl_2_, and the concentration was subsequently adjusted to 3 mg/ml via dilution with dialysis buffer.

#### Small-angle X-ray scattering (SAXS).

SAXS experiments were performed at beamline B21, Diamond Light Source, UK, at 298K, over a momentum transfer (q) range of 0.01 Å^-1^ < q < 0.45 Å^-1^, where q = 4π sin(θ)/λ, and 2θ is the scattering angle. Samples were injected onto an inline Shodex KW-404 size exclusion column in a running buffer of 10 mM Tris pH7.5, 150 mM NaCl, 3 mM CaCl_2_, 1 mM KNO_3_. Data were collected using a Pilatus 2M detector with a sample-to-detector distance of 4014 mm and a beam energy of 12.4 keV. Protein samples were injected at the following concentrations: NET1_ΔNTR_: 4.0 mg/ml; RGMB_ECD_: 4.0 mg/ml; NEO1_FN456_: 4.0 mg/ml; NEO1_FN456_-NET1_ΔNTR_: 2.4 and 5.9 mg/ml; NEO1_FN456_-NET1_ΔNTR_-RGMB_ECD_: 3.3, 4.0 and 6.0 mg/ml.

Data processing and reduction, alongside calculation of molecular weights from the volume of correlation metric (Vc), was performed using the program Scatter (Rambo and Tainer, 2013). Individual SEC-SAXS frames were loaded into Scatter and protein/buffer regions were selected according to visual inspection of the per-frame scattering intensity plot. Individual frames containing protein were buffer-subtracted, followed by analysis of R_g_ (radius of gyration) values across the peak region of eluted protein complex. In each case, a subset of frames showing a constant R_g_ (+/-1 Å) was selected for further analysis. These frames were scaled against one another and individual Log_10_ intensity plots were inspected to inform removal of any outlier frames. Finally, matching frames were merged together to generate a final data file for further analysis. The reported R_g_ and error values were calculated using PRIMUS (Konarev et al., 2003). Mass determination of the NEO1_FN456_-NET1_ΔNTR_-RGMB_ECD_ complex was based on the scattering data from the highest concentration sample (6.0 mg/ml). At the lower concentrations, ternary complex disassembly led to contamination of the overall dataset by non-3:3:3 complexes.

For modelling of the NET1_ΔNTR_ solution structure, missing residues were first added to the crystal structure using Modeller (Eswar et al., 2003). Then, 50 independent all-atom ensembles of 100 models were generated using Allosmod (Weinkam et al., 2012) and calculation and fitting of theoretical scattering curves was performed using FoXS (Schneidman-Duhovny et al., 2016). This entire procedure was automated via the use of Allosmod-FoXS (Guttman et al., 2013). The best-scoring model was used as input for MultiFoXS (Schneidman-Duhovny et al., 2016) to model flexibility at inter-domain hinge points. This process produced 10000 conformations, followed by scoring of multi-state models fit to experimental scattering data as described above using FoXS.

#### Size exclusion chromatography coupled with multi angle light scattering (SEC-MALS)

SEC-MALS experiments were performed using a Wyatt Dawn HELEOS-II 8-angle light scattering detector (with 663.8 nm laser) and a Wyatt Optilab rEX refractive index monitor linked to a Shimadzu HPLC system comprising LC-20AD pump, SIL-20A autosampler and SPD20A UV/Vis detector. SEC-MALS of the NEO1_FN456_-NET1_ΔNTR_-RGMA_ECD_ complex (1.8 mg ml^-1^, 100 μl) was performed using a Superose 6 Increase 10/300 GL column equilibrated in 150 mM NaCl, 10 mM HEPES pH 7.5, 2 mM CaCl_2_, 1 mM sucrose octasulfate, 0.02% NaN_3_, 0.5 ml min^-1^ flow rate at 21 °C. SEC-MALS of the NEO1_FN456_-NET1_ΔNTR_-RGMB_ECD_ complex (1.0 mg ml^-1^, 100 μl) was performed using a Superdex 200 10/30 column equilibrated in 150 mM NaCl, 10 mM HEPES, pH 7.5, 2 mM CaCl_2_, 1 mM sucrose octasulfate, 0.01% NaN_3_, 0.5 ml min^-1^ flow rate at 21 °C. Scattering data were analyzed and molecular weight was calculated using ASTRA 6 software (Wyatt). Glycosylation of the NEO1_FN456_-NET1_ΔNTR_-RGM_ECD_ complexes was taken into account during calculation of dn/dc value. RGMA-containing ternary complex with 8 Asn-linked glycosylation sites: dn/dc = 0.1810 ml/g; RGMB-containing ternary complex with 7 Asn-linked glycosylation sites: dn/dc = 0.1814 ml/g. dn/dc values for proteins (0.1850 ml/g) and glycans (0.146 ml/g) were taken from (Arakawa and Wen, 2001). To calculate the total molecular mass of glycoprotein complexes, it was assumed that each Asn-linked glycosylation site was attached to a Man_9_GlcNAc_2_ moiety with a mass of 1883 Da.

#### Detection of RGMB and NET1 binding to NEO1

COS-7 cells were transfected with the corresponding plasmids and Lipofectamine 2000. Cells were then re-plated and grown on coverslips for 48h, before they were incubated with fresh medium only as a negative control or with fresh medium containing purified Netrin and purified RGMB where applicable for 2 hours at 37°C. Subsequently, the medium was removed, cells were gently washed with PBS and fixed with 4% PFA for 10 min.

Immunofluorescence staining was performed by blocking the fixed cells with 10% FBS in PBS for 1 hr. All antibodies were diluted in 0.1% FBS in PBS and washes were carried out with PBS. After blocking, cells were incubated with anti-Flag (1:500) and anti-Rho ID4 (1:1000). Following incubation with the secondary antibodies Alexa Fluor 488 (1:1000) and Alexa Fluor 555 (1:1000) the coverslips were mounted onto a microscope slide with Vectashield containing DAPI.

PLA assays were carried out solely with reagents included in the Duolink *in situ* red starter kit apart from both antibodies and mounting medium. This technology enables the detection of specific protein-protein interactions in fixed cells. Detection is based on the recognition of both protein partners with specific antibodies that are being covalently linked to DNA primers. If the proteins of interest are in close proximity (< 40nm), the DNA oligos hybridize and give rise to circular DNA. A rolling-circle amplification (RCA) step with fluorescent probes generates highly enhanced fluorescent foci of sites of protein-protein interaction that can be visualized by fluorescence microscopy (Alam, 2018). Incubations were performed in a humidity chamber. In essence, fixed cells were blocked with blocking solution for 1 h at 37 °C. Cells were then incubated with anti-Flag (1:1000) and anti-Rho ID4 (1:1000) in antibody diluent for 1hr at room temperature, followed by three wash steps with buffer A. The minus and plus DNA probes were diluted in antibody diluent, added to the cells and incubated for 1 h at 37 °C. After three wash steps with buffer A, 5x ligase buffer was diluted in water and complemented with ligase, added to cells and incubated at 37 °C for 30 min. This was followed by three wash steps with buffer A. 5x amplification buffer was diluted in water and complemented with polymerase. This was added to the cells and incubation was at 37 °C for 90 min. Cells were washed two times with 1x and one time with 0.01x buffer B. Finally, the cover slips were mounted on microscope slides using Vectashield containing DAPI. Images were taken with a Leica DMi8 TIRF Microscope and a Hamamatsu Orca Flash 4.0 V2 camera.

#### *pcDNA3.1-Syn-GFP-Neogenin* plasmid construction

For the construction of the *pcDNA3.1-Syn-GFP-NEO1* vector, the *NEO1* coding sequence without signal peptide (aa 46-1492) was PCR-amplified from wild type mouse NEO1 (pCMVXL-6-NEO1; a kind gift of Denise Davis). This fragment was cloned into the blunt-made MluI/NotI sites of the *PCI-Syn-GlyS267Q* plasmid, containing the rat synapsin I promoter (a kind gift of Manfred Kiliman (Hoesche et al., 1993) *Syn-NEO1* fragment was released from the PCI vector backbone by ClaI restriction and ligated into the EcoRV site of *pcDNA3.1* (*pcDNA3.1(-)/myc-his*; Invitrogen). A signal peptide, GFP and 3xFLAG tag were PCR-amplified from the *pRK5-DR/GABA(A)a1* vector (a kind gift of Guus Smit) and ligated N-terminal of the *NEO1* coding sequence into the newly generated restriction sites AgeI and PshAI. For construction of the *pcDNA3.1-CMV-GFP-NEO1* vector, the *GFP-NEO1* fragment was isolated from the *pcDNA3.1-Syn-GFP-NEO1* vector using ClaI and PshA restriction. The restriction sites were made blunt-ended and the *GFP-NEO1* fragment was ligated into the EcoRV site of *pcDNA3.1* (*pcDNA3.1(-)/myc-his*; Invitrogen).

#### Cell culture and transfection for immunofluorescence experiments

COS-7 and HEK293 cells were maintained in high glucose Dulbecco’s modified Eagle’s medium (DMEM; Gibco, Invitrogen) supplemented with 10% (v/v) heat-inactivated fetal bovine serum (FBS; Lonza, BioWhittaker), 2 mM L-glutamine (PAA) and 1x penicillin/streptomycin (pen/strep; PAA), in a humidified atmosphere with 5% CO_2_ at 37 °C. COS-7 cells and HEK293 cells were transfected using polyethylenimine (PEI; Polysciences) (Reed et al., 2006).

#### Dissociated cortical neurons and growth cone collapse

Mouse postnatal day 0 (P0) cerebral cortices were dissected and dissociated in 0.25% trypsin (PAA Laboratories) in DMEM/F12 (Thermo-Fisher) at 37 °C for 15 min. Trypsin was inactivated by adding an equal volume of DMEM/F12 containing 20% fetal bovine serum (FBS, Thermo-Fisher). The tissue was further dissociated by trituration in DMEM/F12 containing 10% FBS and 20 μg/ml DNase I (Roche). Dissociated cortical neurons were cultured in Neurobasal medium (NB; Thermo-Fisher) containing 2 mM L-glutamine (PAA), 1x penicillin/streptomycin (pen/strep, Thermo-Fisher), and B-27 supplement (Thermo-Fisher) on 100 μg/ml poly-D-lysine (Sigma-Aldrich) and 40 μg/ml laminin (Thermo-Fisher) coated, acid-washed coverslips in a humidified atmosphere with 5% CO_2_ at 37 °C. At DIV3, cortical neurons were treated for 30 min with control medium or with medium containing 2 μg/ml mouse RGMA (R&D systems) with or without 3.2 μg/ml NET1_ΔNTR_-WT, or NET1_ΔNTR_, ‘Interface-1’ and ‘Interface-2’ mutants, respectively. To test the effect of DCC deletion on RGMA-induced growth cone collapse, cortical neurons were treated for 30 min with control medium or with medium containing 2 μg/ml mouse RGMA (R&D systems) with or without 3.2 μg/ml NET1 (R&D Systems) at DIV3. In order to test whether RGMB induces growth cone collapse, cortical neurons were treated with 2 μg/ml mouse RGMB (R&D systems). Neurons were then fixed with 4% PFA and 15% sucrose for 20 min at RT and washed with PBS. To visualize neurons and growth cones, immunostaining was performed with anti-TuJ1 (1:1000, BioLegend) and with Alexa-568-tagged phalloidin (1:500, Thermo-Fisher). Immunocytochemistry for DCC and NEO1 were performed with goat anti-Neogenin (1:200, AF1079; R&D systems) and goat anti-DCC (1:500, sc-6535, Santa Cruz) primary antibodies and Alexa Fluor 488 donkey-anti-goat (1:750, Life Technologies) secondary antibody. Images were acquired with a Zeiss Axioscope 2 microscope. Growth cone collapse was quantified for more than 100 neurons per condition, for three to six independent experiments. The criterion for the collapsed growth cones was loss of lamellipodia and the presence of only two or fewer filopodia. Statistical analysis was performed with GraphPad Prism 7 using one-way ANOVA (or two-way ANOVA for *Emx1-Cre;DCC-flox* experiment) followed by Tukey’s multiple comparison test.

#### Subventricular zone neurosphere culture and migration assay

Isolation and culture of mouse subventricular zone neurospheres (SVZ-NSCs) was performed as described previously (Guo et al., 2012). Either wild type C57BL/6j animals or *Emx1-cre;DCC-flox* mice (mutants and control littermates) were used. The cultures were maintained in glutaMAX DMEM/F-12 (Thermo-Fisher) supplemented with 20 ng/ml EGF (Thermo-Fisher), 20 ng/ml FGF (Thermo-Fisher), B-27 (Thermo-Fisher) and penicillin/streptomycin (Thermo-Fisher) and kept in a 5% CO_2_ incubator at 37 °C. For the migration assay, coverslips were coated with 100 μg/ml poly-L-lysine (Sigma-Aldrich) and subsequently with 600 ng/ml protein diluted in glutaMAX DMEM/F-12 (Thermo-Fisher) for 2 hours at 37^o^C. Tested proteins were: negative control (vehicle only), NET1_ΔNTR_-WT, NET1_FL_-WT, NET1_FL_ ‘Interface-1’ mutant and NET1_FL_ ‘Interface-2’ mutant. Diluted protein was discarded and differentiation media containing Neurobasal medium (Thermo-Fisher), 200 mM L-glutamine (Thermo-Fisher), 1x pen/strep, B-27 supplement (Thermo-Fisher) and 1.8 mM HEPES (Thermo-Fisher) was added. SVZ NSCs larger than 120 μm were plated on coated coverslips and incubated for 5 days. To test the effect of RGMA and RGMB, coverslips were coated with 600 ng/ml NET1_FL_ as described. Subsequently, mouse RGMA or RGMB was added to the differentiation media. RGMA was tested at two different concentrations: 1.2 μg/ml (2x RGMA) and 6.0 μg/ml (10x RGMA). For RGMB one concentration was tested: 1.2 μg/ml (2x RGMB). Then, cultures were fixed using 4% PFA and 30% sucrose for 15 min, washed with PBS and blocked with 4% bovine serum albumin (Sigma-Aldrich) and 0.1% Triton X-100 (Merck) at room temperature. Primary antibodies used for immunostaining were mouse-anti-Tuj1 (1:500, Biolegend) and guinea pig-anti-DCX (1:200, Roche). Secondary antibodies used were Alexa Fluor 488 donkey-anti-mouse (1:750, Life Technologies) and Alexa Fluor 555 goat anti-guinea pig (1:750, Life Technologies). Cells were counterstained with DAPI. Additional immunocytochemistry for DCC and NEO1 was performed with the following primary antibodies: goat anti-Neogenin (1:200, AF1079; R&D systems) and goat anti-DCC (1:500, sc-6535, Santa Cruz), and Alexa Fluor 488 donkey-anti-goat (1:750, Life Technologies) as a secondary antibody. Images were acquired on the Axioscope 2 (Zeiss) and analyzed using ImageJ (Schneider et al., 2012). Cells that were outside the neurosphere and positive for TUJ1 and the neuronal migration marker doublecortin (DCX) were considered to be migrating neurons. Each experimental group contained 9 to 18 coverslips, and per coverslip between 4 and 6 SVZ NSCs were imaged. The number of migrating cells per neurosphere per condition was normalized to the vehicle (negative control). Bartlett’s test was used to test for a significant difference between the standard deviations. When the input data did not meet the criteria for Bartlett’s test, the Brown-Forsythe test was performed to test for a significant difference between the standard deviations. For normally distributed data, one-way ANOVA (or two-way ANOVA for *Emx1-Cre;DCC-flox* experiment) and post hoc Tukey’s test were performed to test the difference between groups. For not normally distributed data Kruskal-Wallis test with Dunn’s multiple comparisons test were performed. For the RGMB assay, a paired two-tailed t-test was performed. All statistical analysis was performed using GraphPad Prism (www.graphpad.com). Migration distances of DCX/TUJ1+ cells were analyzed by measuring the distance between the nucleus and the edge of the neurosphere. For the RGMA/RGMB-related assays this was measured for all migrating neurons. For the assays concerning the NET1-ΔNTR and NET1-Interface1 and NET1-Interface2, the migration distance of a randomly selected subset of neurons was analyzed.

#### Neurosphere differentiation and proliferation assay

To test the effect of 1.2 μg/ml (2x RGMA) and 6.0 μg/ml (10x RGMA) RGMA on neurosphere differentiation, an EdU assay was performed. DIV2 SVZ NSCs were plated on 100 μg/ml poly-L-lysine (Sigma-Aldrich)-coated coverslips and were placed on differentiation medium (Neurobasal medium (Thermo-Fisher), 200 mM L-glutamine (Thermo-Fisher), 1x pen/strep, B-27 supplement (Thermo-Fisher) and 1.8 mM HEPES (Thermo-Fisher)) for one day. Then, EdU (ThermoFisher) was added to the medium to a final concentration of 10 μM. One day later, the EdU-containing medium was replaced with differentiation medium and cells were cultured for another 3 days. Cultures were fixed using 4% PFA and EdU was visualized using the Click-It EdU Cell proliferation kit for imaging (Alexa Fluor 555 dye; ThermoFisher), according to manufacturer’s protocol. Images were acquired on the Axioscope 2 (Zeiss) and analyzed using ImageJ (Schneider et al., 2012). The single EdU and DAPI channels were first thresholded. Then EdU-positive and DAPI-positive cells were counted using the Analyze Particles function in ImageJ.

A neuronal proliferation assay was performed to test the effect of 1.2 μg/ml (2x RGMA) and 6.0 μg/ml (10x RGMA) RGMA on neuronal proliferation. The SVZ NSC migration assay was performed as described before except that now small DIV2 SVZ NSC neurospheres were used instead of neurospheres > 120 μm. Immunostaining for TUJ1 was performed as described before. Images were acquired on the Axioscope 2 (Zeiss) and analyzed using ImageJ (Schneider et al., 2012). The single TUJ1 and DAPI channels were first thresholded. Then TUJ1-positive and DAPI-positive cells were counted using the Analyze Particles function in ImageJ. For both assays three biological replicates were performed (n = 3), each technical replicate contained 2 coverslips. To test the differences between conditions one-way ANOVA was performed in GraphPad Prism (version 9.0.0)

#### Analysis of single-cell RNA-seq data of adult SVZ cells

Raw single-cell expression data from ventricular-subventricular zone (V-SVZ) cells from 8-10 week old mice was downloaded (GSE109447) (Mizrak et al., 2019). Data was processed using SCANPY version (1.4.6) (Wolf et al., 2018). The dataset contained 13005 cells and 48529 genes. First, cells were normalized and log-transformed. Second, highly variable genes (HVGs) (minimal mean expression = 0.0125, maximal mean expression = 3, minimal dispersion = 0.5) were selected. Third, principal component analysis (PCA) was performed based on the HVGs. Fourth, a neighbourhood graph was computed using the first 40 PCAs. Based on this a UMAP embedding was computed. Original cluster-specific marker genes defined by the authors were used to label clusters. Next, we plotted Netrin-1 (NET1), RGMA, RGMB and Neogenin (NEO1) expression on the UMAP embedding.

#### *In situ* hybridization

Nonradioactive *in situ* hybridization was performed as described in (Pasterkamp et al., 2007). The probe for NET1 was generated by one step reverse transcription (RT)-PCR from whole mouse brain RNA and following primers: 5’-*CGACCTCAATAACCCGCACA*-3’, 5’-*CTTGCAACGGTCGCATTCAG*-3’. The probe for NEO1 (NM_008684.2: 2087-2587) was generated by using sense primer 5’-*ACACCGTTATCTGGCAATGG*-3’ and antisense primer 5’-*TTCAGCAGACAGCCAATCAG*-3’. cDNA was cloned into *pGEM-T Easy* (Promega) and transcribed with T7 polymerase for antisense and Sp6 polymerase for sense probes. Digoxigenin (DIG)-labelled cRNA probe synthesis was performed by *in vitro* transcription. Embryonic brains were isolated, fixed ON in 4% PFA in PBS (pH 7.4), cryoprotected in 30% sucrose in PBS. Brains were embedded in 7,5% gelatin and 10% sucrose solution in PBS and subsequently frozen in isopentane to be stored in -80 ^o^C for long term use. 20 μm sections were cut on a cryostat, mounted on slides, dried for 2 hr and stored at -80^o^C. Tissue sections were washed 3 x 5 minutes with 1x PBS + 0.1% Tween and subsequently permeabilized for 5 minutes with proteinase K (1:5000 in PBS) and were post-fixed with 4% PFA for 10 minutes at room temperature (RT). For background reduction, sections were acetylated with 0.25% acetic anhydride in 0.1 M triethanolamine and 0.06% HCl for 10 minutes at room temperature. Prehybridization in hybridization mix (50% deionized formamide, 5x SSC, 5x Denhardts, 250 μg/mL tRNA baker’s yeast, 500 μg/mL sonicated salmon sperm DNA) was performed for 2 h at room temperature. Slides were hybridized with 400 ng/ml denatured DIG-labeled probe ON at 68 °C. After briefly washing in 2x SSC, sections were incubated in 0.2x SSC for 2 h at 68 °C. Digoxigenin (DIG)-labeled RNA hybrids were detected using anti-digoxigenin FAB fragments conjugated to alkaline phosphatase (Roche, 1:5000) and stained with NBT/BCIP (Roche). Using ProLong Gold antifade reagent (ThermoFisher), sections were mounted and staining was visualized on a digital slide scanner (Hamamatsu Nanozoomer) or using a Zeiss Axioskop 2 microscope.

#### Immunohistochemistry

Timed pregnant mice were sacrificed by cervical dislocation. Brains of the embryos were isolated and fixed in 4% PFA in PBS. Fixed brains were cryoprotected in 30% sucrose in PBS. After embedding in 7.5% gelatin and 10% sucrose, brains were frozen and stored at -80 ^o^ C. Sections of 20 μm were cut on a cryostat and air-dried for 2 hr. The tissue was blocked with blocking solution consisting of 4% BSA and 0.1% Triton X-100 in PBS for 1 h at RT. Following this, sections were treated with primary antibodies in blocking solution at 4^o^ C ON. The antibodies we used were goat anti-NEO1 (1:200, AF1079; R&D systems) and sheep anti-RGMB (1:200, AF3597; R&D systems). After washing with PBS the next day, sections were incubated with secondary antibodies (1:500, Alexa Fluor 488, Life Technologies) in PBS at RT for 1 hr. Sections were washed and mounted with ProLong Gold antifade reagent (ThermoFisher). Optionally, sections were counterstained with fluorescent Nissl stain (NeuroTrace, Invitrogen) 1:500 for 15 min at RT, washed in PBS and embedded in Mowiol (Sigma-Aldrich). Staining was visualized on confocal laser-scanning microscopy (LSM 880, Zeiss) or using a Zeiss Axioskop 2 microscope.

For anti-GFP DAB immunohistochemistry, sections were washed in TBS (pH 7.4), quenched in 3% H_2_O_2_ and 10% methanol in TBS for 15 min, and incubated in blocking buffer (TBS, pH 7.4, 0.1% Triton X-100 and 0.4% BSA) for 1 hr at RT. Sections were incubated with rabbit anti-GFP antibody (A11122; Invitrogen) 1:2000 in blocking buffer. The next day, sections were washed in TBS and incubated with biotin-labeled secondary antibody 1:500 in TBS containing 0.4% BSA for 1.5 hr at RT. Sections were washed in TBS and incubated with avidin-biotin complex (ABC; Vectastain Elite ABC kit, Vector Laboratories) for 90 min. Then, sections were briefly washed in TBS and incubated with 3.3’-diaminobenzidine (DAB) solution to visualize primary antibody binding. Finally, sections were rinsed twice in 0.05 M phosphate buffer, dehydrated in ascending alcohol concentrations, cleared in xylene and embedded in Entellan (Merck).

#### AP cell binding

COS-7 cells were transfected with wild type mouse NEO1 (*pCMVXL-6-NEO1*), GFP-NEO1 (*pcDNA3.1-CMV-GFP-NEO1*) or pcDNA3.1 (*pcDNA3.1(-)/myc-his*; Invitrogen). After 2 days in culture, culture medium was replaced with HBHA buffer (20 mM HEPES, pH 7.0, 1x Hank’s balanced salt solution (HBSS; GIBCO, Invitrogen) and 0.5 mg/ml BSA) for 15 min at RT. Subsequently, cells were incubated with AP-ligands for 75 min, while gently rotating at RT, followed by 2 washes in HBHA buffer. Then, cells were incubated in fixation solution (20 mM HEPES, pH 7, 60% (v/v) acetone and 3.7% formaldehyde) for 30 seconds, followed by 2 washes in HBHA. HBHA was replaced by HBS (20 mM HEPES, pH 7.0 and 150 mM NaCl) and endogenous phosphatase activity was heat-inactivated by incubation at 65 °C for 90 min. Cells were equilibrated in detection buffer (100 mM Tris-HCl, pH 9.5, 100 mM NaCl and 5 mM MgCl_2_) and bound AP-ligand was visualized by incubation in detection buffer containing levamisole and NBT/BCIP (Roche). The specificity of RGMA-AP ligand binding was determined by competition with excess RGMA protein. Furthermore, no staining was observed for AP alone.

#### Section binding

Sections were fixed by immersion in -20°C methanol for 6 min and rehydrated in TBS+ (TBS, pH 7.4, 4 mM MgCl_2_ and 4 mM CaCl_2_). Section were incubated in blocking buffer (TBS+ and 10% FBS (Lonza, BioWhittaker)) for 1 h at and incubated with 1.5 nM AP-tagged protein-containing medium for 2 h at room temperature. After washing in TBS+, sections were incubated with fixation solution (20 mM HEPES, pH 7, 60% (v/v) acetone and 3.7% formaldehyde) for 2 min. After washing in TBS+, endogenous phosphatase activity was heat-inactivated by incubation at 65°C for 1 h. Section were equilibrated in detection buffer (100 mM Tris-HCl, pH 9.5, 100 mM NaCl and 5 mM MgCl_2_) and bound AP-protein was visualized by incubation in detection buffer containing levamisole and NBT/BCIP (Roche). The specificity of RGMA-AP protein binding was determined by competition with excess RGMA protein. Furthermore, no staining was observed for AP alone.

#### In-gel analysis

Pull down samples were separated in a NuPAGE Novex 4-12% Bis-Tris gradient gel following the manufacturer's description (Invitrogen). For mass spectrometry analysis, proteins were visualized using GelCode Blue Stain Reagent (Thermo-Fisher). Silver staining was used to detect differential protein bands. The gel was soaked twice in 50% methanol, followed by a 10 min incubation in 5% methanol. After 3 rinses in water, the gel was incubated in 10 μM dithiothreitol (DTT) for 20 min, followed by 0.1% (w/v) AgNO_3_ for 20 min. The gel was washed once in water and twice in developer solution (3% (w/v) Na_2_CO_3_ and 0.02% (w/v) formaldehyde). The gel was incubated in the developer solution until protein bands appeared. The staining reaction was stopped by adding 5% (w/v) citric acid.

#### Gel digestion and nanoflow LC-MS/MS analysis

1D SDS-PAGE gel lanes were cut into 2-mm slices using an automatic gel slicer and subjected to in-gel reduction with DTT, alkylation with iodoacetamide and digestion with trypsin (Promega, sequencing grade), essentially as described previously. Nanoflow LC-MS/MS was performed on a CapLC system (Waters, Manchester, UK) coupled to a Q-TOF Ultima mass spectrometer (Waters, Manchester, UK) operating in positive mode and equipped with a Z-spray source. Peptide mixtures were trapped on a JupiterTM C18 reversed phase column (Phenomenex; column dimensions 1.5 cm × 50 μm, packed in-house) using a linear gradient from 0 to 80% B (A = 0.1 M acetic acid; B = 80% (v/v) acetonitrile, 0.1 M acetic acid) in 70 min and at a constant flow rate of 200 nl/min using a splitter. The column eluent was directly sprayed into the ESI source of the mass spectrometer. Mass spectra were acquired in continuum mode; fragmentation of the peptides was performed in data-dependent mode. Peak lists were automatically created from raw data files using the Protein Lynx Global Server software (version 2.0). The background subtraction threshold for noise reduction was set to 35% (background polynomial 5). Smoothing (Savitzky-Golay) was performed (number of interactions: 1, smoothing window: 2 channels). Deisotoping and centroiding settings were: minimum peak width: 4 channels, centroid top: 80%, TOF resolution: 5000, NP multiplier: 1. Mascot search algorithm (version 2.0, MatrixScience) was used for searching against the NCBInr database that was available on the MatrixScience server. The peptide tolerance was typically set to 150 ppm and the fragment ion tolerance was set to 0.2 Da. A maximum number of 1 missed cleavage by trypsin was allowed and carbamidomethylated cysteine and oxidized methionine were set as fixed and variable modifications, respectively.

#### *In utero* electroporation

*In utero* electroporation was performed as describe previously (e.g. van Erp et al., 2015). In brief, pregnant C57BL/6j females were anaesthetized at E14.5 with isoflurane in oxygen. The abdominal cavity was opened, uterine horns were exposed and embryos counted. 1.7 μl DNA mix always containing 0.2 μg/μl pCX-GFP (gift from Alain Chédotal), and 1 μg/μl pCMV-mycDDK-RGMA (Origene cat# MR206975) or 1 μg/μl pCAG:mNET1-/3xGS/mCherry (VectorBuilder), or 1 μg/μl RGMA with 0.5 μg/μl NET1, or 1 μg/μl NET1 with 1 μg/μl pSuper-shNEO1 or 1 μg/μl pSuper-Scrambled (van Erp et al., 2015) was injected in the lateral ventricles. Conditions were randomized among mothers by dividing all embryos in a single uterus into two to three groups. Heads were held with a 1 cm platinum tweezer electrode (Harvard apparatus) coupled to the negative pole and a third gold-plated positive electrode (Harvard apparatus) was placed on top of the midline of the head. Five 50 ms pulses of 30 V were given with an interval of 950 ms using a BTX square pulse electroporator (Harvard apparatus). Uterine horns were carefully placed back in the abdomen, and muscle and skin layers were sutured separately. 5 mg/ml/10g mouse EdU (ThermoFischer) was administered i.p. in the mother at 24 h after surgery. Embryos were isolated at E16.5 or E17.5 and brains were rapidly dissected and immersion-fixed in 4% PFA in PBS for no more than 24 h. Brains were cryoprotected in 30% sucrose and immersed in 7.5% gelatin-10% sucrose for cryostat sectioning. 20 μm cryosections were blocked in 2% gelatin and 0.1% Triton-X100 in PBS for 1 hr and primary antibody (chicken anti-GFP (1:2000, AVES) was incubated overnight at room temperature in the same buffer. Followed by incubation with appropriate secondary antibodies (donkey anti-chicken IgY 488, 1:750, Jackson Immunoresearch). EdU was revealed using the Click-It EdU Cell proliferation kit for imaging, Alexa Fluor 647 dye (ThermoFisher), according to manufacturer’s protocol. Sections were counterstained with DAPI, mounted in mowiol and imaged for quantification on a Zeiss Axiovision epifluorescent microscope with a 20x objective. For each brain, images were taken from 3 consecutive sections starting from the first section that showed a connected corpus callosum. Images were processed in Adobe Photoshop, where 3 stacks of 8 (in case of E16.5) or 4 (in case of E17.5) square bins were placed using the DAPI channel. In case of E16.5, the stack of 8 bins reached from the bottom of the subventricular zone until the top of the cortical plate. In case of E17.5, the 4 bins covered the entire cortical plate. GFP-positive or EdU-positive migrating cells in each bin were manually counted and represented as a percentage of the total number of cells in the entire stack of bins. All values of 3 bins in the 3 images per brain were averaged and this was used as a final score (n). At least 3 brains were counted per condition. Graphs were made and statistical analysis (ordinary one-way ANOVA with Sidaks multiple comparisons) was performed using Prism software. Publication images were prepared using a Zeiss LSM 880 confocal microscope with 20x objective.

#### Co-immunoprecipitation

For co-immunoprecipitation (IP) of RGMB from adult mouse brain, three cortices of adult mice were lyzed in 3 ml of lysis buffer (40 mM Tris-HCl pH 7.5, 200 mM NaCl, 10% glycerol, 5 mM EDTA, 0.4% NP-40, protease inhibitor (cOmplete, Sigma-Aldrich), phosphatase inhibitor (Cocktail 2, Sigma-Aldrich)). Lysate was incubated for 10 min at 4 °C on a rotating wheel. Then the lysate was centrifuged for 10 min and supernatant was collected. 100 μl of supernatant was kept for Western blot as input. The rest of the lysate was split into half, one of these halves was incubated with 0.5 μg of non-specific sheep IgGs (R&D Systems 5-001-A) and the other half with 0.5 μg of sheep anti-RGMB antibody (R&D AF3597), followed by incubation with 5 μl of magnetic beads (Dynabeads Protein G, Thermofisher) for 1hr at 4°C on a rotating wheel. Then, the beads were washed three times with 0.5 ml of lysis buffer. For Western blot, 5 μl of lysate was loaded on gel and in case of beads the entire content of the beads.

#### *Syn-GFP-NEO1 in vivo* immunoprecipitation

E18.5 and P0 dissected brains were lysed in 1500 μl lysis buffer (20 mM Tris-HCl, pH 7.5, 150 mM NaCl, 10% glycerol, 1% NP-40 and 200 ng/μl albumin from chick egg white (CEA; Sigma-Aldrich) and cOmplete protease inhibitor cocktail (Roche)) and incubated for 30 min at 4 °C while rotating and centrifuged at 14,000 rpm for 30 min at 4 °C. Cleared supernatants were incubated with 1 μg of rabbit anti-GFP antibody (ab290; Abcam) and incubated rotating at 4 °C. After 2 h, 10 μl protein A Dynabeads (Invitrogen), which had been blocked in blocking buffer (20 mM Tris-HCl, pH 7.5, 150 mM NaCl, 20% glycerol and 200 ng/μl CEA (Sigma-Aldrich)), was added to each sample and samples were incubated for 40 min rotating at 4 °C. Brain lysates of either 4 *Syn-GFP-NEO1* or 4 wild type littermates were pooled and beads were washed 4 times in washing buffer (20 mM Tris-HCl, pH 7.5, 150 mM NaCl, 10% glycerol and 1% NP-40). Precipitated proteins were eluted by boiling in NuPAGE LDS sample buffer (Invitrogen) with 2.5% β-mercaptoethanol for 10 min at 70 °C.

#### Preparation of mouse brain lysates

Different parts of the brain were lysed in 400 μl lysis buffer (40 mM Tris HCl pH 7.5, 200 mM NaCl, 10% glycerol, 5 mM EDTA, 0.4% NP-40, protease inhibitor (cOmplete, Sigma-Aldrich), phosphatase inhibitor (Cocktail 2, Sigma-Aldrich)). Lysates were incubated for 10 min at 4 °C on a rotating wheel. Then the lysate was centrifuged for 10 min and the supernatant was collected. For Western blot, 20 μl of supernatant was loaded on the gel.

#### Immunoblotting brain lysates

Protein samples (cell lysate, brain lysate or content of beads after IP) were loaded onto a 10% SDS-PAGE gel and proteins were transferred onto a nitrocellulose membrane (GE Healthcare Life Sciences, Amersham Protran 0.45 μm NC, 10600002) in Tris-Glycine buffer with 10% methanol. Membranes were washed with TBS/0.1% Tween and blocked with blocking buffer (5% milk (ELK, Campina) in TBS/0.1% Tween) for 30 min at room temperature. Membranes were incubated with primary antibody in blocking buffer, washed 3 x 5 min in TBS/0.1% Tween, incubated with secondary antibody diluted in TBS/0.1%Tween (1:10,000), and again washed 3 x 5 min in TBS/0.1% Tween. The peroxidase conjugated secondary antibodies were visualized using the SuperSignal (34076, ThermoFisher) reagent and FluorChem E (FE0285, Proteinsimple) imaging system. For re-staining of the membrane of the co-immunoprecipitation experiment, the membrane was incubated in stripping buffer (62.5 mM Tris-HCl, pH 6.8; 2% SDS; 0.8% beta-mercaptoethanol; in H_2_O) for 5 min at 60°C. The membrane was washed 3 times for 5 min in TBS/0.1% Tween and processed for another antibody. The membrane was first stained for NET1, then for NEO1 and last for RGMB. Primary antibodies used were sheep anti-RGMB antibody (AF3597, R&D; 1:500); rat anti-NET1 (MAB1109, R–D); goat anti-NEO1 (AF1079, R&D; 1:500); rabbit anti-GFP antibody (ab290, Abcam, 1:6000); mouse anti-FLAG (Stratagene, 1:2000); mouse anti-α-Tubulin (T5168, Sigma-Aldrich, 1:8000); rabbit anti-NEO1 antibody (sc-15337, Santa Cruz, 1:500). Secondary antibodies used were rabbit anti-sheep peroxidase conjugated (Abcepta ASR1953); donkey anti-goat peroxidase conjugated (705-035-003, Jackson ImmunoResearch); and goat anti-rat peroxidase conjugated (sc-2065, Santa Cruz), goat anti-mouse peroxidase conjugated (A0168, Merck) and goat anti-rabbit peroxidase conjugated (111-035-003, Jackson Immunoresearch).

### Quantification and Statistical Analysis

For calculations of Fourier shell correlations (FSC), the FSC cut-off criterion of 0.143 was used. The experiments were not randomized. The investigators were not blinded to allocation during experiments and outcome assessment.

For SPR, non-linear regression curve fitting was performed using GraphPad Prism (www.graphpad.com).

SEC-MALS scattering data were analyzed and molecular weight was calculated using ASTRA 6 software (Wyatt).

PLA statical analysis was carried out using a two-tailed, unpaired t-test from 3 individual, independent experiments.

Growth cone collapse was quantified for more than 100 neurons per condition, for three to six independent experiments. The criterion for the collapsed growth cones was loss of lamellipodia and the presence of only two or fewer filopodia. Statistical analysis was performed with GraphPad Prism using one-way ANOVA (or two-way ANOVA for *Emx1-Cre;DCC-flox* experiment) followed by Tukey’s multiple comparison test.

For the SVZ-NSC differentiation and proliferation assay images were analyzed using ImageJ. The single TUJ1, EdU and DAPI channels were first thresholded. Then Edu- positive, TUJ1-positive and DAPI-positive cells were counted using the Analyze Particles function in ImageJ. For both assays three biological replicates were performed (n = 3), each technical replicate contained 2 coverslips. To test the differences between conditions one-way ANOVA was performed in GraphPad Prism.

For the SVZ-NSC migration assay images were analyzed using ImageJ. Cells that were outside the neurosphere and positive for TUJ1 and the neuronal migration marker doublecortin (DCX) were considered to be migrating neurons. Each experimental group contained 9 to 18 coverslips, and per coverslip between 4 and 6 SVZ NSCs were imaged. The number of migrating cells per neurosphere per condition was normalized to the vehicle (negative control). Bartlett’s test was used to test for a significant difference between the standard deviations. When the input data did not meet the criteria for Bartlett’s test, the Brown-Forsythe test was performed to test for a significant difference between the standard deviations. For normally distributed data, one-way ANOVA (or two-way ANOVA for *Emx1-Cre;DCC-flox* experiment) and post hoc Tukey’s test were performed to test the difference between groups. For not normally distributed data Kruskal-Wallis test with Dunn’s multiple comparisons test were performed. For the RGMB assay, a paired two-tailed t-test was performed. All statistical analysis were performed using GraphPad Prism, Migration distances of DCX/TUJ1+ cells were analyzed by measuring the distance between the nucleus and the edge of the neurosphere. For the RGMA/RGMB-related assays this was measured for all migrating neurons. For the assays concerning the NET1_ΔNTR_ and NET1_WT_-Interface1 and NET1_WT_-Interface2, the migration distance of a randomly selected subset of neurons was analyzed.

For migration distance quantification of the *in utero* electroporation experiments for each brain, images were taken from 3 consecutive sections starting from the first section that showed a connected corpus callosum. Images were processed in Adobe Photoshop, where 3 stacks of 8 (in case of E16.5) or 4 (in case of E17.5) square bins were placed using the DAPI channel. In case of E16.5, the stack of 8 bins reached from the bottom of the subventricular zone until the top of the cortical plate. In case of E17.5, the 4 bins covered the entire cortical plate. GFP-positive or EdU-positive migrating cells in each bin were manually counted and represented as a percentage of the total number of cells in the entire stack of bins. All values of 3 bins in the 3 images per brain were averaged and this was used as a final score (n). At least 3 brains were counted per condition. Graphs were made and statistical analysis (ordinary one-way ANOVA with Sidaks multiple comparisons) was performed using Graphpad Prism.

Details about replicates and statistical tests can also be found in figures and corresponding figure legends.
