# Supplementary material for: Simultaneous binding of Guidance Cues NET1 and RGM blocks extracellular NEO1 signaling
Source: Cell. 2021 Apr 15;184(8):2103–2120.e31. doi: 10.1016/j.cell.2021.02.045 (PMC8063088; doi:10.1016/j.cell.2021.02.045)
Supplement: Document S1. Tables S1–S3 and Methods S1 and S2 [file mmc1.pdf]

Cell, Volume *184*

Supplemental information

Simultaneous binding of Guidance Cues NET1 and RGM blocks extracellular NEO1 signaling

Ross A. Robinson, Samuel C. Griffiths, Lieke L. van de Haar, Tomas Malinauskas, Eljo Y. van Battum, Pavol Zelina, Rebekka A. Schwab, Dimple Karia, Lina Malinauskaite, Sara Brignani, Marleen H. van den Munkhof, Özge Düdükcü, Anna A. De Ruiter, Dianne M.A. Van den Heuvel, Benjamin Bishop, Jonathan Elegheert, A. Radu Aricescu, R. Jeroen Pasterkamp, and Christian Siebold

Cell, *Volume 184*

Supplemental information

Simultaneous binding of Guidance Cues NET1 and RGM blocks extracellular NEO1 signaling

Ross A. Robinson, Samuel C. Griffiths, Lieke L. van de Haar, Tomas Malinauskas, Eljo Y. van Battum, Pavol Zelina, Rebekka A. Schwab, Dimple Karia, Lina Malinauskaite, Sara Brignani, Marleen H. van den Munkhof, Özge Düdükcü, Anna A. De Ruiter, Dianne M.A. Van den Heuvel, Benjamin Bishop, Jonathan Elegheert, A. Radu Aricescu, R. Jeroen Pasterkamp, and Christian Siebold

Table S1: Crystallographic Data Collection and Refinement Statistics. Related to Figure 1, 2 and 3.

	**Ternary NEO1-NET1-RGMB complex**	**Binary NEO1-NET1 complex**
**Data collection**		
Beamline	DLS-I03	DLS-I03
Space group	R32	C2
Cell dimensions<br>a, b, c (Å)<br>α, β, γ (º)	 136.8, 136.8, 430.1<br>90.0, 90.0, 120.0	 160.0, 49.6, 157.3<br>90.0, 99.4, 90.0
Wavelength (Å)	0.9763	0.9763
Resolution (Å)	79.62-3.25 (3.31-3.25)	77.57-3.15 (3.23-3.15)
No. unique reflections	24856 (1184)	19817 (1376)
Completeness (%)	99.9 (99.6)	97.5 (94.4)
Multiplicity	8.1 (8.1)	3.3 (2.8)
$\langle I/\sigma(\mathrm{I})\rangle$	16.7 (1.2)	6.6 (1.0)
R_{merge} (%)	8.8 (>100)	11.9 (93.4)
R_{pim} (%)	4.6 (>100)	11.8 (92.2)
$CC_{1/2}$	0.999 (0.321)	0.993 (0.336)
CC*	1.000 (0.732)	0.998 (0.707)
**Refinement**		
Resolution	3.37-3.25	3.26-3.15
No. reflections (test set)	24845 (1276)	19809 (1015)
R_{work} / R_{free} (%)	25.0/26.4	21.2/23.9
CC_{work}	0.88 (0.58)	0.90 (0.55)
CC_{free}	0.89 (0.42)	0.92 (0.51)
No. atoms:<br>Protein<br>Ligand/Ion	 6971<br>282	 5654<br>124
B factors (Å^2):<br>Protein<br>Ligand/Ion	 161<br>211	 114<br>164
RMSD bond lengths (Å)	0.010	0.011
RMSD bond angles (º)	1.67	1.53
Ramachandran plot (%)<br>Favoured<br>Allowed<br>Outliers	 96.2<br>3.8<br>0	 95.7<br>4.3<br>0

Values in parentheses correspond to the highest resolution shell unless otherwise stated. RMSD: Root Mean Square Deviation. R_{PIM}: precision-indicating merging. R-factor. Rmerge: merged R-factor. $CC_{1/2}$, CC*: correlation coefficients between random half data sets. CC_{WORK}/CC_{FREE}: standard and cross-validated correlations of the experimental intensities with the intensities calculated from the refined molecular model.

Table S2. Cryo-EM data collection and structure refinement of the NEO1-NET1-RGMB complex. Related to Figure 2.

	Ternary NEO1-NET1-RGMB complex
**Data collection**	
Microscope	Titan Krios with Volta phase plate
Nominal magnification	96000×
Voltage (kV)	300
Detector	FEI Falcon III direct electron detector
Pixel size (Å)	0.85
Electron exposure (e^{-}/Å^{2})	40.0
Exposure length (s)	37.26
Number of fractions	40
Defocus (μm)	-0.5, -0.7
Micrographs collected (no.)	1635
Initial particle images (no.)	280158
Final particle images (no.)	68541
Symmetry imposed	C3
Map resolution (Å)<br>FSC threshold	6.0<br>0.143
**Refinement**	
Model composition<br>Protein residues<br>Non-hydrogen atoms<br>Protein atoms<br>…N-Acetylglucosamine<br>Calcium atoms	 2643<br>20934<br>20763<br>168<br>3
R.m.s. deviations<br>Bond lengths (Å)<br>Bond angles (°)	 0.011<br>1.508
Validation<br>MolProbity score<br>Clash score<br>Poor rotamers (%)	 1.41<br>1.87<br>1.82
Ramachandran plot<br>Favored (%)<br>Allowed (%)<br>Disallowed (%)	 96.16<br>3.84<br>0.00

Table S3. NEO1-interacting proteins identified in the anti-GFP *in vivo* NEO1 proteomics screen. Related to Figure 6 and 7.

				GFP-Neogenin		
**#**	**Protein name**	**Acronym**	**MW (kDa)**	**Mascot score**	**Unique peptides**	**Biological function**
1	**Neogenin**	**NEO1**	**160**	**661**	**16**	**axon guidance; cell adhesion; migration**
2	Desmin	DES	53	227	5	actin cytoskeleton organization; type III intermediate filament; neuromuscular junction
3	RNA binding motif protein 14	RBM14	69	276	6	RNA transcription
4	Doublecortin-like kinase 2	DCLK1	47	248	6	protein serine/threonine kinase activity; cell differentiation; axon outgrowth; dendrite morphogenesis; neuron migration
5	Actin binding LIM protein family, member 3	ABLIM3	78	236	5	actin cytoskeleton organization; axon guidance; regulation of transcription
6	Topoisomerase (DNA) II beta	TOP2B	182	220	5	DNA binding; neuromuscular junction; neuron migration; neuronal differentiation; neurite outgrowth; corticogenesis
7	Actin binding LIM protein 1	ABLIM1	97	201	6	actin cytoskeleton organization; axon guidance; transcription regulation
8	Cortactin	CTTN	61	188	6	actin cytoskeleton organization; dendritic spine morphogenesis; cell migration; cell adhesion junctions
9	Calcium/calmodulin-dependent protein kinase II beta	CAMK2B	61	178	4	protein serine/threonine kinase activity; regulation of long-term neuronal plasticity; regulation of neuron projection development; apoptosis; Wnt signaling
10	Interleukin enhancer binding factor 2	ILF2	43	170	4	DNA binding; RNA binding; regulation of transcription
11	Interleukin enhancer binding factor 3	ILF3	96	126	4	DNA binding; RNA binding; regulation of transcription; immune response
12	Casein kinase 2A	CSNK2A1	45	125	4	ATP binding, beta-catenin binding, axon guidance, protein serine/threonine kinase activity, cell cycle, signal transduction
13	Metastasis associated 1	MTA1	79	104	3	DNA binding; regulation of transcription; Wnt signaling
14	Metastasis associated 1 family, member 2	MTA2	75	104	3	DNA binding; regulation of transcription; Wnt signaling
15	RAN binding protein 10	RANBP10	70	104	3	Ran GTPase binding; beta-tubulin binding; microtubule cytoskeleton organization
16	Dedicator of cytokinesis 7	DOCK7	238	102	4	Rac GTPase binding; microtubule cytoskeleton organization; axonogenesis; cell polarity; Schwann cell differentiation and myelination

17	Nitric oxide synthase 1 (neuronal) adaptor protein	NOS1AP	56	100	2	Nitric-oxide synthase binding; link Abl family kinases and the actin cytoskeleton
18	Nucleolin	NCL	77	99	2	DNA binding; RNA binding
19	Actin filament associated protein 1	AFAP1	81	93	2	actin binding
20	Cytoplasmic linker associated protein 1	CLASP1	169	88	3	actin and microtubule cytoskeleton organization; axon guidance
21	Regulating synaptic membrane exocytosis 1	RIMS1	163	88	4	Rab GTPase binding; exocytosis; neurotransmitter transport; regulation of long-term synaptic neuronal plasticity
22	Contactin 1	CTN1	113	80	2	cell adhesion; axon guidance
23	Neuron navigator 1	NAV1	202	80	2	cell differentiation; microtubule cytoskeleton organization
24	Metastasis associated 1 family, member 3	MTA3	66	78	2	DNA binding; regulation of transcription; Wnt signaling
25	Lysosomal trafficking regulator	LYST	425	76	3	endosome to lysosome transport
26	Molecule interacting with CasL 1	MICAL1	109	75	2	actin cytoskeleton organization; microtubule cytoskeleton organization; regulation of protein phosphorylation; apoptosis
27	CDC42 binding protein kinase alpha (DMPK-like)	CDC42BPA	197	72	3	protein serine/threonine kinase activity; actin cytoskeleton organization; cell migration; signal transduction; microtubule cytoskeleton organization
28	Dynactin 1	DCTN1	140	70	2	microtubule cytoskeleton organization; axonogenesis
29	Nuclear mitotic apparatus protein 1	NUMA1	236	70	3	microtubule binding; mitotic spindle orientation
30	Valosin-containing protein	VCP	89	68	2	ATPase activity; ER to Golgi vesicle-mediated transport; regulation of protein complex assembly; apoptosis
31	CDC5 cell division cycle 5-like (*S. pombe*)	CDC5L	92	68	3	DNA binding; RNA binding; regulation of transcription
32	Leucine rich repeat (in FLII) interacting protein 2	LRRFIP2	47	67	2	Wnt receptor signaling

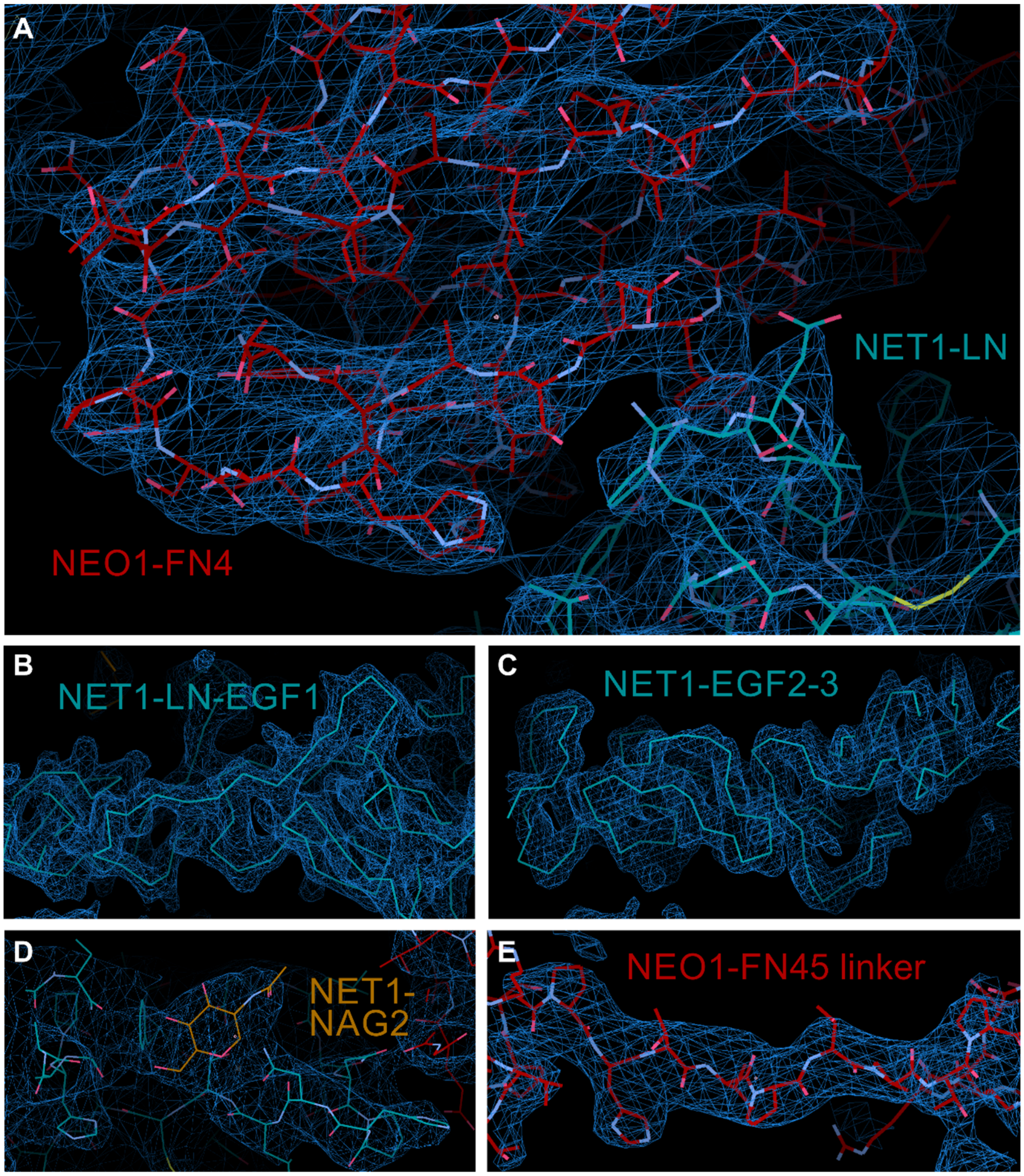

Methods S1. SigmaA-weighted 2Fo-Fc maps of the NEO1-NET1-RGMB complex from the final round of refinement in AutoBuster. Related to Figure 1 and Star Methods. (A) NEO1-FN4:NET1-LN interface. **(B)** The base of the NET1 LN head and EGF1. **(C)** NET1 EGF2-3. **(D)** N-acetyl-glucosamine 1 (see Fig. S9) on the NET1 surface. **(E)** The ordered linker region between NEO1 FN repeats 4 and 5. All models and maps were displayed using COOT, with maps contoured at 1σ.

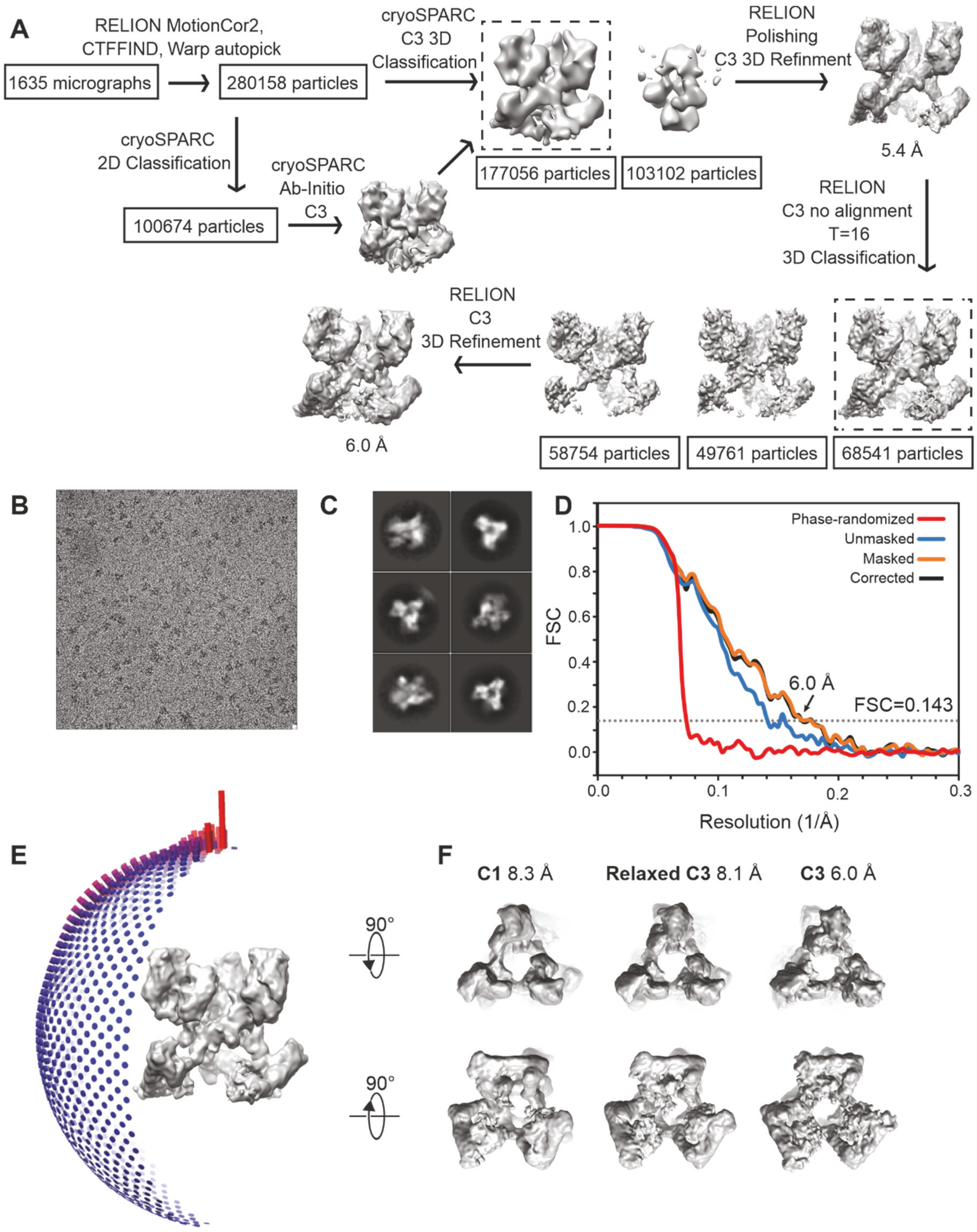

Methods S2. Cryo-EM image processing procedure for the ternary NEO1$_{FN456}$-NET1-RGM complex. Related to Figure 2 and Star Methods. (A) Graphical overview of cryo-EM data collection and image processing using RELION 3.1 and cryoSPARC v2. In short, drift correction, beam-induced motion and dose-weighting were done with MotionCor2 in RELION 3.1, particle automated picking was performed in Warp. Representative aligned, dose-weighted

micrograph depicted in (**B**) and representative 2D class averages in (**C**). Ab-initio 3D model was generated with cryoSPARC v2 from best selected 2D class average 100674 particles. cryoSPARC v2 3D classification was performed with all picked particles and best class with 177056 particles imported in RELION 3.1, performed 3D refinement and Bayesian particle polishing. 3D classification without alignment with regularisation parameter set to T=16 gave one class with more continuous map for RGMB, which was refined to 6.0 Å resolution, as estimated using the Fourier shell correlation (FSC) = 0.143 criterion (dashed line) (**D**). Curves are shown for the phase randomized (red), unmasked (blue), masked (orange) and phase-randomization-corrected masked (black) maps. **(E)** Angular-distribution histogram of particles projections used in calculating the final 3D reconstruction of the complex. **(F)** Maps comparison refined with C3 symmetry (right panel), with C1 (left panel) and relaxed C3 symmetry (middle panel) in RELION 3.1.
